# Supplementary material for: Use of locum doctors in NHS trusts in England: analysis of routinely collected workforce data 2019–2021
Source: BMJ Open. 2023 May 15;13(6):e065803. doi: 10.1136/bmjopen-2022-065803 (PMC10254888; doi:10.1136/bmjopen-2022-065803)
Supplement: Supplementary data [file bmjopen-2022-065803supp001.pdf]

Supplementary material - The use of locum doctors in NHS Trusts in England. Analysis of routinely collected workforce data 2019 – 2021.

Contents

Additional databases ... 3

NHS Workforce statistics database ... 3

NHS Vacancy statistics ... 3

Health Regulators Ratings ... 3

English Indices of Deprivation ... 4

Spatial Maps ... 4

Tables ... 5

$Table 1: NHS Trusts with the highest and lowest locum intensity in England, 2019 ... 5

$Table 2 - Descriptive statistics in 2020, by region*† ... 6

$Table 3 - Descriptive statistics in 2021, by region*† ... 7

$Table 4: Negative binomial regression analyses for the three outcomes in 2019, acute and mental health trusts, IRR a,b,c ... 8

$Table 5: Adjusted marginal effects for the three outcomes in 2019, acute and mental health trusts, a,b ... 10

Figures ... 11

$Figure 1: Locum intensity, proportion of agency shifts and proportion of unfilled shifts in 2019, 2020 & 2021 - NHS Trust level ... 11

$Figure 2: Regional variation in locum intensity, proportion of agency shifts and proportion of unfilled shifts, 2019, 2020 & 2021 - NHS Trust level* ... 12

$Figure 3: Spatial map of locum intensity at the Sustainability and Transformation Partnerships (STP) level, England 2019 ... 13

$Figure 4: Spatial map of agency shifts at the Sustainability and Transformation Partnerships (STP) level, England 2019 ... 14

$Figure 5: Spatial map of unfilled shifts at the Sustainability and Transformation Partnerships (STP) level, England 2019 ... 15

References ... 16

Additional databases

NHS Workforce statistics database

NHS Digital collects monthly NHS Hospital and Community Health Service (HCHS) workforce statistics (1) for staff in NHS trusts and Clinical Commissioning Groups (CCGs) in England. The data are recorded within the Electronic Staff Record (ESR), which is a payroll and human resources system and contains staff records NHS employed staff in England since 2008. Data are available as headcount and full-time equivalents and for all months from September 2009 onwards and they represent an accurate summary of the validated data extracted from the NHS ESR system. We downloaded monthly data collections available from NHS Digital from December 2017 to December 2021. For each month and each NHS trust, we calculated the average total FTE across all available doctor categories in the NHS workforce database and matched the data to NHS improvement data for each trust in each month. The database also included monthly NHS trust level turnover data which were used in the negative binomial regression.

NHS Vacancy statistics

From NHS Digital, (2) we obtained trust level vacancy rates for the period January 2019 to December 2019. The series refers to vacancy FTE from providers which are available on a quarterly basis but recorded on a monthly basis. This monthly rate is defined as the total number of unfilled posts reported at the end of each respective reporting month. A vacancy is defined as a post that is unfilled by permanent or fixed-term staff. Some vacant posts may be filled by agency or temporary staff, but these posts are still considered to be vacancies. The number of vacancies is the difference between the number of reported full-time equivalent (FTE) permanent or fixed-term staff in post and planned workforce levels (i.e. the total funded or budgeted establishment on an FTE basis).

Health Regulators Ratings

Health regulators ratings were obtained from the Care Quality Commission (CQC). Each rating is based on the assessment of the evidence against the key lines of enquiry in the assessment framework for healthcare services and, for relevant non-specialist acute trusts, the use of resources assessment framework. The trust level ratings refer to the trust's overall quality, based on findings under five key quality questions that CQC inspects (safe, effective, caring, responsive and well-led). Where applicable, the CQC also awards a combined rating at the trust level, based on the findings of the five trust-level quality ratings plus a use of resources rating. (3, 4) Each year the CQC inspects NHS trusts,

and awards each of them one of four rating levels ('outstanding', 'good', 'requires improvement', or 'inadequate') in five domains ('safe', 'effective', 'responsive', 'caring', and 'well led'), along with an 'overall' rating that summarised the domain ratings. From the CQC, we obtained published inspection ratings and corresponding inspection dates for all 229 NHS trusts in 2019 that were inspected between January 2019 and December 2019. Over the inspection cycle, some NHS trusts were re-inspected and their ratings updated. Only the rating from the first inspection was used in this study, as subsequent ratings were likely to have been influenced by the previous inspection process and outcomes. Data on the most recent practice inspections are freely available online. (5)

English Indices of Deprivation

Area deprivation, as measured by the latest update of the Index of Multiple Deprivation (IMD) (i.e.2019) was available at the 2011 Lower Super Output (LSOA) level. The IMD measures relative levels of deprivation for all the 32,844 LSOAs in England on a continuous scale of deprivation where most of the indicators are based on 2012 statistics. It is a combined score of deprivation based on a total of 37 separate indicators that have been grouped into seven domains, each of which reflects a different aspect of deprivation experienced by individuals living in an area. The overall measure is calculated as a weighted mean across seven domains: income, employment, education and skills, health and disability, crime, barriers to housing and services, and living environment with different weights given to each domain. The Index of Multiple Deprivation is assigned to every small area in England and ranks them from 1 (most deprived area) to 32,844 (least deprived area). The IMD is widely used across central government to focus programmes on the most deprived areas. (6) These area measures were assigned to trust based on trusts' location. Trusts in our analysis are allocated an IMD score based on the mean IMD score of all admitted patients using data from Hospital Episode Statistics 2017. (7)

Spatial Maps

Digital vector boundaries for the 2019 STPs (Sustainability and Transformation Partnerships), generalised to 20 metres and clipped to the coastline to reduce size and improve visualisation, were obtained from the ONS open geography portal. (8) The vector boundaries were inputted in the Stata shp2dta (9) command to calculate the centroid for each STP in the British National Grid format. These were then converted from British National Grid easting and northing to longitude and latitude in degrees. (10)

Tables

$Table 1: NHS Trusts with the highest and lowest locum intensity in England, 2019

**Trusts with the highest locum intensity in England**	**Locum intensity***	**Trusts with the lowest locum intensity in England**	**Locum intensity***
North East London NHS Foundation Trust	0.795	Royal Papworth Hospital NHS Foundation Trust	0.001
Bedfordshire Hospitals Foundation Trust	0.768	Cambridgeshire Community Services NHS Trust	0.002
Rotherham Doncaster And South Humber NHS Foundation Trust	0.684	The Newcastle Upon Tyne Hospitals NHS Foundation Trust	0.004
Oxford Health NHS Foundation Trust	0.621	Moorfields Eye Hospital NHS Foundation Trust	0.009
Dudley And Walsall Mental Health Partnership NHS Trust	0.602	Alder Hey Children's NHS Foundation Trust	0.010
North Cumbria University Hospitals NHS Trust	0.595	North Tees And Hartlepool NHS Foundation Trust	0.014
George Eliot Hospital NHS Trust	0.591	University Hospitals Bristol And Weston NHS Foundation Trust	0.014
North Cumbria Integrated Care NHS Foundation Trust	0.551	Sheffield Children's NHS Foundation Trust	0.022
United Lincolnshire Hospitals NHS Trust	0.548	Leeds Community Healthcare NHS Trust	0.025
Pennine Acute Hospitals NHS Trust	0.548	Imperial College Healthcare NHS Trust	0.029

*Locum intensity is defined as the sum agency and bank locum shifts adjusted for the size of Trusts' permanent doctor workforce

$Table 2 - Descriptive statistics in 2020, by region*†

	East of England	London	Midlands	North East & Yorkshire	North West	South East	South West
Locum intensity *, Median (25th–75th centile)	**0.21 (0.11 to 0.29)**	**0.17 (0.08 to 0.27)**	**0.23 (0.11 to 0.37)**	**0.16 (0.06 to 0.29)**	**0.19 (0.10 to 0.34)**	**0.18 (0.06 to 0.28)**	**0.11 (0.08 to 0.18)**
Proportion of agency shifts (%) Median (25th–75th centile)	**62.2 (32 to 94.4)**	**42.1 (18.7 to 77.5)**	**74.1 (47.2 to 95.1)**	**63.5 (44.4 to 100)**	**57.2 (36.1 to 93.9)**	**50 (24.3 to 94.7)**	**67.4 (33 to 100)**
Proportion of unfilled shifts † (% of requested shifts), Median (25th–75th centile)	**4.87 (0 to 16)**	**12 (1.15 to 21.7)**	**2.3 (0 to 14.4)**	**4 (0 to 21.1)**	**6.7 (0 to 18.7)**	**5.6 (0 to 18)**	**5 (0 to 20.9)**
Full-time doctor FTE, Median (25th–75th centile)	628.7 (288.3 to 1,344.9)	846 (379.3 to 2,102.7)	593.2 (233.2 to 1,347.8)	715.6 (269 to 1,479.1)	603.2 (309.2 to 1,032.1)	1,037.6 (293.9 to 1,392.8)	743.2 (240.9 to 1,249)
Trust types							
NHS general acute trusts (n)	16	18	20	21	20	17	17
Acute - NHS specialist trusts (n)	1	5	3	1	6	1	-
Mental health trusts (n)	4	10	12	8	6	5	6
Community health (n)	3	2	4	1	2	5	-
Ambulance service (n)	1	1	2	2	1	2	1

$Table 3 - Descriptive statistics in 2021, by region*†

	East of England	London	Midlands	North East & Yorkshire	North West	South East	South West
Locum intensity *, Median (25th–75th centile)	**0.19 (0.09 to 0.28)**	**0.19 (0.09 to 0.31)**	**0.29 (0.15 to 0.40)**	**0.18 (0.07 to 0.33)**	**0.23 (0.13 to 0.39)**	**0.22 (0.14 to 0.30)**	**0.12 (0.10 to 0.19)**
Proportion of agency shifts (%) Median (25th–75th centile)	**55.4 (31 to 87.7)**	**35.8 (18.5 to 68.5)**	**70.1 (45.5 to 93.6)**	**60.3 (39.4 to 100)**	**61.5 (33.9 to 88)**	**39.6 (21.6 to 66.6)**	**55.4 (33.6 to 100)**
Proportion of unfilled shifts † (% of requested shifts), Median (25th–75th centile)	**7.66 (0 to 17.9)**	**17 (6.9 to 27.1)**	**3.7 (0 to 17.2)**	**9.6 (0 to 22.9)**	**11.9 (0 to 27.7)**	**13.1 (0 to 22)**	**8.8 (0 to 28.9)**
Full-time doctor FTE, Median (25th–75th centile)	644.5 (34.6 to 1,222.1)	776.4 (50.6 to 1,685.4)	344.3 (33.3 to 1,222.5)	654.1 (2.19 to 1,248.5)	534.2 (218.1 to 1,047.1)	438.4 (95.6 to 1,332.6)	636.7 (134.1 to 1,354.7)
Trust types							
NHS general acute trusts (n)	14	18	19	23	18	17	16
Acute - NHS specialist trusts (n)	1	5	3	1	6	1	-
Mental health trusts (n)	6	8	12	10	6	5	5
Community health (n)	3	2	4	1	2	5	-
Ambulance service (n)	1	1	1	4	-	2	1

$Table 4: Negative binomial regression analyses for the three outcomes in 2019, sensitivity analyses with acute and mental health trusts only, IRR a,b,c

	**Locum Intensity**	**Agency shifts**	**Unfilled shifts**
**Trust level aggregate FTE**<br>**(reference group is quintile 1)**	**Reference group**	**Reference group**	**Reference group**
Quintile 2	0.499 (0.369 to 0.676), <0.001 (0.077)	1.044 (0.811 to 1.344), <0.735 (0.134)	2.189 (0.967 to 4.960), <0.060 (0.913)
Quintile 3	0.465 (0.321 to 0.676), <0.001 (0.088)	1.044 (0.760 to 1.434), <0.787 (0.169)	3.076 (1.179 to 8.023), <0.022 (1.504)
Quintile 4	0.486 (0.329 to 0.716), <0.001 (0.096)	0.937 (0.671 to 1.311), <0.708 (0.160)	2.572 (0.937 to 7.063), <0.067 (1.325)
Quintile 5	0.248 (0.157 to 0.390), <0.001 (0.057)	0.859 (0.574 to 1.287), <0.462 (0.177)	3.783 (1.237 to 11.572), <0.020 (2.158)
**Trust type**<br>**(reference group is NHS non-specialist trust)**	**Reference group**	**Reference group**	**Reference group**
NHS specialist trust	0.254 (0.176 to 0.366), <0.001 (0.047)	1.600 (1.153 to 2.220), <0.005 (0.268)	0.321 (0.119 to 0.866), <0.025 (0.162)
Mental health trust	0.991 (0.643 to 1.233), <0.487 (0.147)	1.556 (1.180 to 2.052), <0.002 (0.219)	1.015 (0.457 to 2.256), <0.971 (0.414)
**CQC ratings**<br>**(reference group is trusts that provide good and outstanding services)**	**Reference group**	**Reference group**	**Reference group**
Inadequate and requiring improvement	1.626 (1.365 to 1.938), <0.001 (0.145)	1.039 (0.899 to 1.201), <0.601 (0.077)	1.329 (0.855 to 2.066), <0.206 (0.299)
**Index of multiple deprivation**			

**(reference group is quintile 1, where 1 is the most deprived)**			
Quintile 2	1.072 (0.818 to 1.406), <0.610 (0.148)	1.077 (0.859 to 1.349), <0.520 (0.124)	0.585 (0.308 to 1.111), <0.101 (0.191)
Quintile 3	1.138 (0.855 to 1.514), <0.375 (0.166)	1.182 (0.936 to 1.494), <0.159 (0.141)	0.494 (0.248 to 0.986), <0.046 (0.174)
Quintile 4	1.153 (0.855 to 1.554), <0.350 (0.175)	1.198 (0.933 to 1.539), <0.156 (0.153)	0.948 (0.448 to 2.008), <0.890 (0.363)
Quintile 5	1.038 (0.741 to 1.455), <0.827 (0.179)	1.169 (0.882 to 1.550), <0.278 (0.168)	0.628 (0.286 to 1.381), <0.247 (0.252)
**Region (reference region is London)**	**Reference group**	**Reference group**	**Reference group**
South West	0.468 (0.328 to 0.668), <0.001 (0.085)	1.595 (1.198 to 2.123), <0.014 (0.233)	1.258 (0.510 to 3.103), <0.618 (0.580)
South East	0.746 (0.531 to 1.048), <0.092 (0.130)	1.498 (1.136 to 1.975), <0.006 (0.211)	0.578 (0.262 to 1.273), <0.174 (0.233)
Midlands	0.980 (0.718 to 1.337), <0.896 (0.155)	1.481 (1.149 to 1.908), <0.001 (0.192)	0.795 (0.371 to 1.700), <0.553 (0.308)
East of England	0.815 (0.579 to 1.147), <0.240 (0.142)	1.632 (1.233 to 2.161), <0.001 (0.234)	0.419 (0.177 to 0.988), <0.047 (0.184)
North West	0.917 (0.646 to 1.301), <0.627 (0.163)	1.289 (0.964 to 1.724), <0.101 (0.191)	1.176 (0.508 to 2.724), <0.704 (0.504)
North East and Yorkshire	0.703 (0.491 to 1.005), <0.053 (0.128)	1.450 (1.084 to 1.941), <0.001 (0.215)	0.753 (0.299 to 1.901), <0.549 (0.356)
constant	0.381 (0.221 to 0.656), <0.001 (0.105)	0.339 (0.219 to 0.524), <0.001 (0.075)	0.065 (0.017 to 0.247), <0.001 (0.045)

[a] All models included data on 187 trusts (observations) with robust standard errors.
[b] 95% confidence intervals are in brackets; results are reported as incidence rate ratios (IRR) followed by *P*-values and standard errors in parentheses.
[c] Coefficients can be interpreted as proportionate changes, for example, trusts in the North West had on average 8.3% lower locum intensity than trusts in London.

$Table 5: Adjusted marginal effects for the three outcomes in 2019, acute and mental health trusts, IRR a,b

	Total shifts	Agency shifts	Unfilled shifts
**CQC rates**			
Good + outstanding	170.5 (130 to 211.1), <0.001 [20.7]	-	-
Requiring improvement + inadequate	255 (197.1 to 312.9), <0.001 [29.6]	-	-
**Trust type**			
NHS non-specialist	213.8 (168.9 to 258.6), <0.001 [22.9]	94.1 (86 to 102.3), <0.001 [4.17]	31 (22.6 to 39.4), <0.001 [4.31]
NHS specialist trust	61 (31.5 to 90.6), <0.001 [15.1]	142.2 (98.3 to 186), <0.001 [22.4]	7.23 (0.53 to 14), <0.001 [3.42]
Mental health trust	206.5 (122.7 to 290.3), <0.001 [42.8]	148.3 (112.4 to 184.3), <0.001 [18.3]	-
Ambulance service	11,829.7 (1,372.9 to 22,286.5), <0.001 [5,335.2]	3.2 (-1.51 to 7.82], <0.185 [2.4]	-
Community service	308.4 (127.5 to 489.3), <0.001 [92.3]	-	-
**Trust size**			
Quintile 1	452.9 (201.3 to 704.6), <0.001 [128.3]	-	-
Quintile 2	355.3 (196.8 to 513.8), <0.001 [80.9]	-	-
Quintile 3	224.8 (150.9 to 298.7), <0.001 [37.7]	-	-
Quintile 4	276.9 (193 to 360.8), <0.001 [42.8]	-	-
Quintile 5	157.3 (111.2 to 203.5), <0.001 [23.6]	-	-

[a] Model A included data on 220 trusts (observation) while models B and C included data on 214 trusts with robust standard errors.
[b] Coefficients can be interpreted as absolute changes, for example, trusts that were rated as inadequate and requiring improvement on average had 84.5 more weekly locum shifts than trusts that were rated as having good and outstanding services.

Figures

$Figure 1: Locum intensity, proportion of agency shifts and proportion of unfilled shifts in 2019, 2020 & 2021 - NHS Trust level

a)Mean locum intensity in 2019†*

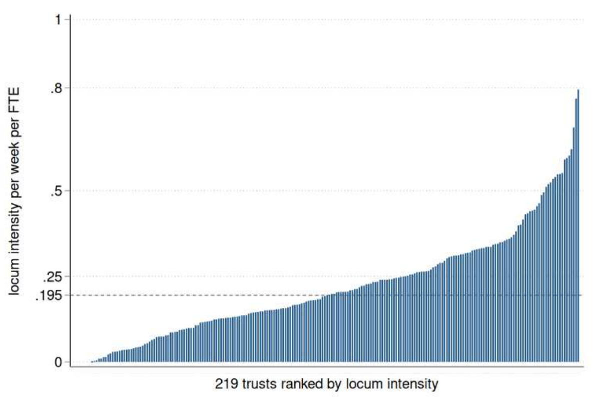

† dash line indicates the median (0.195) locum intensity per week per FTE across 219 NHS trusts in 2019.

d)Mean proportion of agency shifts in 2019††

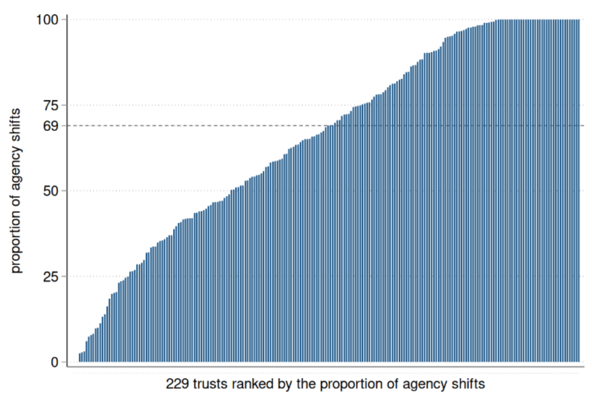

†† dash line indicates the median (69%) proportion of agency shifts perweek across 229 NHS trusts in 2019.

g)Mean proportion of unfilled shifts in 2019¥

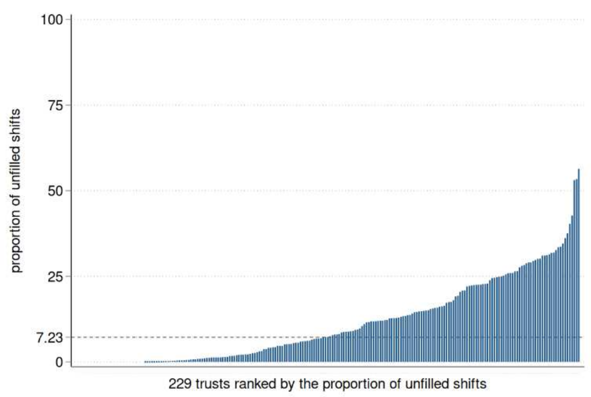

¥ dash line indicates the median (7.23%) unfilled shift rate per week across 229 NHS trusts in 2019.

b)Mean locum intensity in 2020†*

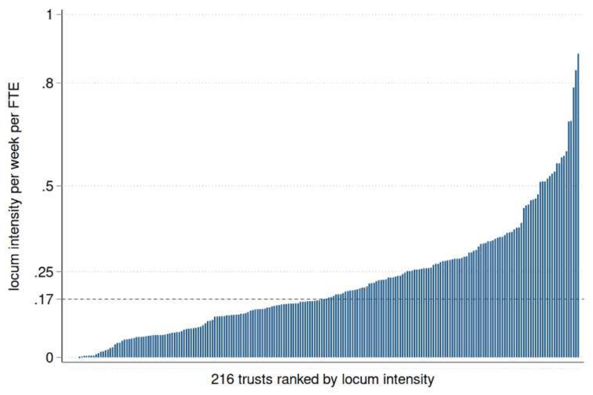

† † dash line indicates the median (0.170) locum intensity per week per FTE across 216 NHS trusts in 2020.

e)Mean proportion of agency shifts in 2020††

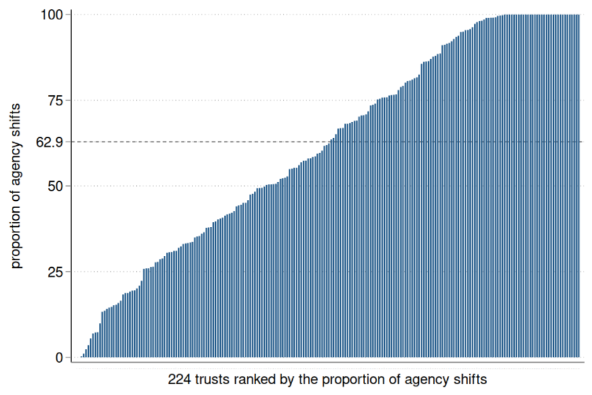

†† dash line indicates the median (62.9%) proportion of agency shifts per week across 224 NHS trusts in 2020.

h)Mean proportion of unfilled shifts in 2020¥

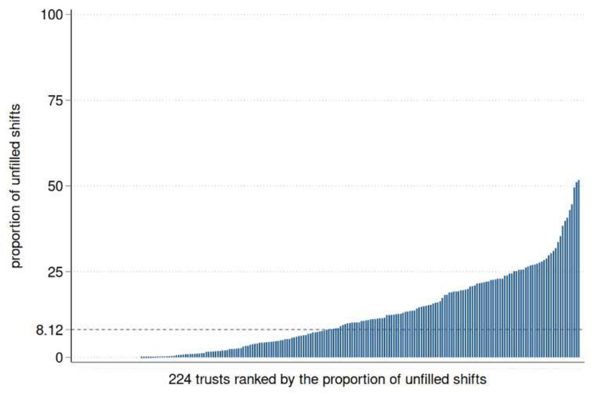

¥ dash line indicates the median (8.12%) unfilled shift rate per week across 224 NHS trusts in 2020.

c)Mean locum intensity in 2021†*

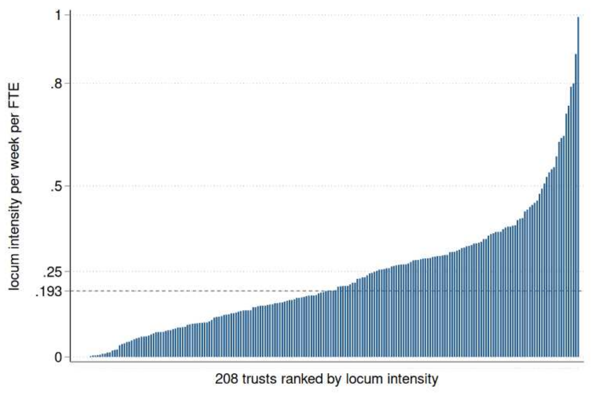

† dash line indicates the median (μ=0.193) locum intensity per week per FTE across 208 NHS trusts in 2021.

f)Mean proportion of agency shifts in 2021††

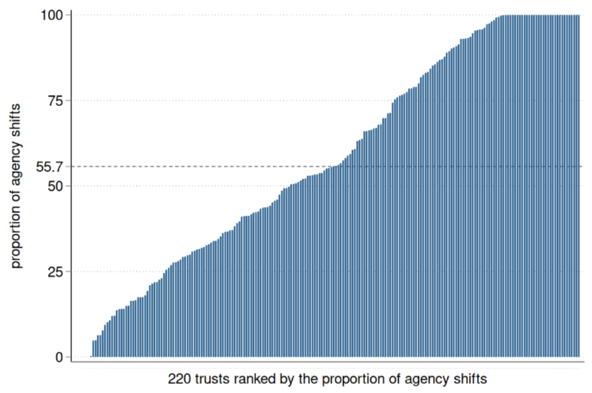

†† dash line indicates the median (μ=55.7%) proportion of agency shifts per week across 221 NHS trusts in 2021.

i)Mean proportion of unfilled shifts in 2021¥

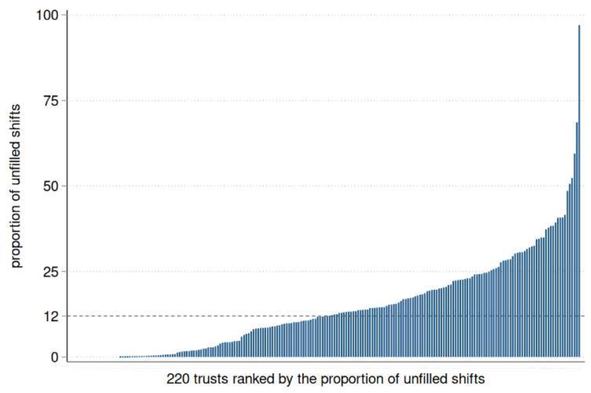

¥ dash line indicates the median (μ=12%) unfilled shift rate per week across 221 NHS trusts in 2021.

*Ten, eight and thirteen trusts were excluded from the analyses of locum intensity due to very small or zerodenominators (i.e. low permanent doctor FTE) in 2019, 2020 and 2021, respectively.

$Figure 2: Regional variation in locum intensity, proportion of agency shifts and proportion of unfilled shifts, 2019, 2020 & 2021 - NHS Trust level*

a) Regional variation in locum intensity in 2019†**

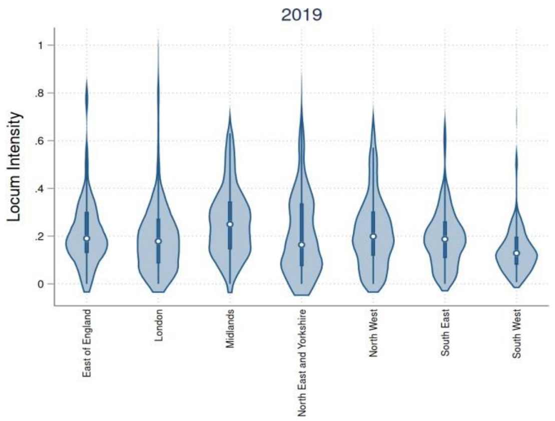

† Figure includes data from 219 NHS trusts in 2019

b) Regional variation in the proportion of agency shifts in 2019††

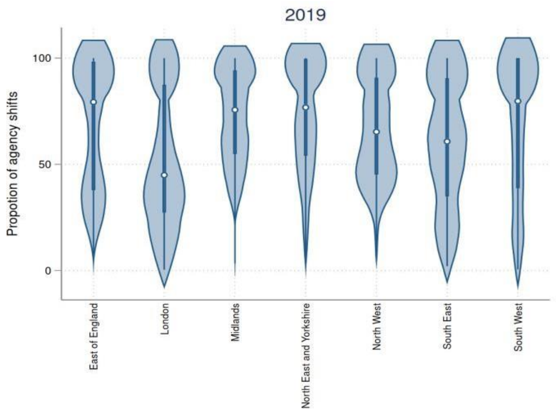

†† Figure includes data from 229 NHS trusts in 2019

c) Regional variation in the proportion of unfilled shifts in2019¥

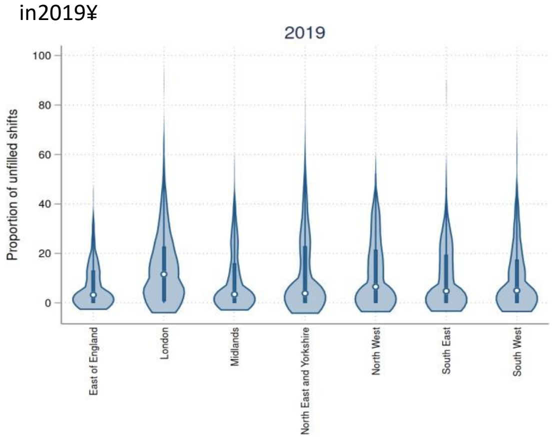

¥ Figure includes data from 229 NHS trusts in 2019

d) Regional variation in locum intensity in 2020†**

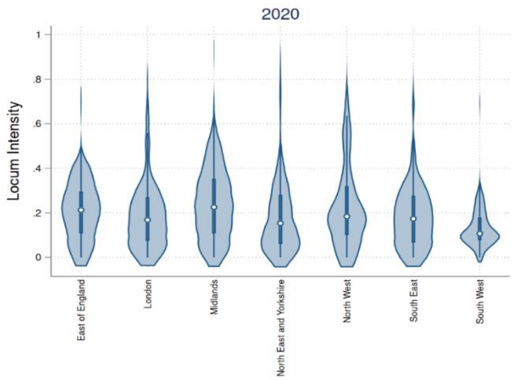

† Figure includes data from 216 NHS trusts in 2020

e) Regional variation in the proportion of agency shifts in 2020††

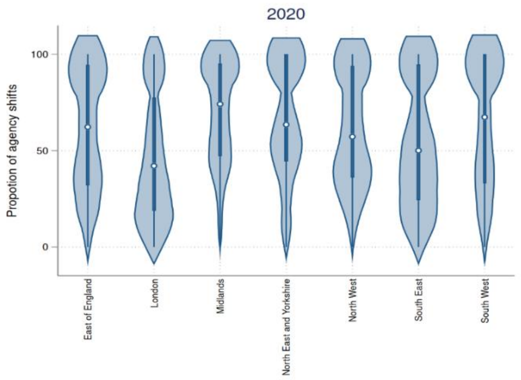

†† Figure includes data from 224 NHS trusts in 2020

f) Regional variation in the proportion of unfilled shifts in 2020¥

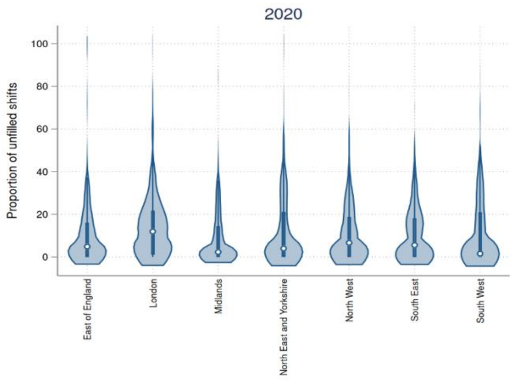

¥ Figure includes data from 224 NHS trusts in 2020

g) Regional variation in locum intensity in 2021†**

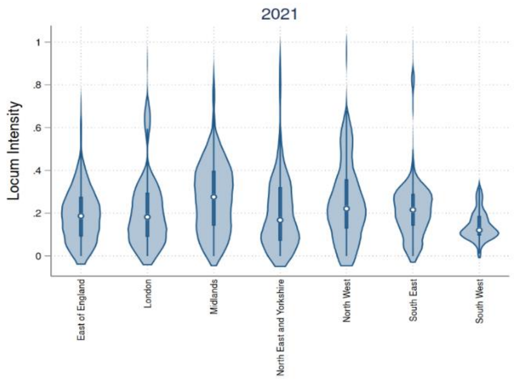

† Figure includes data from 208 NHS trusts in 2021

h) Regional variation in the proportion of agency shifts in 2021††

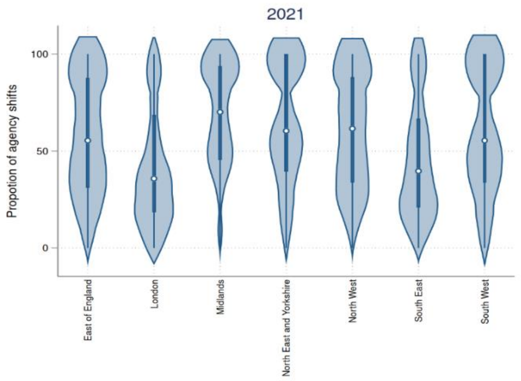

†† Figure includes data from 221 NHS trusts in 2021

i) Regional variation in the proportion of unfilled shifts in 2021¥

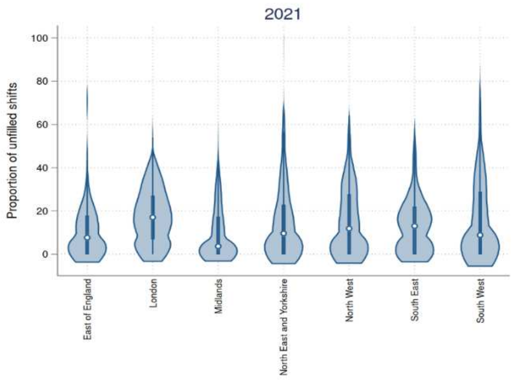

¥ Figure includes data from 221 NHS trusts in 2021

* The thick blue line represents the interquartile range (25th–75th centile) and the thin line represents the rest of the distribution with upper/lower adjacent values. The white dot represents the median of the data.The distribution shape of the data is based on a kernel density estimation where wider sections of the plot represent a higher chance that members of the population of interest will take on a given value and where thinner section represent lower chance.

** Ten, eight and thirteen trusts were excluded from the analyses of locum intensity due to very small or zerodenominators (i.e. low permanent doctor FTE) in 2019, 2020 and 2021, respectively.

$Figure 3: Spatial map of locum intensity at the Sustainability and Transformation Partnerships (STP) level, England 2019

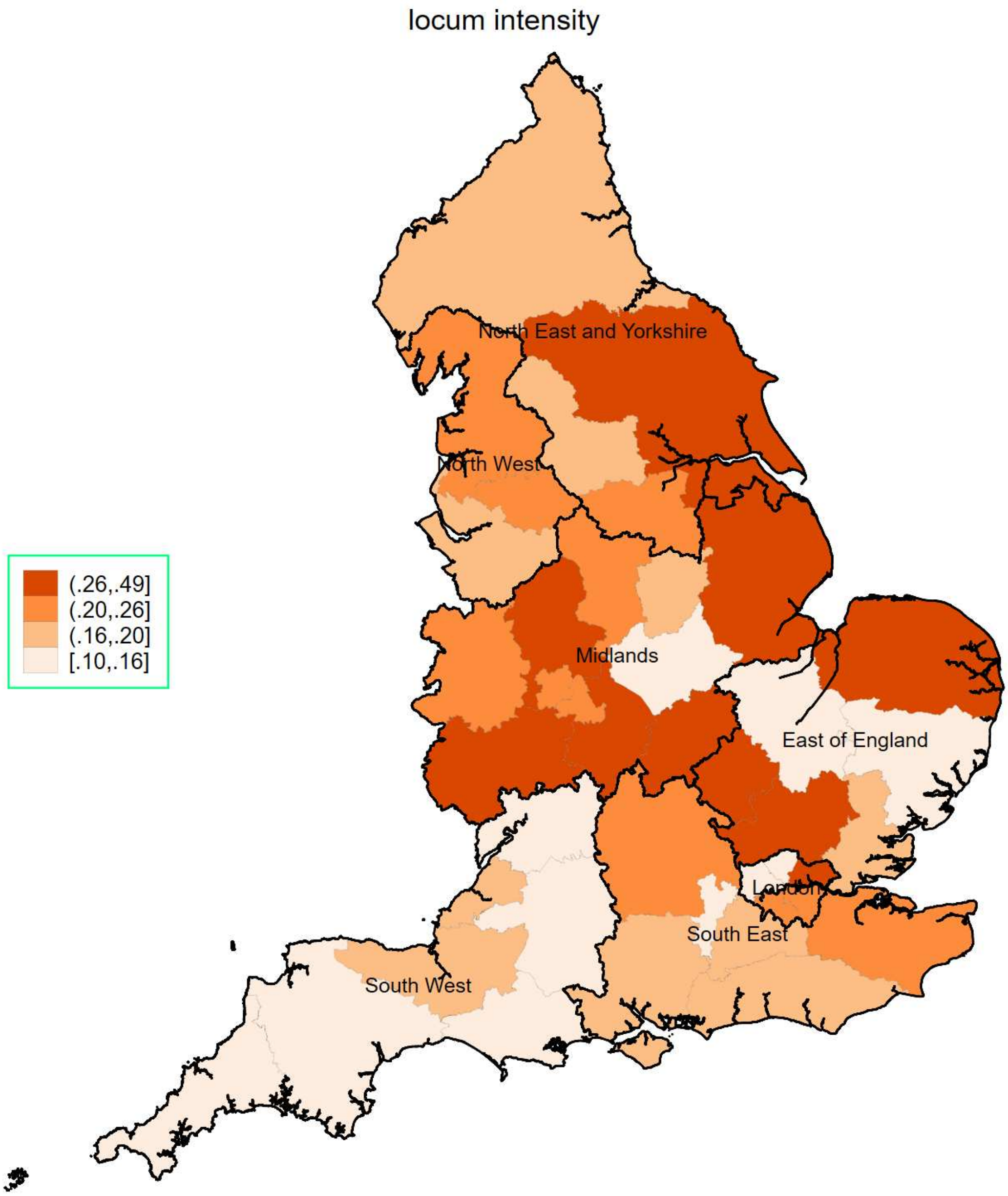

$Figure 4: Spatial map of agency shifts at the Sustainability and Transformation Partnerships (STP) level, England 2019 (%)

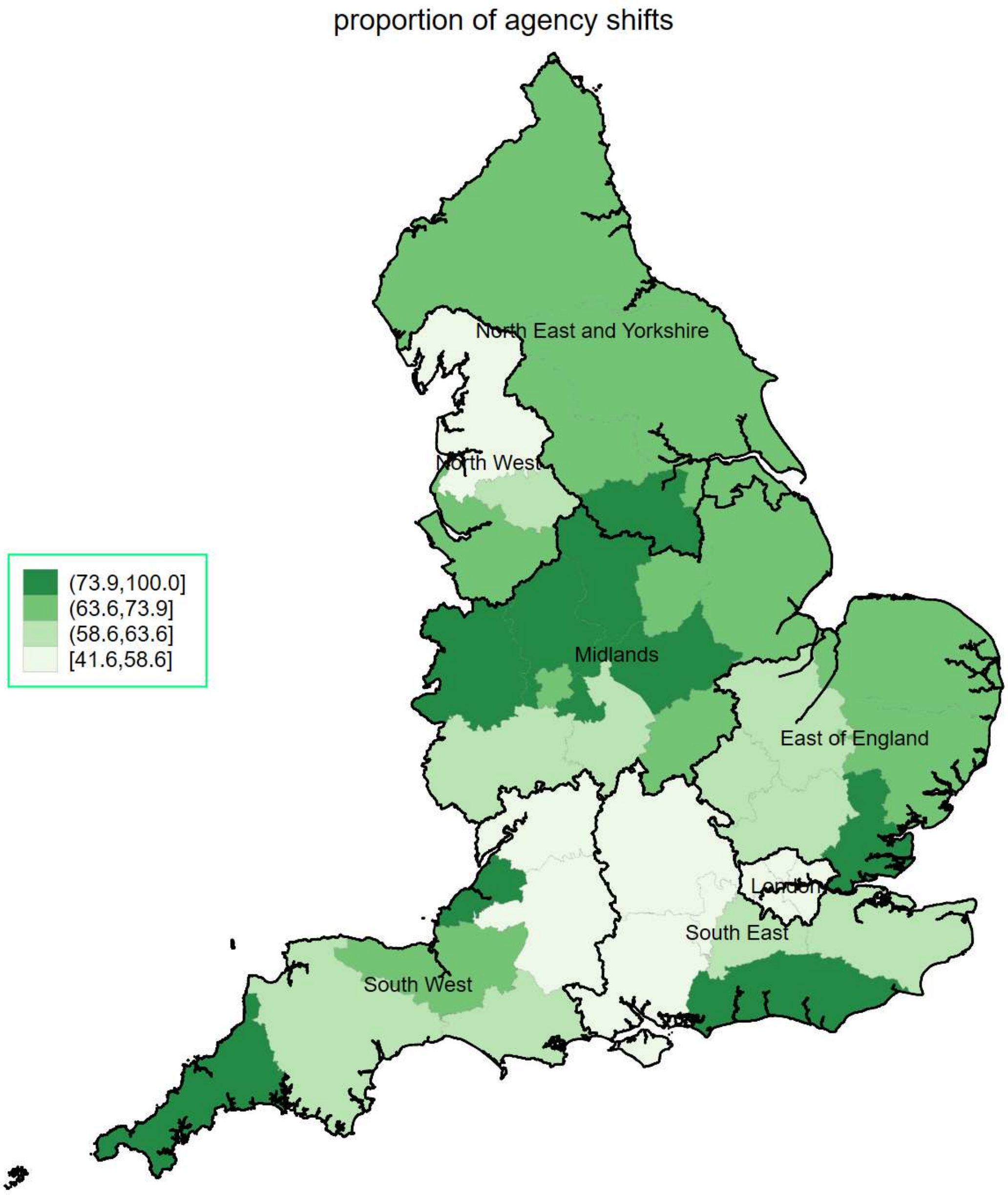

$Figure 5: Spatial map of unfilled shifts at the Sustainability and Transformation Partnerships (STP) level, England 2019 (%)

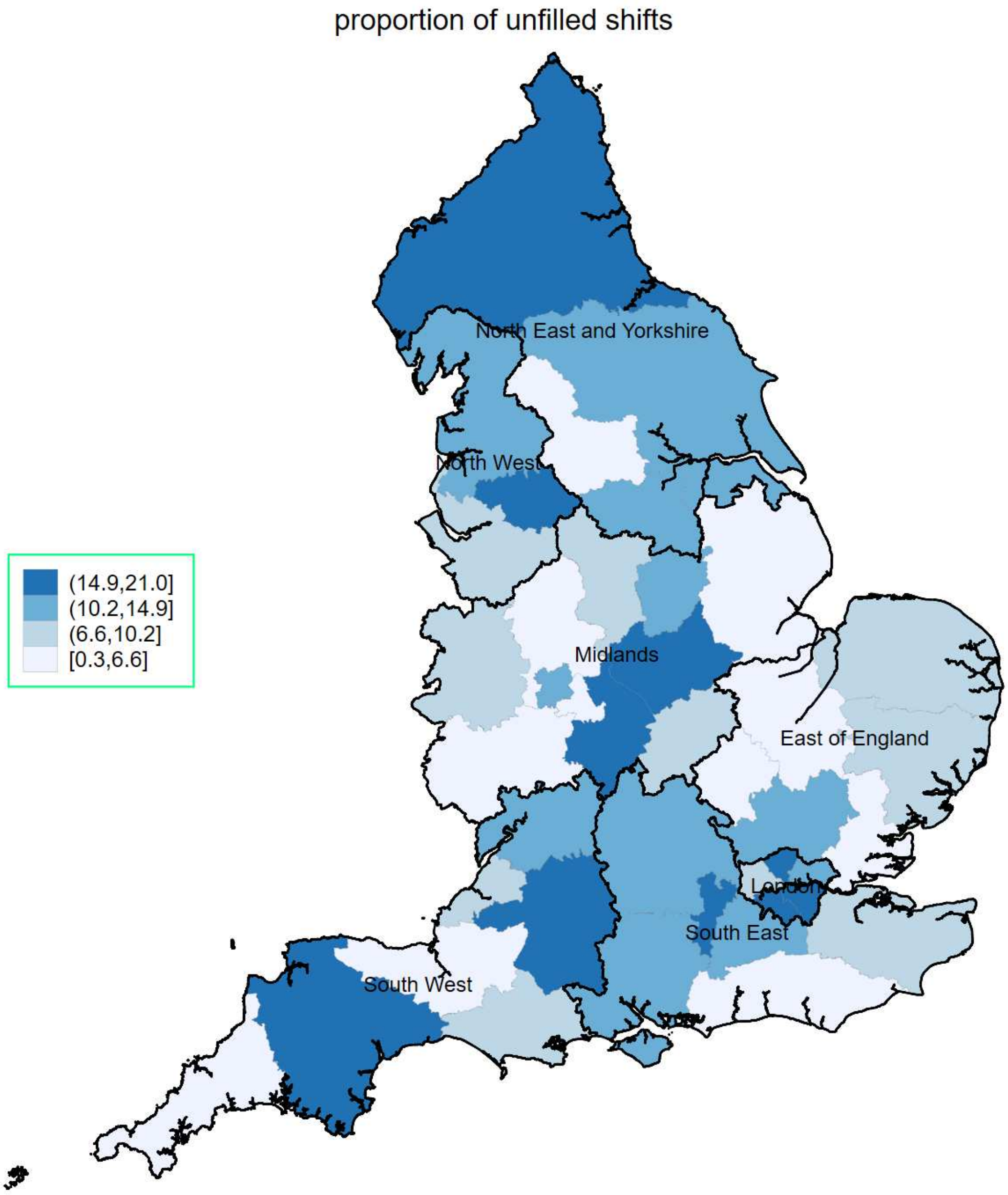

References

1. NHS Digital. NHS workforce statistics. 2022.
2. NHS Digital. NHS Vacancy Statistics. 2021.
3. Care Quality Commission. Shaping the Future: CQC's strategy 2016 to 2021. 2016.
4. Care Quality Commission. Intelligent Monitoring: NHS GP practices Indicators and methodology. 2014.
5. Care Quality Commission. Using CQC data. 2020.
6. Ministry of Housing, Communities and Local GovernmenT. The English Indices of Deprivation. Ministry of Housing, Communities and Local Government 2019.
7. NHS Digital. Hospital Episode Statistics. 2017.
8. Office for National Statistics. Open Geography Portal. 2018.
9. SHP2DTA: Stata module to convert shape boundary files to Stata datasets[program]. Boston College Department of Economics. 2006.;S456718 version.
10. Ordance Survey. A guide to coordinate systems in Great Britain. ONS, 2013:43. www.ordnancesurvey.co.uk/docs/support/guide-coordinate-systems-great-britainpdf.
