# Supplementary material for: Use of locum doctors in NHS trusts in England: analysis of routinely collected workforce data 2019–2021
Source: BMJ Open. 2023 May 15;13(6):e065803. doi: 10.1136/bmjopen-2022-065803 (PMC10254888; doi:10.1136/bmjopen-2022-065803)
Supplement: Author's manuscript [file bmjopen-2022-065803.draft_revisions.pdf]

BMJ Open

BMJ Open is committed to open peer review. As part of this commitment we make the peer review history of every article we publish publicly available.

When an article is published we post the peer reviewers' comments and the authors' responses online. We also post the versions of the paper that were used during peer review. These are the versions that the peer review comments apply to.

The versions of the paper that follow are the versions that were submitted during the peer review process. They are not the versions of record or the final published versions. They should not be cited or distributed as the published version of this manuscript.

BMJ Open is an open access journal and the full, final, typeset and author-corrected version of record of the manuscript is available on our site with no access controls, subscription charges or pay-per-view fees (http://bmjopen.bmj.com).

If you have any questions on BMJ Open's open peer review process please email info.bmjopen@bmj.com

BMJ Open

The use of locum doctors in NHS trusts in England: analysis of routinely collected workforce data 2019 – 2021.

Journal:	*BMJ Open*
Manuscript ID	bmjopen-2022-065803
Article Type:	Original research
Date Submitted by the Author:	17-Jun-2022
Complete List of Authors:	Grigoroglou, Christos; The University of Manchester, Centre for Health Economics<br>Walshe, K; The University of Manchester, Alliance Manchester Business School, Institute for Health Policy and Organisation<br>Kontopantelis, Evangelos; The University of Manchester, NIHR School for Primary Care Research, Centre for Primary Care, Division of Population Health, Health Services Research and Primary Care; The University of Manchester, Division of Informatics, Imaging and Data Sciences<br>Ferguson, Jane ; The University of Manchester, Alliance Manchester Business School, Institute for Health Policy and Organisation<br>Stringer, Gemma; The University of Manchester, Alliance Manchester Business School, Institute for Health Policy and Organisation<br>Ashcroft, Darren; The University of Manchester, NIHR School for Primary Care Research, Centre for Primary Care, Division of Population Health, Health Services Research and Primary Care; The University of Manchester, NIHR Greater Manchester Patient Safety Translational Research Centre<br>Allen, Thomas; The University of Manchester, Manchester Centre for Health Economics; University of Southern Denmark
Keywords:	Health policy < HEALTH SERVICES ADMINISTRATION & MANAGEMENT, Human resource management < HEALTH SERVICES ADMINISTRATION & MANAGEMENT, Organisation of health services < HEALTH SERVICES ADMINISTRATION & MANAGEMENT

SCHOLARONE™
Manuscripts

I, the Submitting Author has the right to grant and does grant on behalf of all authors of the Work (as defined in the below author licence), an exclusive licence and/or a non-exclusive licence for contributions from authors who are: i) UK Crown employees; ii) where BMJ has agreed a CC-BY licence shall apply, and/or iii) in accordance with the terms applicable for US Federal Government officers or employees acting as part of their official duties; on a worldwide, perpetual, irrevocable, royalty-free basis to BMJ Publishing Group Ltd ("BMJ") its licensees and where the relevant Journal is co-owned by BMJ to the co-owners of the Journal, to publish the Work in this journal and any other BMJ products and to exploit all rights, as set out in our licence.

The Submitting Author accepts and understands that any supply made under these terms is made by BMJ to the Submitting Author unless you are acting as an employee on behalf of your employer or a postgraduate student of an affiliated institution which is paying any applicable article publishing charge ("APC") for Open Access articles. Where the Submitting Author wishes to make the Work available on an Open Access basis (and intends to pay the relevant APC), the terms of reuse of such Open Access shall be governed by a Creative Commons licence – details of these licences and which Creative Commons licence will apply to this Work are set out in our licence referred to above.

Other than as permitted in any relevant BMJ Author's Self Archiving Policies, I confirm this Work has not been accepted for publication elsewhere, is not being considered for publication elsewhere and does not duplicate material already published. I confirm all authors consent to publication of this Work and authorise the granting of this licence.

For peer review only

The use of locum doctors in NHS trusts in England: analysis of routinely collected workforce data 2019 – 2021.

Christos Grigoroglou[1] (christos.grigoroglou@manchester.ac.uk)

Kieran Walshe[2] (kieran.walshe@manchester.ac.uk)

Evangelos Kontopantelis[3, 4] (e.kontopantelis@manchester.ac.uk)

Jane Ferguson[2] (jane.ferguson@manchester.ac.uk)

Gemma Stringer[2] (gemma.stringer@manchester.ac.uk)

Darren M Ashcroft[3, 5 6] (darren.ashcroft@manchester.ac.uk)

Thomas Allen[1, 7] (thomas.allen@manchester.ac.uk)

1. Manchester Centre for Health Economics, Division of Population Health, Health Services Research and Primary Care, University of Manchester, Manchester, UK,
2. Alliance Manchester Business School, Institute for Health Policy and Organisation, University of Manchester, Manchester, UK
3. NIHR School for Primary Care Research, Centre for Primary Care, Division of Population Health, Health Services Research and Primary Care, University of Manchester, Manchester, UK
4. Division of Informatics, Imaging and Data Sciences, University of Manchester, Manchester, UK
5. NIHR Greater Manchester Patient Safety Translational Research Centre, University of Manchester, Manchester, UK
6. Centre for Pharmacoepidemiology and Drug Safety, School of Health Sciences, Faculty of Biology, Medicine and Health, University of Manchester, Manchester, UK
7. Danish Centre for Health Economics, University of Southern Denmark, Denmark

Corresponding author: Christos Grigoroglou (christos.grigoroglou@manchester.ac.uk)

For peer review only - http://bmjopen.bmj.com/site/about/guidelines.xhtml

Abstract

Objectives: Locum doctors play an important role in the delivery of care in the NHS, however, little is known about the extent of locum use in NHS trusts. This study aimed to quantify and describe locum use for all NHS trusts in England in 2019–2021.

Methods: Descriptive analyses of data on locum shifts from all NHS trusts in England in 2019-2021. Weekly data were available for the number of shifts filled by agency and bank staff and the number of shifts requested by each trust. Negative binomial models were used to investigate the association between the proportion of medical staffing provided by locums and NHS trust characteristics.

Results: In 2019, on average 4.4% of total medical staffing was provided by locums, but this varied substantially across trusts (25th–75th centile=2.2% to 6.6%). Over time, two thirds of locum shifts were filled by locum agencies and a third by trusts' staff banks. On average 10% of shifts requested were left unfilled. In 2019-2021, the mean number of weekly shifts per trust increased by 17% (175.2 to 208.6) and the mean number of weekly unfilled shifts per trust increased by 52.2% (32.8 to 49.95). Trusts rated by the Care Quality Commission as inadequate or requiring improvement (IRR=1.495; 95%CI 1.191 to 1.877), and smaller trusts had a higher use of locums. Large variability was observed across regions for use of locums, proportion of shifts filled by locum agencies, and unfilled shifts.

Conclusions: There were large variations in the demand for and use of locum doctors in NHS trusts. Trusts with poor CQC ratings and smaller trusts app1ear to use locum doctors more intensively compared to other trust types. Unfilled shifts were at a three-year high at the end of 2021 suggesting increased demand which may result from growing workforce shortages in NHS trusts

Keywords: employment, locum doctors, health workforce, regional variation, NHS trusts

Word count: 3,931 words

Strengths and limitations of the study
• National study covering every NHS trust in England.
• Data on locum doctors across NHS trusts have recently become available for the first time and this research study is the first to utilise this dataset.
• Outcomes investigated included measures of locum use across trusts as well as outcomes pertaining to how trusts recruit locum doctors and how well trusts are able to fill their needs with locum doctors.
• The data lacks some important information such as the types of medical specialties covered by locum doctors which would provide an improved understanding of the work that locums do.
• Information on length of locum shifts would enable us to capture more accurately the level of locum intensity at the NHS trust level.

For peer review only - http://bmjopen.bmj.com/site/about/guidelines.xhtml

Introduction

In the UK, challenges in the recruitment and retention of medical staff in the National Health System (NHS) have resulted in significant staff shortages. (1-4) In 2018, 43% of NHS consultant posts in general medicine which were advertised were not filled and 40% of consultants and 63% of higher specialist trainees said that rota gaps occurred on a daily or weekly basis. (5), while recent surveys of middle grade doctors reported that their workload has become unsustainable under current staffing levels. (4)

When faced with medical workforce shortages, NHS trusts can fill shifts using locum doctors recruited through locum agencies, third party organisations who contract healthcare professionals to work temporarily within the NHS, or through internal staff banks. Increases in NHS expenditure for agency staff led to NHS Improvement introducing a locum pay cap to curb agency expenditure and a weekly system for gathering data on locum usage by NHS trusts in 2016. (6) The new set of rules for agency staff resulted in a reduction in spending on locum agency doctors from £3.6 billion in 2015/16 to £2.4 billion in 2018/19 (7), although many hospital trusts have applied for extensions of these price caps to fill their workforce gaps. (4) Despite national information on expenditure, little is known about the extent of locum doctors working across NHS trusts, in contrast to general practice where NHS Digital has published workforce data since 2015. (8, 9) To date, no study has described the scale of locum usage in NHS trusts in England.

The aim of this study was to use NHS Improvement data to quantify and describe locum use, and its variation, for all acute, ambulance, community and mental health NHS trusts in England from January 2019 to December 2021. We describe the rate at which NHS trusts were able to fill locum shifts and whether NHS trusts find their locum workforce via their own NHS staff banks or via locum agencies. We explore regional variations for these measures and identify NHS trusts with the highest and lowest locum usage in 2019. Finally, we examine whether some NHS trust and population characteristics explain variability in locum use at the trust level.

Methods

Data

NHS trust temporary staff employment data

In England, NHS Improvement is responsible for setting out rules which trusts are expected to follow on temporary staff expenditure. The rules have a strong focus on providing support to trusts to reduce their expenditure and to move towards a sustainable model of temporary staffing. To fulfil this responsibility and support trusts, NHS Improvement collects information

For peer review only - http://bmjopen.bmj.com/site/about/guidelines.xhtml

from all NHS trusts on their employment of temporary staff. These data are not published and were secured for research through a bespoke data-sharing agreement.

We analysed data on locum use for all NHS trusts in England between January 2019 and December 2021. Data record the weekly number of shifts that were filled by bank or agency locums for each acute, ambulance, community and mental health trust in England. A shift is defined as the period between the doctor commencing and finishing their work but the duration of shifts is not collected. Bank staff are defined as staff who are usually sourced in-house or from temporary staff banks such as NHS Professionals, which is the largest of these banks supplying temporary staff to NHS trusts. (10) Agency staff are defined as staff who are not on the payroll of the NHS organisation offering employment and are sourced from a third-party agency.

NHS Improvement data record information on the number of shifts filled by temporary staff in all staff groups but we focus on the medical and dental group which includes the aggregate number of shifts, done by all doctors and dentists. The data contain the total number of shifts that were filled by bank staff, the total number of shifts filled by agency staff and the total number of shifts requested by each trust in every reporting week, grouped for doctors and dentists. A detailed table of all the variables in the NHS Improvement data is provided in the supplementary material.

NHS trust characteristics

We collected monthly data on all trusts' substantive employees represented as full-time equivalents (FTE) and trust annual job turnover data for the medical and dental staff group using the NHS Workforce Statistics database. (11) Trust type information and trust overall inspection ratings were obtained from the Care Quality Commission (CQC) which rates NHS trusts as outstanding, good, requiring improvement or inadequate. (12) Trust level deprivation was derived using hospital admissions data from NHS Digital and aggregating inpatient postcode deprivation for each trust. (13) Trust level vacancy rates were obtained in the form of advertised FTE roles for medical and dental staff, available from the NHS Vacancy Statistics from NHS Digital. (14) These trust characteristics were linked to temporary staffing data using unique trust identifiers and are discussed in detail in the supplementary material.

Analyses

Outcome measures

Locum intensity

For peer review only - http://bmjopen.bmj.com/site/about/guidelines.xhtml

Our primary outcome measured locum intensity for each NHS trust in every reporting week. To calculate locum intensity, we combined bank and agency shifts to obtain the total number of shifts reported at trust level in every reporting week. We adjusted this weekly total by the size of the permanent medical and dental workforce in each trust, specifically, the total number of locum shifts was divided by permanent doctor FTE, including dentists, (i.e. FTE of NHS and Community Health Hospital Doctors, Consultants, Associate Specialists, Specialty Doctors, Specialty Registrars, Foundation Doctors/Postgraduate Doctors) to give the locum intensity. The annual mean locum intensity was calculated over 12 months of data. A locum intensity of 0.25 indicates that the trust filled 0.25 locum shifts per week per FTE permanent doctor. We report locum intensity in this way because we do not know the length of the reported locum shifts and therefore cannot directly convert them into FTE. If we assume that one FTE permanent doctor typically works five shifts per week and that shift length for permanent doctors and locum doctors is broadly equivalent, then a locum intensity of 0.25 means that 5% of medical staffing in that week was provided by locums.

Proportion of agency shifts

Our second outcome measured trusts' reliance on agency staff, which are more costly than bank staff. We divided the number of agency shifts by the total number of filled shifts for every trust in every reporting week. An annual mean proportion of agency shifts was then calculated for each trust over 12 months of data.

Proportion of unfilled shifts

Our third outcome measures shifts that the trust was unable to fill. The total number of shifts requested by each trust in every week was provided by NHS Improvement. The number of filled shifts was subtracted from the number of shifts requested to obtain the number of unfilled shifts for each trust in each week. We calculated the proportion of unfilled shifts by dividing unfilled shifts by shifts requested. An annual mean proportion of unfilled shifts was calculated for each trust over 12 months of data. Trusts occasionally reported a higher number of shifts filled than requested. In these cases, we adjusted the number of shifts requested to reflect the number of total shifts filled in that week. These adjustments were made 811 times out of 11,450 (7.1%) trust-week observations in 2019.

A worked example of the algorithm that we used in each calculation is illustrated in Box 1. Our analysis dataset contained information on locum intensity, proportion of agency shifts, proportion of unfilled shifts and trust characteristics for 229 acute, mental health, ambulance and community health trusts in 2019. Of these, three acute trusts and one ambulance trust did not report data on monthly doctor FTE, and one acute trust and seven ambulance trusts reported zero weekly locum returns in every reporting week. Eight ambulance, one acute,

For peer review only - http://bmjopen.bmj.com/site/about/guidelines.xhtml

one mental health and one community trust reported zero agency shifts in every reporting week. We also explored variation in the three outcomes over time, with 224 and 221 trusts reporting bank and agency shift data to NHS Improvement, in 2020 and 2021 respectively.

Our first set of analyses was descriptive and we used ordered bar charts to show the distribution of locum intensity, proportion of agency shifts and proportion of unfilled shifts for all trusts in 2019 - 2021. Violin plots showed the geographic variation in each outcome across regions. We used spatial maps to illustrate the distribution of each outcome across all Sustainability and Transformation Partnerships (STPs), local partnerships aiming to improve health and quality of care in the areas they serve. Analysis from 2019 to 2021 uncovered whether trusts reported changes over time in locum intensity, proportion of agency shifts and proportion of unfilled shifts, a period including a majority of the COVID 19 pandemic in England.

Our second set of analyses was inferential and employed three mean-dispersion negative binomial regressions to model locum intensity, proportion of agency shifts, and proportion of unfilled shifts in 2019. Each model used robust standard errors with fixed-effects predictors for region (as categorical, to account for between region variation). Our dependent variables were: the mean number of total shifts (offset: natural logarithm of annual mean total permanent doctor FTE); the mean number of agency shifts (offset: natural logarithm of the annual mean total shifts); and the mean number of unfilled shifts (offset: natural logarithm of annual mean total shifts requested). Our choice of negative binomial models over standard Poisson models was based on the presence of over-dispersion in the three outcomes. We controlled for CQC inspection rating, trust type (NHS general acute trusts, NHS specialist trusts, mental health trusts, and ambulance trusts), trust size (quintiles of trust permanent doctor FTE), turnover and vacancy rates and regional effects.

The final dataset consisted of 197 trusts out of 229 trusts in 2019 with complete data for all covariates (8.6% of missing data). We performed a sensitivity regression analysis excluding 25 ambulance and community trusts as these trusts tend to employ very small numbers of doctors relative to acute and mental health NHS trusts. The exclusion of ambulance and community trusts allowed us to examine the effects of deprivation, which could only be measured for acute and mental health NHS trusts. Stata v16.1 was used for the principal data cleaning, management and analyses. We used the *nbreg* command with the exposure option.

Results

Overall locum use

For peer review only - http://bmjopen.bmj.com/site/about/guidelines.xhtml

In 2019, total unadjusted locum use for all trusts in England was 2,004,485 shifts, of which 909,029 (45.3%) were bank shifts and 1,095,455 (54.7%) were agency shifts. Trusts requested 2,316,302 shifts with a trust mean of 208 per week (SD=258.3). The completeness of the data was good with 99% of all trusts reporting at least some locum use in any week.

Locum intensity

Figure 1a plots the ranked mean locum intensity in 2019 for 219 NHS trusts in England showing significant variation in locum use across trusts. Mean locum intensity was 0.22 (SD=0.16) (median=0.195; 25th–75th centile=0.11 to 0.31), indicating 0.22 locum shifts per permanent doctor FTE. Assuming five shifts per FTE, the locum intensity is equivalent to 4.4% of medical staffing provided by locums (25th–75th centile=2.2% to 6%). Four ambulance trusts, three acute trusts and one community trust were not included in this analysis as they reported very low or zero permanent doctor FTE and therefore adjustments in their locum intensity could not be performed. The ranked rates of locum intensity in 2020 and 2021 are presented in supplementary figure1a and supplementary figure2a. We report the ten trusts with the highest and lowest reported locum intensity in 2019 in the supplementary material (Supplementary Table 1).

Proportion of agency shifts

The proportion of locum shifts filled by locum agency staff (rather than from staff banks) ranked from low to high at the trust level is depicted in Figure 1b. The use of agency shifts varied substantially across trusts in 2019 with a mean of 66.1% (SD=28.5%; median=68.9%; 25th–75th centile=43.5% to 95.8%). Half of trusts (109) reported 100% of shifts filled by agency staff at some point in 2019, of which 24 trusts reported that shifts were filled entirely by agency staff in every week. Eight ambulance, one acute, one mental health and one community trusts reported zero agency shifts in every reporting week in 2019. We present the ranked proportion of agency shifts for 2020 and 2021 in supplementary figure1b and supplementary figure2b.

Proportion of unfilled shifts

In figure 1c, trusts are ranked from low to high on their proportion of unfilled shifts. Overall, trusts were able to fill the majority of their requested shifts either via bank or agency staff but we observed substantial variation. The mean proportion of unfilled shifts was 11.3% (SD=11.9%; Median=7.23%; 25th–75th centile =0 to 18.1%). Seven ambulance and one acute trust did not request any shifts at any point in 2019. The ranked proportions of unfilled shifts for 2020 and 2021 are presented in supplementary figure1c and supplementary figure2c.

For peer review only - http://bmjopen.bmj.com/site/about/guidelines.xhtml

Regional variation in locum use

In table 1, we present descriptive statistics on outcomes and trust characteristics at the regional level for 2019. Figures 2a, 2b and 2c show regional variation in outcomes at the trust level in 2019. At the regional level, median locum intensity varied substantially from 0.13 (25th–75th centile: 0.08 to 0.2) in the South West of England to 0.26 (25th–75th centile: 0.15 to 0.35) in the Midlands (Table 1 and figure 2a). We also observed large variation in the proportion of agency shifts across regions. Trusts in London filled the lowest proportion of shifts using agency staff with a median of 44.8%, (25th–75th centile=26.6% to 87.5%) while trusts in the East of England filled the highest with a median of 78.1% (25th–75th centile=37% to 98.1) (Table 1 and figure 2b). Trusts in the East of England filled requested shifts more successfully with unfilled shifts of 3.25% (25th–75th centile: 0% to 13.1%) whereas trusts in London had unfilled shifts of 11.6% (25th–75th centile: 0.71% to 22.8%) (Table 1 and figure 2c). Regional variation for the three outcomes in 2020 and 2021 is presented in supplementary figure3 and supplementary figure4.

Table 1 - Descriptive statistics in 2019, by region*†

	East of England	London	Midlands	North East & Yorkshire	North West	South East	South West
Locum intensity *, Median (25th–75th centile)	**0.19 (0.13 to 0.30)**	**0.18 (0.08 to 0.28)**	**0.26 (0.15 to 0.35)**	**0.17 (0.08 to 0.35)**	**0.21 (0.12 to 0.31)**	**0.19 (0.11 to 0.27)**	**0.13 (0.08 to 0.2)**
Proportion of agency shifts (%) Median (25th–75th centile)	**78.1 (37 to 98.1)**	**44.8 (26.6 to 87.5)**	**75.6 (54.9 to 94.2)**	**74.7 (51 to 99.5)**	**65.3 (45.2 to 90.8)**	**60 (34.3 to 88.9)**	**77.9 (33.4 to 100)**
Proportion of unfilled shifts † (% of requested shifts), Median (25th–75th centile)	**3.25 (0 to 13.1)**	**11.6 (0.71 to 22.8)**	**3.5 (0 to 16)**	**3.9 (0 to 23)**	**6.5 (0 to 21.6)**	**4.8 (0 to 19.5)**	**5 (0 to 17.6)**
Full-time doctor FTE,	803.3 (385.4 to 1,176.5)	869.1 (398.9 to 2,061.5)	569 (220 to 1,198)	715.5 (268 to 1,246.1)	612.6 (321.9 to 1,016.4)	1,013 (298 to 1,317.7)	701.1 (229 to 1,082.2)

For peer review only - http://bmjopen.bmj.com/site/about/guidelines.xhtml

Median (25th–75th centile)							
Trust types							
NHS general acute trusts (n)	16	18	20	22	20	17	17
Acute - NHS specialist trusts (n)	1	5	3	1	6	1	-
Mental health trusts (n)	4	10	12	9	6	5	6
Community health (n)	3	2	4	1	2	5	-
Ambulance service (n)	1	1	2	2	1	2	1

* Locum intensity is adjusted for mean total full-time doctor FTE in 2019
† The proportion of unfilled shifts for trusts that reported a higher number of shifts filled than shifts requested was capped at 100%.

We investigated spatial variation within and between regions using spatial maps at the Sustainability and Transformation Partnership level (see supplementary figures 5, 6 and 7). Substantial variability was observed for all three outcome both within and between regions. High levels of locum intensity were concentrated in the Midlands, the North East & Yorkshire, and the North West. The South East and South West ranked among the lowest in terms of locum intensity. High proportions of agency shifts were observed in areas in the Midlands, the East of England, and the North East & Yorkshire. London had by far the lowest proportion of agency shifts. The proportion of unfilled shifts was high in areas in London, the Midlands and the South West and low in the East of England.

Results from regression analyses

The regression analyses results using the three different outcomes are presented in table 2. The results are reported as incidence rate ratios (IRRs) for the coefficients of interest. Sensitivity analyses where we excluded ambulance and community trusts and examined the effects of deprivation on our three outcomes were nearly identical to the results from the

For peer review only - http://bmjopen.bmj.com/site/about/guidelines.xhtml

main analyses. Deprivation did not appear to have any discernible effect on any of the three outcomes. The results from the sensitivity analyses are provided in supplementary table2.

Table 2: Negative binomial regression analyses for the three outcomes in 2019, IRR [a,b,c]

	**Locum Intensity**	**Agency shifts**	**Unfilled shifts**
**Trust level aggregate FTE (reference group is quintile 1)**	**Reference group**	**Reference group**	**Reference group**
Quintile 2	0.784 (0.527 to 1.676), <0.231 (0.159)	0.945 (0.734 to 1.218), <0.662 (0.122)	0.936 (0.449 to 1.952), <0.859 (0.351)
Quintile 3	0.496 (0.299 to 0.825), <0.007 (0.129)	0.937 (0.675 to 1.301), <0.698 (0.157)	1.848 (0.735 to 4.645), <0.192 (0.869)
Quintile 4	0.611 (0.349 to 1.072), <0.086 (0.175)	0.883 (0.617 to 1.264), <0.497 (0.162)	1.878 (0.704 to 5.011), <0.208 (0.940)
Quintile 5	0.347 (0.187 to 0.644), <0.001 (0.110)	0.796 (0.530 to 1.195), <0.271 (0.165)	2.447 (0.826 to 7.251), <0.106 (1.356)
**Trust type (reference group is NHS general acute trust)**	**Reference group**	**Reference group**	**Reference group**
NHS specialist trust	0.285 (0.174 to 0.468), <0.001 (0.072)	1.510 (1.086 to 2.100), <0.014 (0.254)	0.233 (0.091 to 0.598), <0.002 (0.112)
Mental health trust	0.966 (0.628 to 1.486), <0.875 (0.212)	1.576 (1.198 to 2.073), <0.001 (0.220)	1.062 (0.508 to 2.221), <0.873 (0.400)
Ambulance service	55.43 (20.56 to 149), <0.001 (27.96)	0.033 (0.008 to 0.147), <0.001 (0.025)	3.894 (0.453 to 33.14), <0.215 (4.272)
Community service	1.443 (0.780 to 2.670), <0.243 (0.453)	0.962 (0.641 to 1.445), <0.854 (0.199)	1.360 (0.471 to 3.930), <0.570 (0.736)
**CQC ratings (reference group is trusts that provide good and outstanding services)**	**Reference group**	**Reference group**	**Reference group**

For peer review only

For peer review only - http://bmjopen.bmj.com/site/about/guidelines.xhtml

Inadequate and requiring improvement	1.495 (1.191 to 1.877), <0.001 (0.173)	1.044 (0.907 to 1.201), <0.550 (0.075)	1.193 (0.789 to 1.804), <0.402 (0.251)
**Trust level substantive doctor turnover rates**	1.015 (1.009 to 1.021), <0.001 (0.002)	1.001 (0.997 to 1.003), <0.589 (0.001)	0.995 (0.987 to 1.003), <0.248 (0.004)
**Trust level vacancy rates [full-time equivalent (FTE)]**	1.000 (0.999 to 1.001), <0.530 (0.005)	0.999 (0.999 to 1.001), <0.948 (0.001)	0.999 (0.997 to 1.001), <0.585 (0.001)
**Region (reference region is London)**	**Reference group**	**Reference group**	**Reference group**
South West	0.575 (0.361 to 0.915), <0.019 (0.136)	1.447 (1.098 to 1.907), <0.009 (0.204)	0.687 (0.316 to 1.493), <0.343 (0.272)
South East	0.701 (0.472 to 1.041), <0.078 (0.141)	1.349 (1.047 to 1.736), <0.021 (0.175)	0.524 (0.252 to 1.092), <0.085 (0.196)
Midlands	1.041 (0.714 to 1.520), <0.832 (0.201)	1.425 (1.126 to 1.804), <0.003 (0.172)	0.548 (0.276 to 1.086), <0.085 (0.191)
East of England	0.813 (0.533 to 1.240), <0.336 (0.175)	1.525 (1.167 to 1.993), <0.002 (0.208)	0.402 (0.182 to 0.890), <0.025 (0.163)
North West	1.045 (0.705 to 1.550), <0.826 (0.210)	1.327 (1.035 to 1.701), <0.026 (0.168)	0.855 (0.412 to 1.773), <0.673 (0.318)
North East and Yorkshire	0.754 (0.495 to 1.150), <0.191 (0.162)	1.449 (1.120 to 1.875), <0.005 (0.191)	0.575 (0.269 to 1.230), <0.154 (0.223)
constant	0.030 (0.152 to 0.601), <0.001 (0.105)	0.436 (0.283 to 0.671), <0.001 (0.096)	0.117 (0.038 to 0.357), <0.001 (0.066)

[a] Model A included data on 220 trusts (observation) while models B and C included data on 214 trusts with robust standard errors.
[b] 95% confidence intervals are in brackets; results are reported as incidence rate ratios (IRR) followed by *P*-values and standard errors in parentheses.
[c] Coefficients can be interpreted as proportionate changes, for example, trusts in the North West had on average 4.5% lower locum intensity than trusts in London.

Locum intensity

Results indicate that in 2019 trust size was a strong predictor of locum intensity. Using quintile 1 (i.e. small trust size) as the reference group, our results showed significant reductions in locum intensity for medium and very large trusts with IRRs of 0.496 (95% CI 0.299 to 0.825) for quintile 3, and 0.347 (95% CI 0.187 to 0.644) for quintile 5. As an example of interpretation, comparing quintile 1 and quintile 3 suggests a locum intensity 50.4% lower for the medium size trusts. NHS specialist trusts had a 71.5% lower locum intensity (IRR=0.285; 95% CI 0.174

For peer review only - http://bmjopen.bmj.com/site/about/guidelines.xhtml

to 0.468) than NHS general acute trusts. Ambulance service trusts had 55 times higher locum intensity than NHS general acute (IRR=55.43; 95% CI 20.56 to 149). However, this result is an artefact of the very low numbers of permanent doctors employed by ambulance trusts when compared to other trusts. CQC ratings were strongly associated with higher locum intensity with trusts rated as inadequate or required improvement having 49.5% (IRR=1.495; 95% CI 1.191 to 1.877) higher mean locum intensity than trusts rated good or outstanding. Staff turnover rates had negligible effects on locum intensity (IRR=1.015; 95% CI 1.009 to 1.021). Trusts in the South West had 40.25% lower locum intensity than trusts in London (IRR=0.575; 95% CI 0.361 to 0.915).

Proportion of agency shifts

NHS specialist trusts and mental health trusts had 51% (IRR=1.510; 95% CI 1.086 to 2.100) and 57.6% (IRR=1.576; 95% CI 1.198 to 2.07) higher proportion of agency shifts than NHS general acute trusts. Ambulance service trusts had 96.7% lower proportion of agency shifts (IRR=0.033; 95% CI 0.008 to 0.147) than NHS general acute trusts. Trusts in the East of England had the highest proportion of agency shifts compared to trusts in London (IRR=1.525; 95% CI 1.167 to 1.993).

Proportion of unfilled shifts

NHS specialist trusts had 76.7% higher proportion of unfilled shifts when compared to NHS general acute trusts. Trusts in the East of England had 59.80 lower rates of unfilled shifts when compared to trusts in London (IRR=0.402; 95% CI 0.182 to 0.890).

Locum use during the COVID-19 pandemic

Figure 3 shows the mean agency, bank, unfilled and total shifts per week at the trust level in 2019 to 2021. Over time, the trust level mean was 188.5 shifts per week (SD=205.8), of which 95.2 (SD=108.6) were agency shifts and 93.3 (SD=135.8) were bank staff shifts and the mean of unfilled shifts across all trusts was 38.5 (SD =85.2). Pre-pandemic, we observed small variability in the mean number of agency, bank and unfilled shifts. In March 2020 there was a steep decline (approximately 18%) in agency and bank shifts per trust as very few trusts reported locum use between March and April. In the third quarter of 2020, we observed an increase (approximately 15%) in agency and bank shifts per trust. In 2021, there was a steep steady increase in the mean number of unfilled shifts from 33.9 to 50.1 (47.8% increase) between May and June, which was sustained throughout 2021 and reached a peak of 69.2 unfilled shifts per trust in December 2021.

For peer review only - http://bmjopen.bmj.com/site/about/guidelines.xhtml

Discussion

Summary

This study provides evidence on the extent of locum use and factors associated with locum use in NHS trusts in England for the period 2019 – 2021. Our findings show that on average 4.4% of medical staffing in NHS trusts in 2019 was provided by locum medical staffing. Trusts with lower CQC ratings, acute trusts and smaller trusts had higher locum intensity. We observed moderate variability in locum use across regions and greater variability in the proportion of shifts filled by agency locums. During 2021, the proportion of shifts that were unfilled reached a three year high. Our findings can help inform NHS organisations about the extent of their locum use and can provide important information about the effective planning of the NHS workforce.

Strengths and limitations of the study

The main strength of this study is the national scope and coverage of every NHS trust of England. For the first time, using routinely collected data on locum use, we quantified the extent of locum use, sourced from agencies or banks, across all NHS trusts for the period 2019-2021. We also explored whether trusts were able to cover sufficiently for staff shortages and identified drivers of locum use at the trust level for the whole of England. We reveal the impact of COVID-19 on locum use in NHS Trust. Our analyses allowed us to control for measured trust and population characteristics.

However, this study has some important limitations which should be considered when interpreting the key findings. First, the NHS Improvement data do not reveal information on locum use by specialty and there may be substantial variations across specialties which we could not identify. Second, although NHS Improvement collects data on the number of locum shifts, it does not collect the shift duration or locum FTE which would allow a more straightforward comparison with permanent doctor FTE. We had to assume that shift lengths for permanent and locum doctors were broadly equivalent in order to estimate the proportion of medical staffing provided by locum doctors. Third, there may also exist variability in locum use between locums of different types (e.g. infrequent or long-term locums) or durations apart from the agency/bank categories, which has been observed in general practice. (9) Some locums may be employed for several months (15) often to cover a vacancy which has not been filled, while others may cover short-term absences such as illness for as little as one or two shifts and we did not have that information. Fourth, the dataset has no information on how well NHS trusts use their locum workforce such as the provision of adequate induction, training, supervision and feedback in accordance with NHSE guidance.

For peer review only - http://bmjopen.bmj.com/site/about/guidelines.xhtml

Prior work (16) suggests that locum performance is driven more by organisational attributes such as these than by the characteristics of the locum doctors themselves.

Interpretation of findings

The use of locum doctors is important because of the high level of spending it entails and because of concerns about the quality and safety of locum staffing arrangement. (17) Our study shows that the actual level of locum use, as a proportion of overall medical staffing, is relatively low on average, but varies considerably, with some trusts having much higher use of locums and some trusts relying overly on more expensive agency locums rather than using staff banks.

Some of this variation may be explained by organisational characteristics. For instance, larger trusts may be more able to cover workforce gaps within their own staff without needing locums, and specialist/tertiary trusts may find it easier to recruit and provide attractive workplaces compared with general acute trusts. Mental health trusts may face particular staffing shortages, which may explain the high level of agency locum use.

Our results show significantly higher locum intensity in trusts with worse CQC ratings (inadequate or requires improvement). It may be that these trusts find it harder to recruit and fill workforce gaps, but it could also be hypothesised that sustained high levels of locum use may impact quality and safety and hence affect CQC ratings.

The introduction of the first UK lockdown brought significant reductions in the numbers of both bank and agency locum doctors employed across NHS trusts, due to cancelations in elective care. However, shortly after, trusts started employing more locums likely in an effort to tackle excessive workload and increasing demand for health care services during the pandemic. Furthermore, in 2021, we observed an increase in the mean number of shifts filled by bank compared to the previous years and this was accompanied with a stable trend in agency shifts and an increase in the number of unfilled shifts. This suggests that trusts were meeting the increased demand with bank staff, which is in line with the new agency rules enacted by NHS Improvement in 2019 that aim to reduce reliance on agency staff. (18) Despite the increase in the mean number of total shifts, trusts appeared to be less able to fill the number of shifts they were requesting over the second half of 2021. This may suggest a persisting high workload for permanent doctors that trusts were unable to address with the use of locum doctors over that period.

For peer review only - http://bmjopen.bmj.com/site/about/guidelines.xhtml

Contributors: CG, TA and KW designed the study. CG extracted the data from all sources, performed the analyses and drafted the manuscript. KW, EK, JF, GS, DA and TA critically revised the manuscript. CG is the guarantor of this work and, as such, had full access to all the data in the study and takes responsibility for the integrity of the data and the accuracy of the data analysis.

Data availability statement: Data may be obtained from a third party and are not publicly available. Data can be obtained from NHS Improvement under a special license and are not freely available.

Patient consent for publication: Not required.

Patient and Public Involvement: None.

Acknowledgments: We would like to thank NHS Improvement and NHS Digital for the wealth of information they have collected and systematically organised, which made this study possible.

Competing interests: The authors have declared no competing interests.

Ethics Approval: No ethics approval was required as the study uses aggregated data.

Funding: This study was funded by the National Institute for Health Research (NIHR) Health Service and Delivery Research programme (project reference number: NIHR128349). The views expressed are those of the authors and not necessarily those of the NIHR or the Department of Health and Social Care.

For peer review only - http://bmjopen.bmj.com/site/about/guidelines.xhtml

Worked example of outcome measure calculations for Manchester University NHS Foundation trust in 2019.

To obtain the mean locum intensity for Manchester University NHS Foundation trust in 2019, we combined the number of bank and agency shifts to calculate the total number of filled shifts out of the number of shifts requested. For every reporting week in 2019, we divided the total number of shifts by the monthly permanent doctor FTE reported for the in that month. For example, in the week commencing 7th January 2019, Manchester University NHS Foundation trust reported 205 agency shifts and 283 bank staff shifts. We divided the total number of shifts (i.e. 488) by the reported permanent doctor FTE in January (i.e. 4,378.8) to obtain a locum intensity value of 0.11, suggesting that for every one full-time doctor the trust had 0.11 locum doctor shifts that week. That would equate to 2.2% [(0.11/5)*100] of care provided by locums in that week if we assume five shifts per FTE.

We calculated the proportion of shifts filled by agency staff by dividing the total number of agency shifts by the total number of all shifts (agency and bank) for each trust in every reporting week. For example, the proportion of agency shifts for Manchester University NHS Foundation trust in the week commencing 7th January 2019 was (205/488)*100=42%.

We also had information on the number of shifts that each trust requested in every reporting week. For the same week, Manchester University NHS Foundation trust requested 574 bank and agency shifts but failed to fill 86 of these giving an unfilled rate of 15% [(86/574)*100].

For peer review only - http://bmjopen.bmj.com/site/about/guidelines.xhtml

References

1. Rimmer A. NHS England workforce plan fails to tackle staff shortages, say health leaders. BMJ. 2020;370:m3057; 2020.
2. Kings Fund. NHS workforce: our position. London King's Fund. 2021.
3. Buchan J, Charlesworth A, Gershlick B, Seccombe I. A critical moment: NHS staffing trends, retention and attrition. The Health Foundation. 2019.
4. Anderson M, O'Neill C, Macleod Clark J, Street A, Woods M, Johnston-Webber C, et al. Securing a sustainable and fit-for-purpose UK health and care workforce. Lancet. 2021;397(10288):1992-2011.
5. Focus on physicians; census of consultant physicians and higher specialty trainees 2018-19. London. Royal College of Physicians. 2019.
6. NHS Improvement. Reducing reliance on agency staff: consultation response. 2019.
7. Department of Health. The Department of Health and Social Care's written evidence to the Review Body on Doctors' and Dentists' Remuneration (DDRB) for the 2020/21 Pay Round. Department of Health. 2020.
8. NHS Digital. General Practice Workforce. 2019.
9. Grigoroglou C, Walshe K, Kontopantelis E, Ferguson J, Stringer G, Ashcroft DM, et al. Locum doctor use in English general practice: analysis of routinely collected workforce data 2017–2020. Br J Gen Prac. 2022;72(715):e108-e17.
10. NHS Professionals. About NHS Professionals. 2022.
11. NHS Digital. NHS workforce statistics. 2019.
12. Care Quality Commission. Using CQC data. 2020.
13. NHS Digital. Hospital Episode Statistics. 2017.
14. NHS Digital. NHS Vacancy Statistics. 2021.
15. The British Medical Association. Locum working for SAS doctors. London. 2022.
16. Ferguson J, Walshe K. The quality and safety of locum doctors: a narrative review. J R Soc Med. 2019;112(11):462-71.
17. Ferguson J, Tazzyman A, Walshe K, Bryce M, Boyd A, Archer J, et al. 'You're just a locum': professional identity and temporary workers in the medical profession. Sociol Health Illn. 2021;43(1):149-66.
18. NHS Improvement. Agency rules. 2019

For peer review only

For peer review only - http://bmjopen.bmj.com/site/about/guidelines.xhtml

Fig 1: Locum intensity, proportion of agency shifts and proportion of unfilled shifts in 2019, NHS trust level

a)Mean locum intensity (bank and agency shifts combined) at trust level in 2019†*

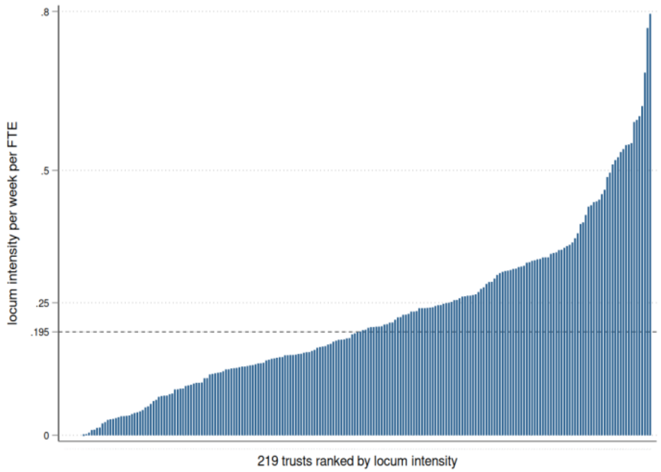

† red line indicates the median (0.195) locum intensity per week per FTE across 219 NHS trusts in 2019

* 10 trusts were excluded from the analyses due to very small or zero denominators (i.e. low permanent doctor FTE)

b)Mean proportion of agency shifts at trust level in 2019††

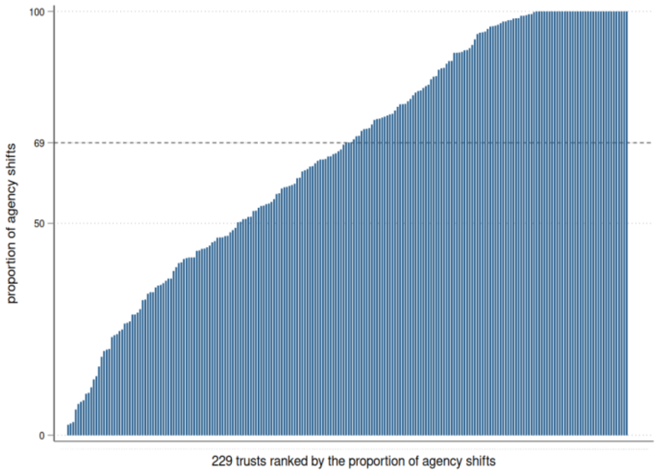

†† red line indicates the median (69%) proportion of agency shifts per week across 229 NHS trusts in 2019

c)Mean proportion of unfilled shifts at trust level in 2019¥

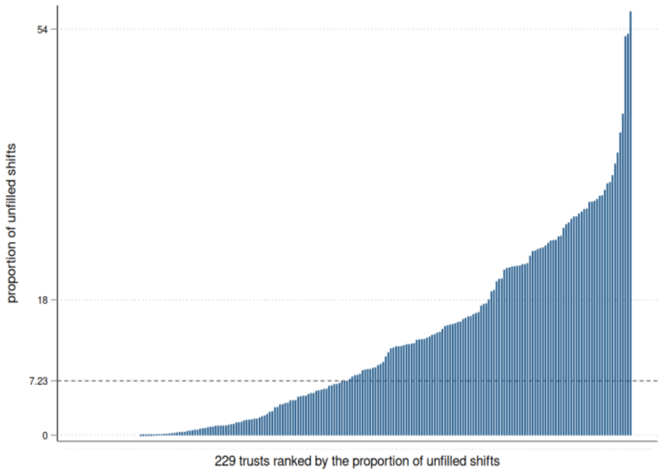

¥ red line indicates the median (7.23%) unfilled shift rate per week across 229 NHS trusts in 2019.

For peer review only - http://bmjopen.bmj.com/site/about/guidelines.xhtml

Fig 2: Regional variation in locum intensity, proportion of agency shifts and proportion of unfilled shifts, 2019*

a) Regional variation in locum intensity (bank + agency shifts combined) in 2019†**

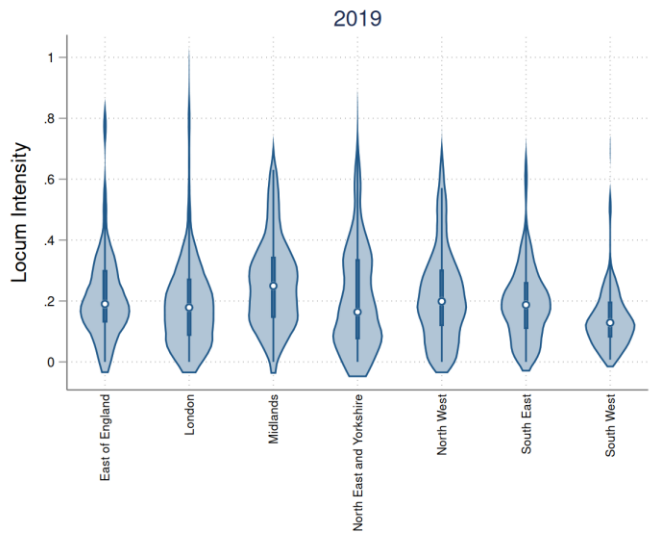

† Figure includes data from 222 NHS trusts in 2019, adjusted for permanent doctor FTE.

b) Regional variation in the proportion of agency shifts in 2019††

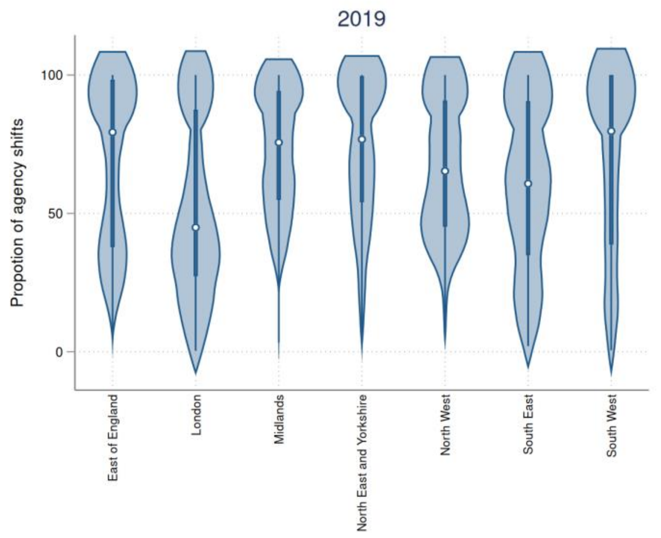

†† Figure includes data from 229 NHS trusts in 2019

c) Regional variation in the proportion of unfilled shifts in 2019¥

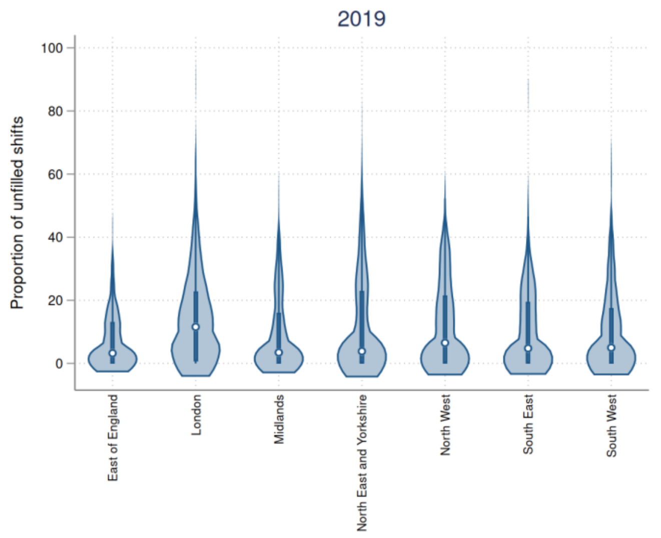

¥ Figure includes data from 229 NHS trusts in 2019

* The thick blue line represents the interquartile range (25th–75th centile) and the thin line represents the rest of the distribution with upper/lower adjacent values. The white dot represents the median of the data. The distribution shape of the data is based on a kernel density estimation where wider sections of the plot represent a higher chance that members of the population of interest will take on a given value and where thinner section represent lower chance.

**Seven trusts were excluded from the analyses due to non-availability of data for permanent doctor FTE.

For peer review only - http://bmjopen.bmj.com/site/about/guidelines.xhtml

Fig 3: Variation in mean number of locum shifts over time, 2019 – 2021*±

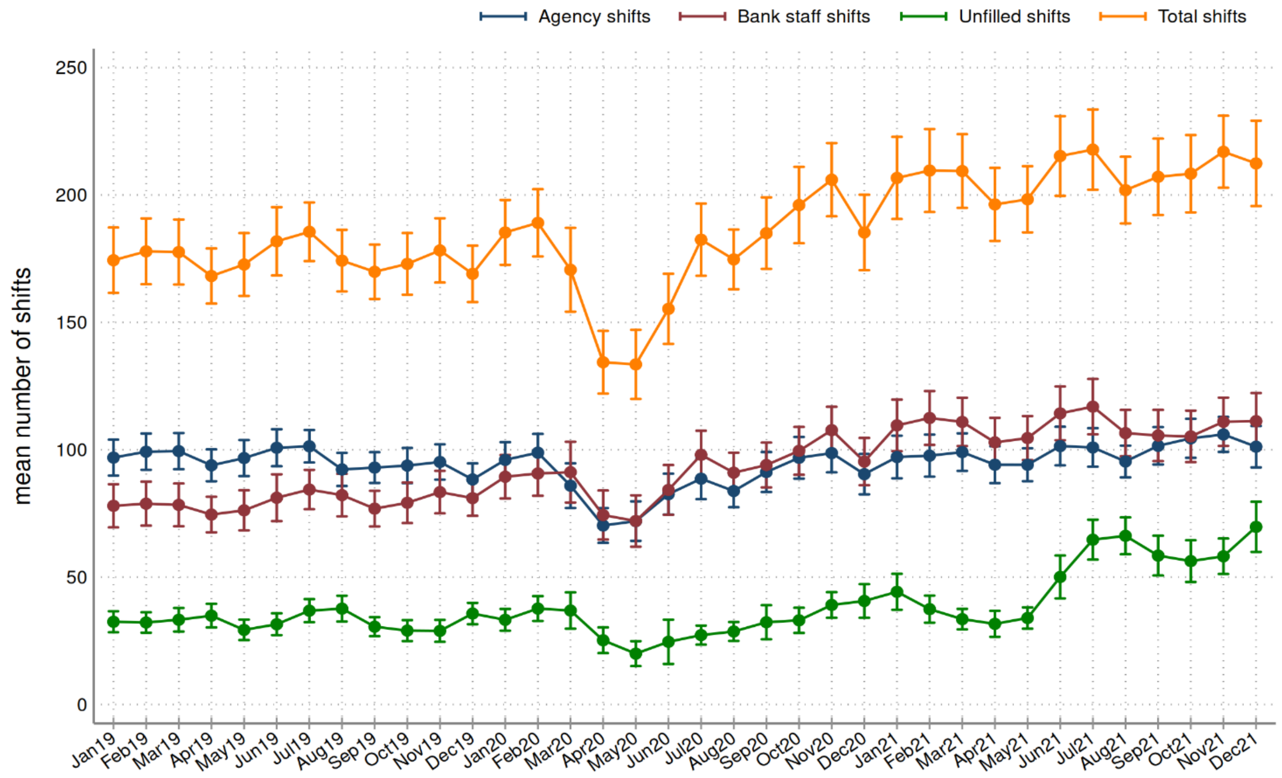

*dots indicate the mean number of shifts across trusts for each month in the period 2019 – 2021. and the vertical lines represent the upper and lower 95% confidence limits

± The number of trusts that reported data in 2019, 2020 and 2021 were 229, 224 and 221 respectively.

Supplementary material - The use of locum doctors in NHS Trusts in England. Analysis of routinely collected workforce data 2019 – 2021.

For peer review only

Contents

Additional databases ... 3
NHS Workforce statistics database ... 3
NHS Vacancy statistics ... 3
Health Regulators Ratings ... 3
English Indices of Deprivation ... 4
Spatial Maps ... 4
Tables ... 5
$Table 1: NHS Trusts with the highest and lowest locum intensity in England, 2019 ... 5
$Table 2: Negative binomial regression analyses for the three outcomes in 2019, acute and mental health trusts, IRR [a,b,c] ... 6
Figures ... 8
$Figure 1: Locum intensity, proportion of agency shifts and proportion of unfilled shifts in 2020, NHS Trust level ... 8
$Figure 2: Locum intensity, proportion of agency shifts and proportion of unfilled shifts in 2021, NHS Trust level ... 9
$Figure 3: Regional variation in locum intensity, proportion of agency shifts and proportion of unfilled shifts, 2020* ... 10
$Figure 4: Regional variation in locum intensity, proportion of agency shifts and proportion of unfilled shifts, 2021* ... 11
$Figure 5: Spatial map of locum intensity at the Sustainability and Transformation Partnerships (STP) level, England 2019 ... 12
$Figure 6: Spatial map of agency shifts at the Sustainability and Transformation Partnerships (STP) level, England 2019 ... 13
$Figure 7: Spatial map of unfilled shifts at the Sustainability and Transformation Partnerships (STP) level, England 2019 ... 14
References ... 15

For peer review only

Additional databases

NHS Workforce statistics database

NHS Digital collects monthly NHS Hospital and Community Health Service (HCHS) workforce statistics (1) for staff in NHS trusts and Clinical Commissioning Groups (CCGs) in England. The data are recorded within the Electronic Staff Record (ESR), which is a payroll and human resources system and contains staff records NHS employed staff in England since 2008. Data are available as headcount and full-time equivalents and for all months from September 2009 onwards and they represent an accurate summary of the validated data extracted from the NHS ESR system. We downloaded monthly data collections available from NHS Digital from December 2017 to December 2021. For each month and each NHS trust, we calculated the average total FTE across all available doctor categories in the NHS workforce database and matched the data to NHS improvement data for each trust in each month. The database also included monthly NHS trust level turnover data which were used in the negative binomial regression.

NHS Vacancy statistics

From NHS Digital, (2) we obtained trust level vacancy rates for the period January 2019 to December 2019. The series refers to vacancy FTE from providers which are available on a quarterly basis but recorded on a monthly basis. This monthly rate is defined as the total number of unfilled posts reported at the end of each respective reporting month. A vacancy is defined as a post that is unfilled by permanent or fixed-term staff. Some vacant posts may be filled by agency or temporary staff, but these posts are still considered to be vacancies. The number of vacancies is the difference between the number of reported full-time equivalent (FTE) permanent or fixed-term staff in post and planned workforce levels (i.e. the total funded or budgeted establishment on an FTE basis).

Health Regulators Ratings

Health regulators ratings were obtained from the Care Quality Commission (CQC). Each rating is based on the assessment of the evidence against the key lines of enquiry in the assessment framework for healthcare services and, for relevant non-specialist acute trusts, the use of resources assessment framework. The trust level ratings refer to the trust's overall quality, based on findings under five key quality questions that CQC inspects (safe, effective, caring, responsive and well-led). Where applicable, the CQC also awards a combined rating at the trust level, based on the findings of the five trust-level quality ratings plus a use of resources rating. (3, 4) Each year the CQC inspects NHS trusts,

For peer review only

and awards each of them one of four rating levels ('outstanding', 'good', 'requires improvement', or 'inadequate') in five domains ('safe', 'effective', 'responsive', 'caring', and 'well led'), along with an 'overall' rating that summarised the domain ratings. From the CQC, we obtained published inspection ratings and corresponding inspection dates for all 229 NHS trusts in 2019 that were inspected between January 2019 and December 2019. Over the inspection cycle, some NHS trusts were re-inspected and their ratings updated. Only the rating from the first inspection was used in this study, as subsequent ratings were likely to have been influenced by the previous inspection process and outcomes. Data on the most recent practice inspections are freely available online. (5)

English Indices of Deprivation

Area deprivation, as measured by the latest update of the Index of Multiple Deprivation (IMD) (i.e.2019) was available at the 2011 Lower Super Output (LSOA) level. The IMD measures relative levels of deprivation for all the 32,844 LSOAs in England on a continuous scale of deprivation where most of the indicators are based on 2012 statistics. It is a combined score of deprivation based on a total of 37 separate indicators that have been grouped into seven domains, each of which reflects a different aspect of deprivation experienced by individuals living in an area. The overall measure is calculated as a weighted mean across seven domains: income, employment, education and skills, health and disability, crime, barriers to housing and services, and living environment with different weights given to each domain. The Index of Multiple Deprivation is assigned to every small area in England and ranks them from 1 (most deprived area) to 32,844 (least deprived area). The IMD is widely used across central government to focus programmes on the most deprived areas. (6) These area measures were assigned to trust based on trusts' location. Trusts in our analysis are allocated an IMD score based on the mean IMD score of all admitted patients using data from Hospital Episode Statistics 2017. (7)

Spatial Maps

Digital vector boundaries for the 2019 STPs (Sustainability and Transformation Partnerships), generalised to 20 metres and clipped to the coastline to reduce size and improve visualisation, were obtained from the ONS open geography portal. (8) The vector boundaries were inputted in the Stata shp2dta (9) command to calculate the centroid for each STP in the British National Grid format. These were then converted from British National Grid easting and northing to longitude and latitude in degrees. (10)

For peer review only

Tables

$Table 1: NHS Trusts with the highest and lowest locum intensity in England, 2019

**Trusts with the highest locum intensity in England**	**Locum intensity***	**Trusts with the lowest locum intensity in England**	**Locum intensity***
North East London NHS Foundation Trust	0.795	Royal Papworth Hospital NHS Foundation Trust	0.001
Bedfordshire Hospitals Foundation Trust	0.768	Cambridgeshire Community Services NHS Trust	0.002
Rotherham Doncaster And South Humber NHS Foundation Trust	0.684	The Newcastle Upon Tyne Hospitals NHS Foundation Trust	0.004
Oxford Health NHS Foundation Trust	0.621	Moorfields Eye Hospital NHS Foundation Trust	0.009
Dudley And Walsall Mental Health Partnership NHS Trust	0.602	Alder Hey Children's NHS Foundation Trust	0.010
North Cumbria University Hospitals NHS Trust	0.595	North Tees And Hartlepool NHS Foundation Trust	0.014
George Eliot Hospital NHS Trust	0.591	University Hospitals Bristol And Weston NHS Foundation Trust	0.014
North Cumbria Integrated Care NHS Foundation Trust	0.551	Sheffield Children's NHS Foundation Trust	0.022
United Lincolnshire Hospitals NHS Trust	0.548	Leeds Community Healthcare NHS Trust	0.025
Pennine Acute Hospitals NHS Trust	0.548	Imperial College Healthcare NHS Trust	0.029

*Locum intensity is defined as the sum agency and bank locum shifts adjusted for the size of Trusts' permanent doctor workforce

For peer review only

$Table 2: Negative binomial regression analyses for the three outcomes in 2019, acute and mental health trusts, IRR [a,b,c]

	Locum Intensity	Agency shifts	Unfilled shifts
**Trust level aggregate FTE (reference group is quintile 1)**	**Reference group**	**Reference group**	**Reference group**
Quintile 2	0.499 (0.369 to 0.676), <0.001 (0.077)	1.044 (0.811 to 1.344), <0.735 (0.134)	2.189 (0.967 to 4.960), <0.060 (0.913)
Quintile 3	0.465 (0.321 to 0.676), <0.001 (0.088)	1.044 (0.760 to 1.434), <0.787 (0.169)	3.076 (1.179 to 8.023), <0.022 (1.504)
Quintile 4	0.486 (0.329 to 0.716), <0.001 (0.096)	0.937 (0.671 to 1.311), <0.708 (0.160)	2.572 (0.937 to 7.063), <0.067 (1.325)
Quintile 5	0.248 (0.157 to 0.390), <0.001 (0.057)	0.859 (0.574 to 1.287), <0.462 (0.177)	3.783 (1.237 to 11.572), <0.020 (2.158)
**Trust type (reference group is NHS non-specialist trust)**	**Reference group**	**Reference group**	**Reference group**
NHS specialist trust	0.254 (0.176 to 0.366), <0.001 (0.047)	1.600 (1.153 to 2.220), <0.005 (0.268)	0.321 (0.119 to 0.866), <0.025 (0.162)
Mental health trust	0.991 (0.643 to 1.233), <0.487 (0.147)	1.556 (1.180 to 2.052), <0.002 (0.219)	1.015 (0.457 to 2.256), <0.971 (0.414)
**CQC ratings (reference group is trusts that provide good and outstanding services)**	**Reference group**	**Reference group**	**Reference group**
Inadequate and requiring improvement	1.626 (1.365 to 1.938), <0.001 (0.145)	1.039 (0.899 to 1.201), <0.601 (0.077)	1.329 (0.855 to 2.066), <0.206 (0.299)
**Index of multiple deprivation**			

For peer review only

**(reference group is quintile 1, where 1 is the most deprived)**			
Quintile 2	1.072 (0.818 to 1.406), <0.610 (0.148)	1.077 (0.859 to 1.349), <0.520 (0.124)	0.585 (0.308 to 1.111), <0.101 (0.191)
Quintile 3	1.138 (0.855 to 1.514), <0.375 (0.166)	1.182 (0.936 to 1.494), <0.159 (0.141)	0.494 (0.248 to 0.986), <0.046 (0.174)
Quintile 4	1.153 (0.855 to 1.554), <0.350 (0.175)	1.198 (0.933 to 1.539), <0.156 (0.153)	0.948 (0.448 to 2.008), <0.890 (0.363)
Quintile 5	1.038 (0.741 to 1.455), <0.827 (0.179)	1.169 (0.882 to 1.550), <0.278 (0.168)	0.628 (0.286 to 1.381), <0.247 (0.252)
**Region (reference region is London)**	**Reference group**	**Reference group**	**Reference group**
South West	0.468 (0.328 to 0.668), <0.001 (0.085)	1.595 (1.198 to 2.123), <0.014 (0.233)	1.258 (0.510 to 3.103), <0.618 (0.580)
South East	0.746 (0.531 to 1.048), <0.092 (0.130)	1.498 (1.136 to 1.975), <0.006 (0.211)	0.578 (0.262 to 1.273), <0.174 (0.233)
Midlands	0.980 (0.718 to 1.337), <0.896 (0.155)	1.481 (1.149 to 1.908), <0.001 (0.192)	0.795 (0.371 to 1.700), <0.553 (0.308)
East of England	0.815 (0.579 to 1.147), <0.240 (0.142)	1.632 (1.233 to 2.161), <0.001 (0.234)	0.419 (0.177 to 0.988), <0.047 (0.184)
North West	0.917 (0.646 to 1.301), <0.627 (0.163)	1.289 (0.964 to 1.724), <0.101 (0.191)	1.176 (0.508 to 2.724), <0.704 (0.504)
North East and Yorkshire	0.703 (0.491 to 1.005), <0.053 (0.128)	1.450 (1.084 to 1.941), <0.001 (0.215)	0.753 (0.299 to 1.901), <0.549 (0.356)
constant	0.381 (0.221 to 0.656), <0.001 (0.105)	0.339 (0.219 to 0.524), <0.001 (0.075)	0.065 (0.017 to 0.247), <0.001 (0.045)

[a] All models included data on 187 trusts (observations) with robust standard errors.
[b] 95% confidence intervals are in brackets; results are reported as incidence rate ratios (IRR) followed by *P*-values and standard errors in parentheses.
[c] Coefficients can be interpreted as proportionate changes, for example, trusts in the North West had on average 8.3% lower locum intensity than trusts in London.

Figures

$Figure 1: Locum intensity, proportion of agency shifts and proportion of unfilled shifts in 2020, NHS Trust level		
a)Mean locum intensity (bank and agency shifts combined) at Trust level in 2020†*	b)Mean proportion of agency shifts at Trust level in 2020††	c)Mean proportion of unfilled shifts at Trust level in 2020¥
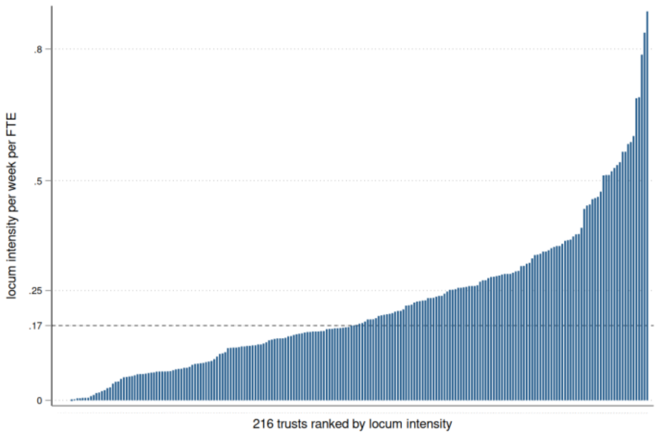	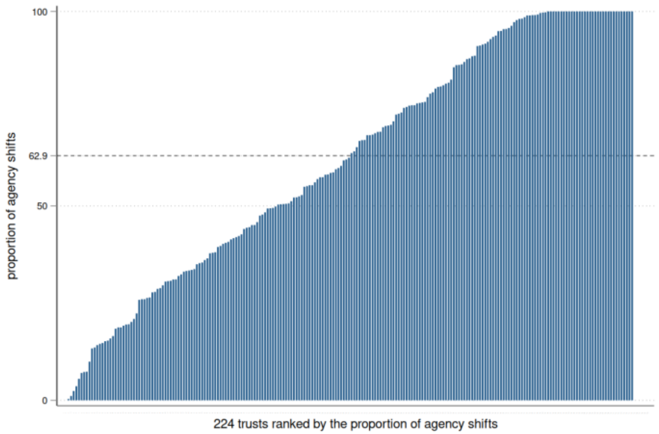	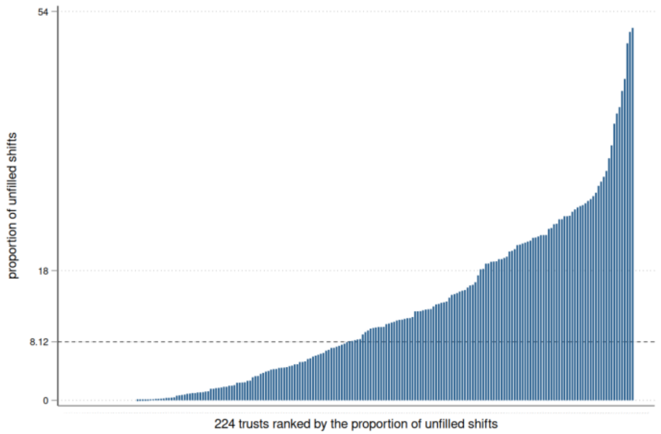
† dash line indicates the median (0.170) locum intensity per week per FTE across 216 NHS trusts in 2020<br>* eight trusts were excluded from the analyses due to very small or zero denominators (i.e. permanent doctor FTE)	†† dash line indicates the median (62.9%) proportion of agency shifts per week across 224 NHS trusts in 2020	¥ dash line indicates the median (8.12%) unfilled shift rate per week across 224 NHS trusts in 2020.

$Figure 2: Locum intensity, proportion of agency shifts and proportion of unfilled shifts in 2021, NHS Trust level

a)Mean locum intensity (bank and agency shifts combined) at Trust level in 2021†*

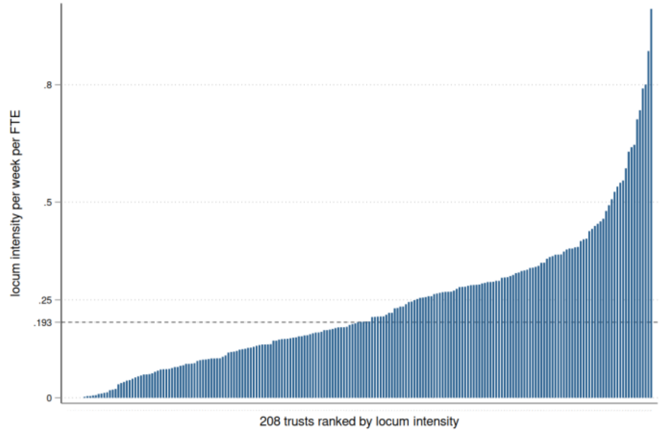

† dash line indicates the median (μ=0.193) locum intensity per week per FTE across 208 NHS trusts in 2021
* 13 trusts were excluded from the analyses due to very small or zero denominators (i.e. permanent doctor FTE)

b)Mean proportion of agency shifts at Trust level in 2021††

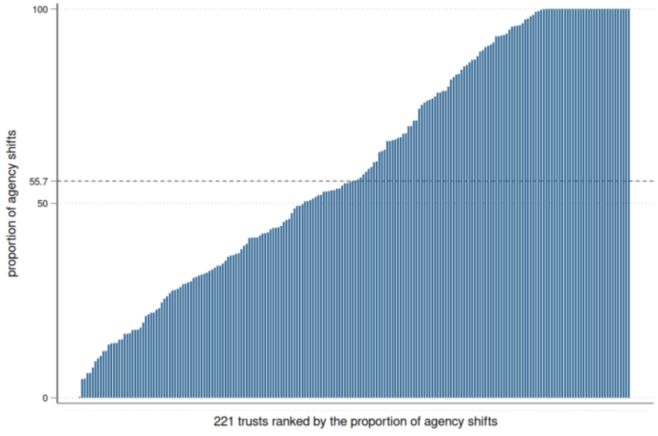

†† dash line indicates the median (μ=55.7%) proportion of agency shifts per week across 221 NHS trusts in 2021

c)Mean proportion of unfilled shifts at Trust level in 2021¥

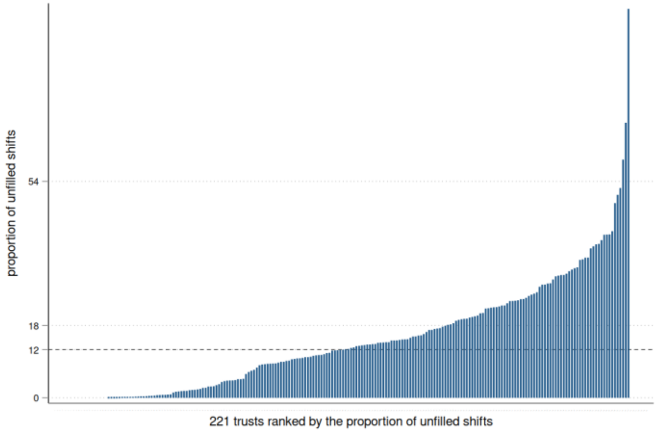

¥ dash line indicates the median (μ=12%) unfilled shift rate per week across 221 NHS trusts in 2021

$Figure 3: Regional variation in locum intensity, proportion of agency shifts and proportion of unfilled shifts, 2020*

a) Regional variation in locum intensity (bank + agency shifts combined) in 2020†**

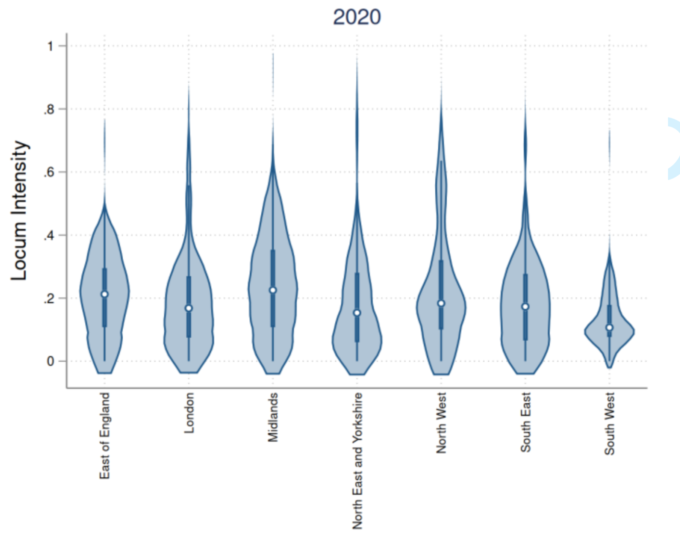

† Figure includes data from 218 NHS trusts in 2020, adjusted for substantive doctor FTE.

b) Regional variation in the proportion of agency shifts in 2020††

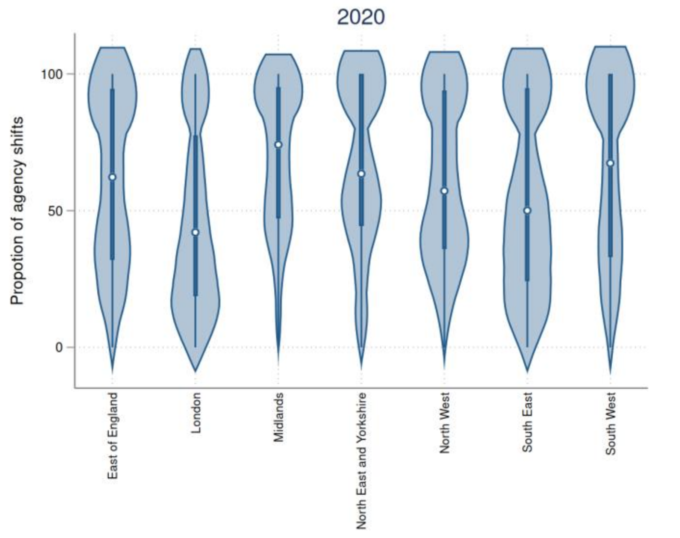

†† Figure includes data from 224 NHS trusts in 2020

c) Regional variation in the proportion of unfilled shifts in 2020¥

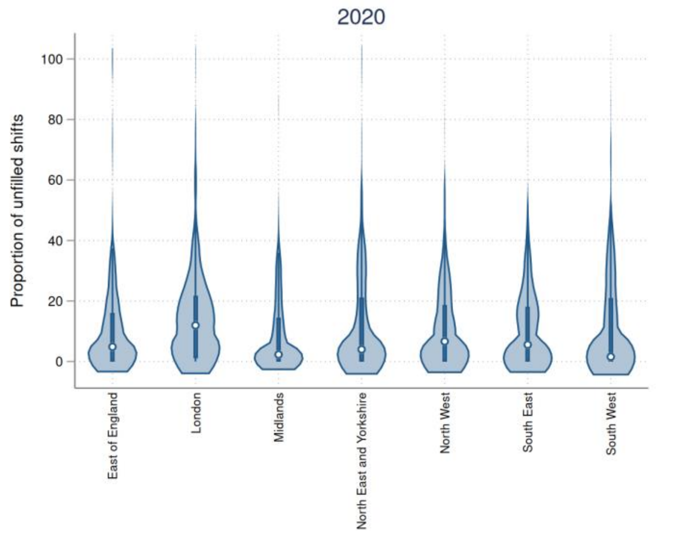

¥ Figure includes data from 224 NHS trusts in 2020

* The thick blue line represents the interquartile range (25th–75th centile) and the thin line represents the rest of the distribution with upper/lower adjacent values. The white dot represents the median of the data. The distribution shape of the data is based on a kernel density estimation where wider sections of the plot represent a higher chance that members of the population of interest will take on a given value and where thinner section represent lower chance.
** Six trusts were excluded from the analyses due to non-availability of data for substantive doctor FTE.

$Figure 4: Regional variation in locum intensity, proportion of agency shifts and proportion of unfilled shifts, 2021*

a) Regional variation in locum intensity (bank + agency shifts combined) in 2021†**

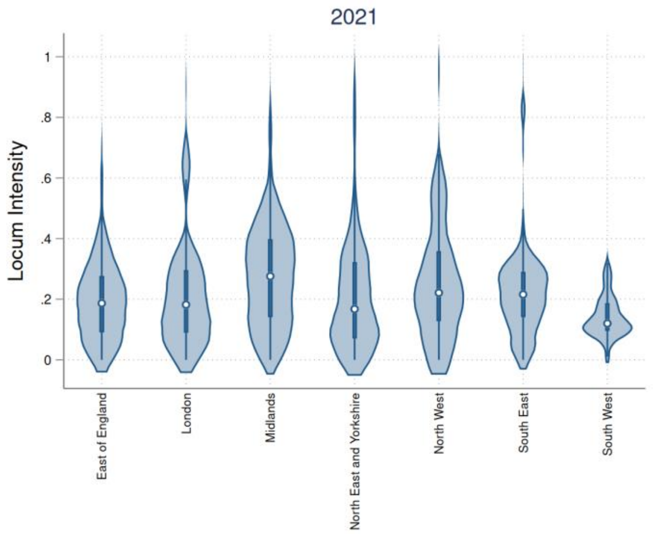

† Figure includes data from 210 NHS trusts in 2021, adjusted for substantive doctor FTE.

b) Regional variation in the proportion of agency shifts in 2021††

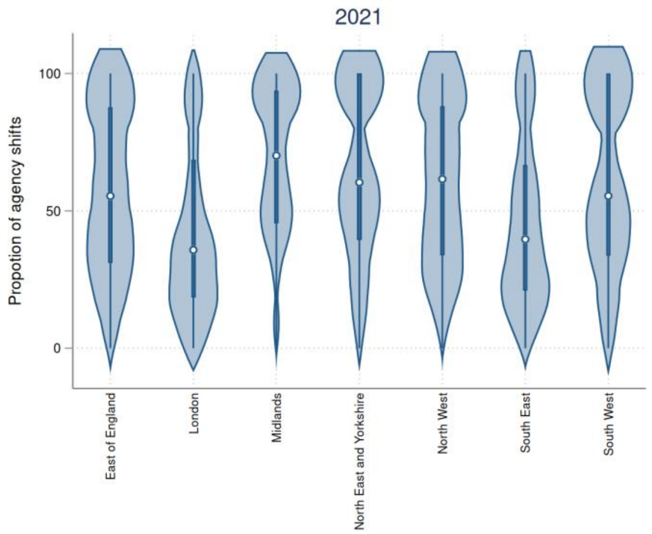

†† Figure includes data from 221 NHS trusts in 2021

c) Regional variation in the proportion of unfilled shifts in 2021¥

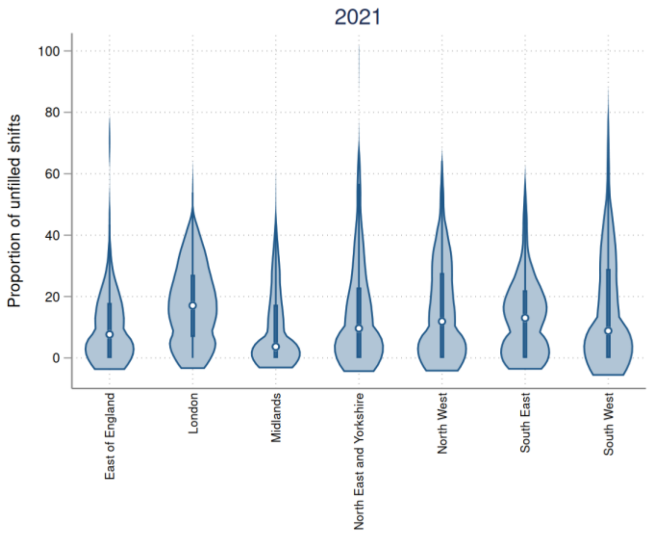

¥ Figure includes data from 221 NHS trusts in 2021

* The thick blue line represents the interquartile range (25th–75th centile) and the thin line represents the rest of the distribution with upper/lower adjacent values. The white dot represents the median of the data. The distribution shape of the data is based on a kernel density estimation where wider sections of the plot represent a higher chance that members of the population of interest will take on a given value and where thinner section represent lower chance.
**11 trusts were excluded from the analyses due to non-availability of data for permanent doctor FTE.

$Figure 5: Spatial map of locum intensity at the Sustainability and Transformation Partnerships (STP) level, England 2019

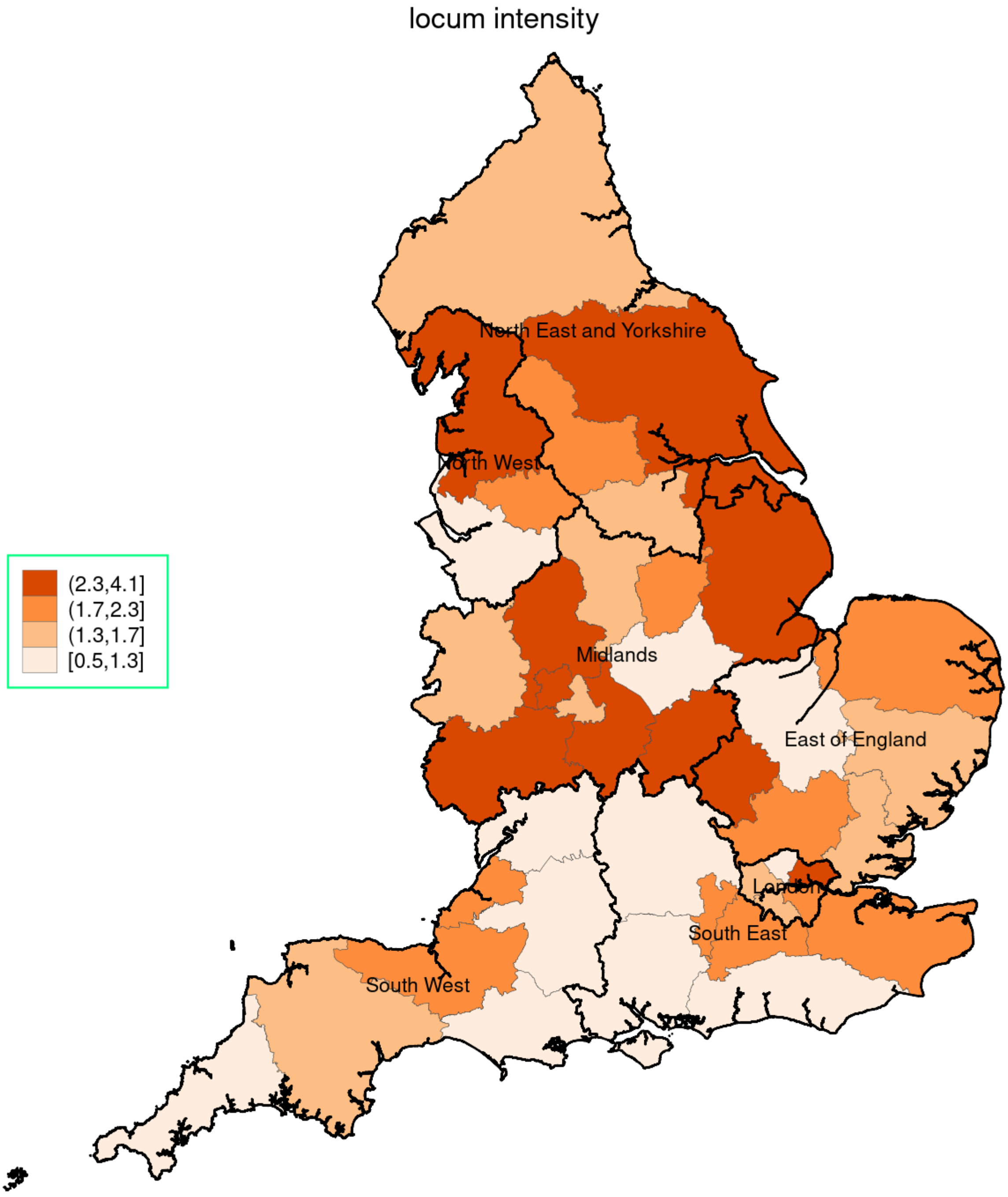

$Figure 6: Spatial map of agency shifts at the Sustainability and Transformation Partnerships (STP) level, England 2019

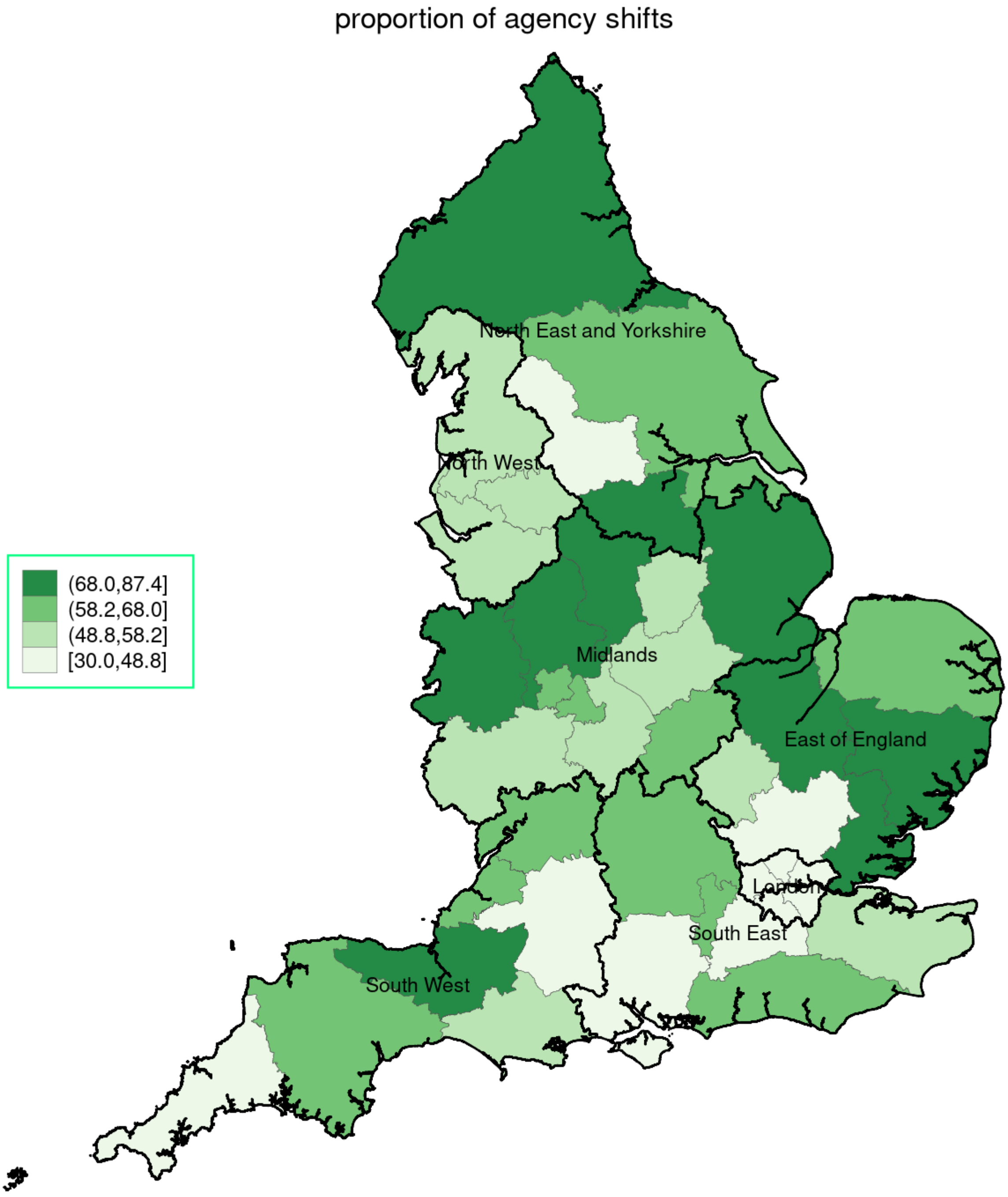

$Figure 7: Spatial map of unfilled shifts at the Sustainability and Transformation Partnerships (STP) level, England 2019

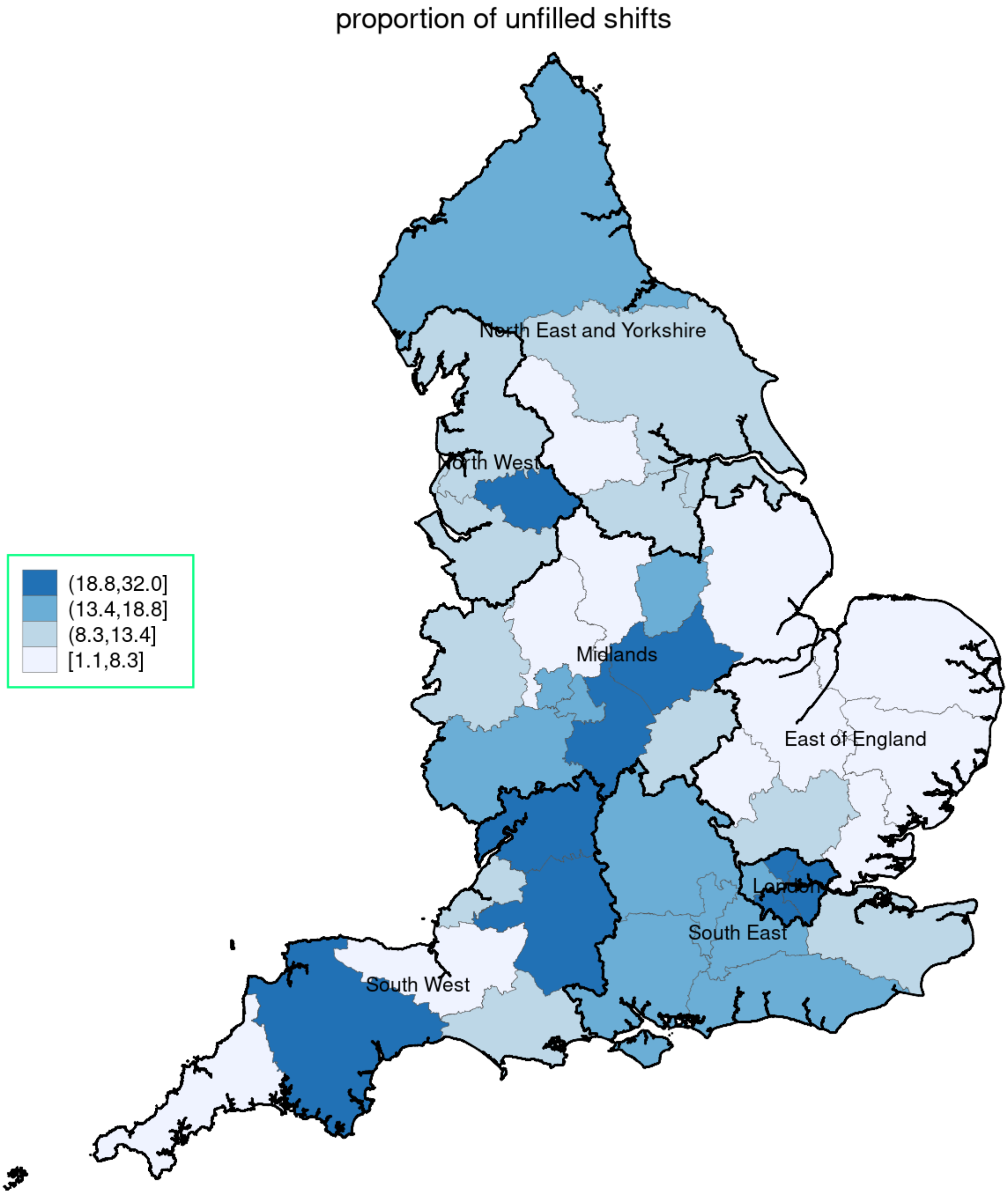

References

1. NHS Digital. NHS workforce statistics. 2022.
2. NHS Digital. NHS Vacancy Statistics. 2021.
3. Care Quality Commission. Shaping the Future: CQC's strategy 2016 to 2021. 2016.
4. Care Quality Commission. Intelligent Monitoring: NHS GP practices Indicators and methodology. 2014.
5. Care Quality Commission. Using CQC data. 2020.
6. Ministry of Housing, Communities and Local GovernmenT. The English Indices of Deprivation. Ministry of Housing, Communities and Local Government 2019.
7. NHS Digital. Hospital Episode Statistics. 2017.
8. Office for National Statistics. Open Geography Portal. 2018.
9. SHP2DTA: Stata module to convert shape boundary files to Stata datasets[program]. Boston College Department of Economics. 2006.;S456718 version.
10. Ordance Survey. A guide to coordinate systems in Great Britain. ONS, 2013:43. www.ordnancesurvey.co.uk/docs/support/guide-coordinate-systems-great-britainpdf.

For peer review only

STROBE Statement—checklist of items that should be included in reports of observational studies

	Item No.	Recommendation	Page No.	Relevant text from manuscript
**Title and abstract**	1	(*a*) Indicate the study's design with a commonly used term in the title or the abstract	1	
		(*b*) Provide in the abstract an informative and balanced summary of what was done and what was found	1	
**Introduction**				
Background/rationale	2	Explain the scientific background and rationale for the investigation being reported	2	
Objectives	3	State specific objectives, including any prespecified hypotheses	2	
**Methods**				
Study design	4	Present key elements of study design early in the paper	4-5	
Setting	5	Describe the setting, locations, and relevant dates, including periods of recruitment, exposure, follow-up, and data collection	5	
Participants	6	(*a*) *Cohort study*—Give the eligibility criteria, and the sources and methods of selection of participants. Describe methods of follow-up<br>*Case-control study*—Give the eligibility criteria, and the sources and methods of case ascertainment and control selection. Give the rationale for the choice of cases and controls<br>*Cross-sectional study*—Give the eligibility criteria, and the sources and methods of selection of participants	5	
		(*b*) *Cohort study*—For matched studies, give matching criteria and number of exposed and unexposed<br>*Case-control study*—For matched studies, give matching criteria and the number of controls per case		
Variables	7	Clearly define all outcomes, exposures, predictors, potential confounders, and effect modifiers. Give diagnostic criteria, if applicable	6	
Data sources/ measurement	8*	For each variable of interest, give sources of data and details of methods of assessment (measurement). Describe comparability of assessment methods if there is more than one group	5- 6	
Bias	9	Describe any efforts to address potential sources of bias	7	
Study size	10	Explain how the study size was arrived at	N/A	

Continued on next page

For peer review only - http://bmjopen.bmj.com/site/about/guidelines.xhtml
For peer review only

Quantitative variables	11	Explain how quantitative variables were handled in the analyses. If applicable, describe which groupings were chosen and why	6-7
Statistical methods	12	(*a*) Describe all statistical methods, including those used to control for confounding	7
		(*b*) Describe any methods used to examine subgroups and interactions	7
		(*c*) Explain how missing data were addressed	8
		(*d*) *Cohort study*—If applicable, explain how loss to follow-up was addressed<br>*Case-control study*—If applicable, explain how matching of cases and controls was addressed<br>*Cross-sectional study*—If applicable, describe analytical methods taking account of sampling strategy	7
		(*e*) Describe any sensitivity analyses	7
**Results**			
Participants	13*	(a) Report numbers of individuals at each stage of study—eg numbers potentially eligible, examined for eligibility, confirmed eligible, included in the study, completing follow-up, and analysed	8
		(b) Give reasons for non-participation at each stage	N/A
		(c) Consider use of a flow diagram	
Descriptive data	14*	(a) Give characteristics of study participants (eg demographic, clinical, social) and information on exposures and potential confounders	8
		(b) Indicate number of participants with missing data for each variable of interest	8
		(c) *Cohort study*—Summarise follow-up time (eg, average and total amount)	N/A
Outcome data	15*	*Cohort study*—Report numbers of outcome events or summary measures over time	
		Case-control study—Report numbers in each exposure category, or summary measures of exposure	
		Cross-sectional study—Report numbers of outcome events or summary measures	8
Main results	16	(*a*) Give unadjusted estimates and, if applicable, confounder-adjusted estimates and their precision (eg, 95% confidence interval). Make clear which confounders were adjusted for and why they were included	9-10
		(*b*) Report category boundaries when continuous variables were categorized	N/A
		(*c*) If relevant, consider translating estimates of relative risk into absolute risk for a meaningful time period	N/A

Continued on next page

For peer review only

Other analyses	17	Report other analyses done—eg analyses of subgroups and interactions, and sensitivity analyses	10
**Discussion**			
Key results	18	Summarise key results with reference to study objectives	11
Limitations	19	Discuss limitations of the study, taking into account sources of potential bias or imprecision. Discuss both direction and magnitude of any potential bias	11
Interpretation	20	Give a cautious overall interpretation of results considering objectives, limitations, multiplicity of analyses, results from similar studies, and other relevant evidence	12
Generalisability	21	Discuss the generalisability (external validity) of the study results	12
**Other information**			
Funding	22	Give the source of funding and the role of the funders for the present study and, if applicable, for the original study on which the present article is based	13

*Give information separately for cases and controls in case-control studies and, if applicable, for exposed and unexposed groups in cohort and cross-sectional studies.

Note: An Explanation and Elaboration article discusses each checklist item and gives methodological background and published examples of transparent reporting. The STROBE checklist is best used in conjunction with this article (freely available on the Web sites of PLoS Medicine at http://www.plosmedicine.org/, Annals of Internal Medicine at http://www.annals.org/, and Epidemiology at http://www.epidem.com/). Information on the STROBE Initiative is available at www.strobe-statement.org.

For peer review only

For peer review only - http://bmjopen.bmj.com/site/about/guidelines.xhtml

BMJ Open

The use of locum doctors in NHS trusts in England: analysis of routinely collected workforce data 2019 – 2021.

Journal:	*BMJ Open*
Manuscript ID	bmjopen-2022-065803.R1
Article Type:	Original research
Date Submitted by the Author:	25-Dec-2022

Complete List of Authors:	Grigoroglou, Christos; The University of Manchester, Manchester Centre for Health Economics<br>Walshe, K; The University of Manchester, Alliance Manchester Business School, Institute for Health Policy and Organisation<br>Kontopantelis, Evangelos; The University of Manchester, NIHR School for Primary Care Research, Centre for Primary Care, Division of Population Health, Health Services Research and Primary Care; The University of Manchester, Division of Informatics, Imaging and Data Sciences<br>Ferguson, Jane ; The University of Manchester, Alliance Manchester Business School, Institute for Health Policy and Organisation<br>Stringer, Gemma; The University of Manchester, Alliance Manchester Business School, Institute for Health Policy and Organisation<br>Ashcroft, Darren; The University of Manchester, NIHR School for Primary Care Research, Centre for Primary Care, Division of Population Health, Health Services Research and Primary Care; The University of Manchester, NIHR Greater Manchester Patient Safety Translational Research Centre<br>Allen, Thomas; The University of Manchester, Manchester Centre for Health Economics; University of Southern Denmark

**Primary Subject Heading**:	Health services research
Secondary Subject Heading:	Health policy
Keywords:	Health policy < HEALTH SERVICES ADMINISTRATION & MANAGEMENT, Human resource management < HEALTH SERVICES ADMINISTRATION & MANAGEMENT, Organisation of health services < HEALTH SERVICES ADMINISTRATION & MANAGEMENT

SCHOLARONE™
Manuscripts

BMJ

I, the Submitting Author has the right to grant and does grant on behalf of all authors of the Work (as defined in the below author licence), an exclusive licence and/or a non-exclusive licence for contributions from authors who are: i) UK Crown employees; ii) where BMJ has agreed a CC-BY licence shall apply, and/or iii) in accordance with the terms applicable for US Federal Government officers or employees acting as part of their official duties; on a worldwide, perpetual, irrevocable, royalty-free basis to BMJ Publishing Group Ltd ("BMJ") its licensees and where the relevant Journal is co-owned by BMJ to the co-owners of the Journal, to publish the Work in this journal and any other BMJ products and to exploit all rights, as set out in our licence.

The Submitting Author accepts and understands that any supply made under these terms is made by BMJ to the Submitting Author unless you are acting as an employee on behalf of your employer or a postgraduate student of an affiliated institution which is paying any applicable article publishing charge ("APC") for Open Access articles. Where the Submitting Author wishes to make the Work available on an Open Access basis (and intends to pay the relevant APC), the terms of reuse of such Open Access shall be governed by a Creative Commons licence – details of these licences and which Creative Commons licence will apply to this Work are set out in our licence referred to above.

Other than as permitted in any relevant BMJ Author's Self Archiving Policies, I confirm this Work has not been accepted for publication elsewhere, is not being considered for publication elsewhere and does not duplicate material already published. I confirm all authors consent to publication of this Work and authorise the granting of this licence.

For peer review only

The use of locum doctors in NHS trusts in England: analysis of routinely collected workforce data 2019 – 2021.

Christos Grigoroglou[1] (christos.grigoroglou@manchester.ac.uk)

Kieran Walshe[2] (kieran.walshe@manchester.ac.uk)

Evangelos Kontopantelis[3, 4] (e.kontopantelis@manchester.ac.uk)

Jane Ferguson[2] (jane.ferguson@manchester.ac.uk)

Gemma Stringer[2] (gemma.stringer@manchester.ac.uk)

Darren M Ashcroft[3, 5 6] (darren.ashcroft@manchester.ac.uk)

Thomas Allen[1, 7] (thomas.allen@manchester.ac.uk)

1. Manchester Centre for Health Economics, Division of Population Health, Health Services Research and Primary Care, University of Manchester, Manchester, UK,
2. Alliance Manchester Business School, Institute for Health Policy and Organisation, University of Manchester, Manchester, UK
3. NIHR School for Primary Care Research, Centre for Primary Care, Division of Population Health, Health Services Research and Primary Care, University of Manchester, Manchester, UK
4. Division of Informatics, Imaging and Data Sciences, University of Manchester, Manchester, UK
5. NIHR Greater Manchester Patient Safety Translational Research Centre, University of Manchester, Manchester, UK
6. Centre for Pharmacoepidemiology and Drug Safety, School of Health Sciences, Faculty of Biology, Medicine and Health, University of Manchester, Manchester, UK
7. Danish Centre for Health Economics, University of Southern Denmark, Denmark

Corresponding author: Christos Grigoroglou (christos.grigoroglou@manchester.ac.uk)

For peer review only - http://bmjopen.bmj.com/site/about/guidelines.xhtml

Abstract

Objectives: Temporary doctors, known as locum doctors play an important role in the delivery of care in the NHS, however, little is known about the extent of locum use in NHS trusts. This study aimed to quantify and describe locum use for all NHS trusts in England in 2019–2021.

Setting: Descriptive analyses of data on locum shifts from all NHS trusts in England in 2019-2021. Weekly data were available for the number of shifts filled by agency and bank staff and the number of shifts requested by each trust. Negative binomial models were used to investigate the association between the proportion of medical staffing provided by locums and NHS trust characteristics.

Results: In 2019, on average 4.4% of total medical staffing was provided by locums, but this varied substantially across trusts (25th–75th centile=2.2% to 6.6%). Over time, on average two thirds of locum shifts were filled by locum agencies and a third by trusts' staff banks. On average 10% of shifts requested were left unfilled. In 2019-2021, the mean number of weekly shifts per trust increased by 17% (175.2 to 208.6) and the mean number of weekly unfilled shifts per trust increased by 52.2% (32.8 to 49.95). Trusts rated by the Care Quality Commission as inadequate or requiring improvement (IRR=1.495; 1.191 to 1.877 95% CI), and smaller trusts had a higher use of locums. Large variability was observed across regions for use of locums, proportion of shifts filled by locum agencies, and unfilled shifts.

Conclusions: There were large variations in the demand for and use of locum doctors in NHS trusts. Trusts with poor CQC ratings and smaller trusts appear to use locum doctors more intensively compared to other trust types. Unfilled shifts were at a three-year high at the end of 2021 suggesting increased demand which may result from growing workforce shortages in NHS trusts

Keywords: employment, locum doctors, health workforce, regional variation, NHS trusts

Word count: 3,931 words

For peer review only - http://bmjopen.bmj.com/site/about/guidelines.xhtml

Strengths and limitations of the study
• National study covering every NHS trust in England.
• Data on locum doctors across NHS trusts have recently become available for the first time and this research study utilised this dataset.
• Outcomes investigated included measures of locum use across trusts as well as outcomes pertaining to how trusts recruit locum doctors and how well trusts are able to fill their needs with locum doctors.
• The data lacks some important information such as the types of medical specialties covered by locum doctors which would provide an improved understanding of the work that locums do.
• Information on length of locum shifts would enable us to capture more accurately the level of locum intensity at the NHS trust level.

For peer review only

For peer review only - http://bmjopen.bmj.com/site/about/guidelines.xhtml

Introduction

In the UK, challenges in the recruitment and retention of medical staff, including doctors of all grades, consultants, registrars and other doctors in training, in the National Health System (NHS) have resulted in significant staff shortages. (1-4) In 2018, 43% of NHS consultant posts in general medicine which were advertised were not filled and 40% of consultants and 63% of higher specialist trainees said that rota gaps occurred on a daily or weekly basis. (5), while recent surveys of middle grade doctors reported that their workload has become unsustainable under current staffing levels. (4)

When faced with medical workforce shortages, NHS trusts can fill shifts using locum doctors recruited through locum agencies, third party organisations who contract healthcare professionals to work temporarily within the NHS, or through internal staff banks. Increases in NHS expenditure for agency staff led to NHS Improvement introducing a locum pay cap to curb agency expenditure and a weekly system for gathering data on locum usage by NHS trusts in 2016. (6) The new set of rules for agency staff resulted in a reduction in spending on locum agency doctors from £3.6 billion in 2015/16 to £2.38 billion in 2019/20 (7), although many hospital trusts have applied for extensions of these price caps to fill their workforce gaps. (4) Despite national information on expenditure, little is known about the extent of locum doctors working across NHS trusts, in contrast to general practice where NHS Digital has published workforce data since 2015. (8, 9) To date, no study has described the scale of locum usage in NHS trusts in England.

The aim of this study was to use NHS Improvement data to quantify and describe locum use, and its variation, for all acute, ambulance, community and mental health NHS trusts in England from January 2019 to December 2021. We describe the rate at which NHS trusts were able to fill locum shifts and whether NHS trusts find their locum workforce via their own NHS staff banks or via locum agencies. We explore regional variations for these measures and identify NHS trusts with the highest and lowest locum usage in 2019, as this was the year before the onset of the COVID-19 pandemic which was a period of substantial disruptions in the delivery of NHS services. Finally, we examine whether some NHS trust and population characteristics explain variability in locum use at the trust level in 2019.

Methods

Patient and Public Involvement
No patient involved

Data

NHS trust temporary staff employment data

For peer review only - http://bmjopen.bmj.com/site/about/guidelines.xhtml

In England, NHS Improvement is responsible for setting out rules which trusts are expected to follow on temporary staff expenditure. The rules have a strong focus on providing support to trusts to reduce their expenditure and to move towards a sustainable model of temporary staffing. To fulfil this responsibility and support trusts, NHS Improvement collects information from all NHS trusts on their employment of temporary staff. These data are not published and were secured for research through a bespoke data-sharing agreement.

We analysed data on locum use for all NHS trusts in England between January 2019 and December 2021. Data record the weekly number of shifts that were filled by bank or agency locums for each acute, ambulance, community and mental health trust in England. A shift is defined as the period between the doctor commencing and finishing their work but the duration of shifts is not collected. Bank staff are defined as staff who are usually sourced in-house or from temporary staff banks such as NHS Professionals, which is the largest of these banks supplying temporary staff to NHS trusts. (10) Agency staff are defined as staff who are not on the payroll of the NHS organisation offering employment and are sourced from a third-party agency.

NHS Improvement data record information on the number of shifts filled by temporary staff in all staff groups but we focus on the medical and dental staff group which includes the aggregate number of shifts, filled by temporary doctors working in NHS trusts. The data capture a snapshot of the weekly number of shifts done by doctors within hospital and community services (HCHS) of the NHS, who are defined as all practising doctors who are registered with the General Medical Council (GMC) – including GPs and dental staff – who are employed substantively by trust i.e. are on a trust's payroll. Information on the total number of doctor shifts that were filled by bank staff, the total number of shifts filled by agency staff and the total number of shifts requested by each trust in every reporting week, was provided. A detailed table of all the variables in the NHS Improvement data is provided in the supplementary material.

NHS trust characteristics

We collected monthly data on all trusts' substantive employees represented as full-time equivalents (FTE) and trust annual job turnover data for the medical and dental staff group using the NHS Workforce Statistics database. (11) Trust type information and trust overall inspection ratings were obtained from the Care Quality Commission (CQC) which rates NHS trusts as outstanding, good, requiring improvement or inadequate. (12) Trust level deprivation was derived using hospital admissions data from NHS Digital and aggregating inpatient postcode deprivation for each trust. (13) Trust level vacancy rates were obtained in the form of advertised FTE roles for medical and dental staff, available from the NHS Vacancy Statistics from NHS Digital. (14) These trust characteristics were linked to temporary staffing data using unique trust identifiers and are discussed in detail in the supplementary material.

For peer review only - http://bmjopen.bmj.com/site/about/guidelines.xhtml

Analyses
Outcome measures

Locum intensity

Our primary outcome measured locum intensity for each NHS trust in every reporting week. To calculate locum intensity, we combined bank and agency shifts to obtain the total number of shifts reported at trust level in every reporting week. We adjusted this weekly total by the size of the permanent medical and dental workforce in each trust, specifically, the total number of locum shifts was divided by permanent HCHS doctor FTE, (i.e. FTE of NHS and Community Health Hospital Doctors, Consultants, Associate Specialists, Specialty Doctors, Specialty Registrars, Foundation Doctors/Postgraduate Doctors) to give the locum intensity. The annual mean locum intensity was calculated over 12 months of data. A locum intensity of 0.25 indicates that the trust filled 0.25 locum shifts per week per FTE permanent doctor. We report locum intensity in this way because we do not know the length of the reported locum shifts and therefore cannot directly convert them into FTE. If we assume that one FTE permanent doctor typically works five shifts per week and that shift length for permanent doctors and locum doctors is broadly equivalent, then a locum intensity of 0.25 means that 5% of medical staffing in that week was provided by locums. We present a worked example of the algorithm that we used in the calculation of each outcome in Box 1.

Box 1: Worked example of outcome measure calculations for Manchester University NHS Foundation trust in 2019.

To obtain the mean locum intensity for Manchester University NHS Foundation trust in 2019, we combined the number of bank and agency shifts to calculate the total number of filled shifts out of the number of shifts requested. For every reporting week in 2019, we divided the total number of shifts by the monthly permanent doctor FTE reported the in that month. For example, in the week commencing 7th January 2019, Manchester University NHS Foundation trust reported 205 agency shifts and 283 bank staff shifts. We divided the total number of shifts (i.e. 488) by the reported permanent doctor FTE in January (i.e. 4,378.8) to obtain a locum intensity value of 0.11, suggesting that for every one full-time doctor the trust had 0.11 locum doctor shifts that week. That would equate to 2.2% [(0.11/5)*100] of care provided by locums in that week if we assume five shifts per FTE.

We calculated the proportion of shifts filled by agency staff by dividing the total number of agency shifts by the total number of all shifts (agency and bank) for each trust in every reporting week. For example, the proportion of agency shifts for Manchester University NHS Foundation trust in the week commencing 7th January 2019 was (205/488)*100=42%.

We also had information on the number of shifts that each trust requested in every reporting week. For the same week, Manchester University NHS Foundation trust

For peer review only - http://bmjopen.bmj.com/site/about/guidelines.xhtml

requested 574 bank and agency shifts but failed to fill 86 of these giving an unfilled rate of 15% [(86/574)*100].

Proportion of agency shifts

Our second outcome measured trusts' reliance on agency staff, which are more costly than bank staff. We divided the number of agency shifts by the total number of filled shifts for every trust in every reporting week. An annual mean proportion of agency shifts was then calculated for each trust over 12 months of data.

Proportion of unfilled shifts

Our third outcome measures shifts that the trust was unable to fill. The total number of shifts requested by each trust in every week was provided by NHS Improvement. The number of filled shifts was subtracted from the number of shifts requested to obtain the number of unfilled shifts for each trust in each week. We calculated the proportion of unfilled shifts by dividing unfilled shifts by shifts requested. An annual mean proportion of unfilled shifts was calculated for each trust over 12 months of data. Trusts occasionally reported a higher number of shifts filled than requested. In these cases, we adjusted the number of shifts requested to reflect the number of total shifts filled in that week. These adjustments were made 811 times out of 11,450 (7.1%) trust-week observations in 2019.

Our analysis dataset contained information on locum intensity, proportion of agency shifts, proportion of unfilled shifts and trust characteristics for 229 acute, mental health, ambulance and community health trusts in 2019. Of these, three acute trusts and one ambulance trust did not report data on monthly doctor FTE, and one acute trust and seven ambulance trusts reported zero weekly locum returns in every reporting week. Eight ambulance, one acute, one mental health and one community trust reported zero agency shifts in every reporting week. We also explored variation in the three outcomes over time, with 224 and 221 trusts reporting bank and agency shift data to NHS Improvement, in 2020 and 2021 respectively.

Our first set of analyses was descriptive and we used ordered bar charts to show the distribution of locum intensity, proportion of agency shifts and proportion of unfilled shifts for all trusts in 2019 - 2021. Violin plots showed the geographic variation in each outcome across regions. We used spatial maps to illustrate the distribution of each outcome across all Sustainability and Transformation Partnerships (STPs), local partnerships aiming to improve health and quality of care in the areas they serve. Analysis from 2019 to 2021 uncovered whether trusts reported changes over time in locum intensity, proportion of agency shifts and

For peer review only - http://bmjopen.bmj.com/site/about/guidelines.xhtml

proportion of unfilled shifts, a period including a majority of the COVID 19 pandemic in England.

Our second set of analyses was inferential and employed three mean-dispersion negative binomial regressions to model locum intensity, proportion of agency shifts, and proportion of unfilled shifts in 2019. Each model used robust standard errors with fixed-effects predictors for region (as categorical, to account for between region variation) and outcome-specific offset to model the rate of events for each outcome. Our dependent variables were: the mean number of locum shifts (offset: natural logarithm of mean permanent doctor FTE to model the rate of locum shifts per permanent doctor FTE); the mean number of agency shifts (offset: natural logarithm of the mean total shifts to model the rate at which a shift is filled by agency staff); and the mean number of unfilled shifts (offset: natural logarithm of mean shifts requested to model the rate at which a requested shift is left unfilled). Offsets are used as each dependent variable is derived from count data, where the value of the count is determined by the size of the workforce or exposure to locums. Our choice of negative binomial models over standard Poisson models was based on the presence of over-dispersion in the three outcomes. We controlled for CQC inspection rating, trust type (NHS general acute trusts, NHS specialist trusts, mental health trusts, and ambulance trusts), trust size (quintiles of trust permanent doctor FTE), turnover and vacancy rates and regional effects. Marginal effects were also calculated for the statistically significant coefficients, to estimate the absolute effects of those predictors on shift coverages.

The final dataset consisted of 197 trusts out of 229 trusts in 2019 with complete data for all covariates (8.6% of data were missing). We performed a sensitivity regression analysis excluding 25 ambulance and community trusts as these trusts tend to employ very small numbers of doctors relative to acute and mental health NHS trusts. The exclusion of ambulance and community trusts allowed us to examine the effects of deprivation, which could only be measured for acute and mental health NHS trusts. Stata v16.1 was used for the principal data cleaning, management and analyses. We used the *nbreg* command with the exposure option.

Results

Overall locum use

In 2019, total unadjusted locum use for all trusts in England was 2,004,485 shifts, of which 909,029 (45.3%) were bank shifts and 1,095,455 (54.7%) were agency shifts. Trusts requested 2,316,302 shifts with a trust mean of 208 per week (SD=258.3). The completeness of the data was good with 99% of all trusts reporting at least some locum use in any week.

Locum intensity

For peer review only - http://bmjopen.bmj.com/site/about/guidelines.xhtml

Figure 1 plots the ranked mean locum intensity in 2019 for 219 NHS trusts in England showing significant variation in locum use across trusts. Mean locum intensity was 0.22 (SD=0.16) (median=0.195; 25th–75th centile=0.11 to 0.31) in 2019, indicating 0.22 locum shifts per permanent doctor FTE. Assuming five shifts per permanent doctor FTE, the average trust level locum intensity of 0.22 locum shifts per permanent doctor FTE was equivalent to 4.4% (i.e. [0.22/5]*100) (25th–75th centile=2.2% to 6.2%) of medical staffing provided by locums in 2019. Four ambulance trusts, three acute trusts and one community trust were not included in this analysis as they reported very low or zero permanent doctor FTE and therefore adjustments in their locum intensity could not be performed. The ranked rates of locum intensity in 2020 and 2021 are presented in supplementary figure 1. We report the ten trusts with the highest and lowest reported locum intensity in 2019 in the supplementary material (Supplementary Table 1).

Proportion of agency shifts

The proportion of locum shifts filled by locum agency staff (rather than from staff banks) ranked from low to high at the trust level is depicted in Figure 1b. The use of agency shifts varied substantially across trusts in 2019 with a mean of 66.1% (SD=28.5%; median=68.9%; 25th–75th centile=43.5% to 95.8%). Half of trusts (109) reported 100% of shifts filled by agency staff at some point in 2019, of which 24 trusts reported that shifts were filled entirely by agency staff in every week. Eight ambulance, one acute, one mental health and one community trusts reported zero agency shifts in every reporting week in 2019. We present the ranked proportion of agency shifts for 2020 and 2021 in supplementary figure 1.

Proportion of unfilled shifts

In figure 1c, trusts are ranked from low to high on their proportion of unfilled shifts. Overall, trusts were able to fill the majority of their requested shifts either via bank or agency staff but we observed substantial variation. The mean proportion of unfilled shifts was 11.3% (SD=11.9%; Median=7.23%; 25th–75th centile =0 to 18.1%). Seven ambulance and one acute trust did not request any shifts at any point in 2019. The ranked proportions of unfilled shifts for 2020 and 2021 are presented in supplementary figure 1.

Regional variation in locum use

In table 1, we present descriptive statistics on outcomes and trust characteristics at the regional level for 2019. Figures 2a, 2b and 2c show regional variation in outcomes at the trust level in 2019. At the regional level, median locum intensity varied substantially from 0.13 (25th–75th centile: 0.08 to 0.2) in the South West of England to 0.26 (25th–75th centile: 0.15 to 0.35) in the Midlands (Table 1 and figure 2a). We also observed large variation in the proportion of agency shifts across regions. Trusts in London filled the lowest proportion of shifts using agency staff with a median of 44.8%, (25th–75th centile=26.6% to 87.5%) while

For peer review only - http://bmjopen.bmj.com/site/about/guidelines.xhtml

trusts in the East of England filled the highest with a median of 78.1% (25th–75th centile=37% to 98.1) (Table 1 and figure 2b). Trusts in the East of England filled requested shifts more successfully with unfilled shifts of 3.25% (25th–75th centile: 0% to 13.1%) whereas trusts in London had unfilled shifts of 11.6% (25th–75th centile: 0.71% to 22.8%) (Table 1 and figure 2). Regional variation for the three outcomes in 2020 and 2021 is presented in supplementary tables 2 and 3, and supplementary figure 2.

Table 1 - Descriptive statistics in 2019, by region*†

	East of England	London	Midlands	North East & Yorkshire	North West	South East	South West
Locum intensity *, Median (25th–75th centile)	**0.19 (0.13 to 0.30)**	**0.18 (0.08 to 0.28)**	**0.26 (0.15 to 0.35)**	**0.17 (0.08 to 0.35)**	**0.21 (0.12 to 0.31)**	**0.19 (0.11 to 0.27)**	**0.13 (0.08 to 0.2)**
Proportion of agency shifts (%) Median (25th–75th centile)	**78.1 (37 to 98.1)**	**44.8 (26.6 to 87.5)**	**75.6 (54.9 to 94.2)**	**74.7 (51 to 99.5)**	**65.3 (45.2 to 90.8)**	**60 (34.3 to 88.9)**	**77.9 (33.4 to 100)**
Proportion of unfilled shifts † (% of requested shifts), Median (25th–75th centile)	**3.25 (0 to 13.1)**	**11.6 (0.71 to 22.8)**	**3.5 (0 to 16)**	**3.9 (0 to 23)**	**6.5 (0 to 21.6)**	**4.8 (0 to 19.5)**	**5 (0 to 17.6)**
Full-time doctor FTE, Median (25th–75th centile)	803.3 (385.4 to 1,176.5)	869.1 (398.9 to 2,061.5)	569 (220 to 1,198)	715.5 (268 to 1,246.1)	612.6 (321.9 to 1,016.4)	1,013 (298 to 1,317.7)	701.1 (229 to 1,082.2)
Trust types							
NHS general acute trusts (n)	16	18	21	23	20	17	17
Acute - NHS specialist trusts (n)	1	5	3	1	6	1	-

For peer review only - http://bmjopen.bmj.com/site/about/guidelines.xhtml

Mental health trusts (n)	4	10	12	9	6	5	6
Community health (n)	3	2	4	1	2	5	1
Ambulance service (n)	1	1	2	2	1	2	1

* Locum intensity is adjusted for mean total full-time doctor FTE in 2019
† The proportion of unfilled shifts for trusts that reported a higher number of shifts filled than shifts requested was capped at 100%.

We investigated spatial variation within and between regions using spatial maps at the Sustainability and Transformation Partnership level (see supplementary figures 3, 4 and 5). Substantial variability was observed for all three outcome both within and between regions. High levels of locum intensity were concentrated in the Midlands, the North East & Yorkshire, and the North West. The South East and South West ranked among the lowest in terms of locum intensity. High proportions of agency shifts were observed in areas in the Midlands, the East of England, and the North East & Yorkshire. London had by far the lowest proportion of agency shifts. The proportion of unfilled shifts was high in areas in London, the Midlands and the South West and low in the East of England.

Results from regression analyses

The regression analyses results using the three different outcomes are presented in table 2. The results are reported as incidence rate ratios (IRRs) for the coefficients of interest followed by P-values and standard errors in square brackets and 95% confidence intervals in brackets. IRRs are defined as the number of exposed events (e.g. number of locum shifts) divided by the number of unexposed events (offset – e.g. permanent doctor FTE) in each time period and are essentially a ratio of two incidence rates. An IRR with a value greater than 1 indicates that the incident rate is higher in an exposed group compared to an unexposed group and the opposite is true for an IRR value less than 1. We focused on effect sizes rather than P values since statistical significance is more likely and can be less meaningful in large datasets such as the one we analysed. (15) Sensitivity analyses where we excluded ambulance and community trusts and examined the effects of deprivation on our three outcomes were nearly identical to the results from the main analyses. Deprivation did not appear to have any discernible effect on any of the three outcomes. The results from the sensitivity analyses are provided in supplementary table 4 and the absolute differences in shift coverages for the statistically significant coefficients are provided in supplementary table 5.

Table 2: Negative binomial regression analyses for the three outcomes in 2019, IRR [a,b]

	**Locum Intensity**	**Agency shifts**	**Unfilled shifts**

For peer review only - http://bmjopen.bmj.com/site/about/guidelines.xhtml

**Trust level aggregate FTE**<br><br>**(reference group is quintile 1)**	**Reference group**	**Reference group**	**Reference group**
Quintile 2	0.784 (0.527 to 1.676), <0.231 [0.159]	0.945 (0.734 to 1.218), <0.662 [0.122]	0.936 (0.449 to 1.952), <0.859 [0.351]
Quintile 3	0.496 (0.299 to 0.825)*, <0.007 [0.129]	0.937 (0.675 to 1.301), <0.698 [0.157]	1.848 (0.735 to 4.645), <0.192 [0.869]
Quintile 4	0.611 (0.349 to 1.072), <0.086 [0.175]	0.883 (0.617 to 1.264), <0.497 [0.162]	1.878 (0.704 to 5.011), <0.208 [0.940]
Quintile 5	0.347 (0.187 to 0.644)*, <0.001 [0.110]	0.796 (0.530 to 1.195), <0.271 [0.165]	2.447 (0.826 to 7.251), <0.106 [1.356]
**Trust type**<br><br>**(reference group is NHS general acute trust)**	**Reference group**	**Reference group**	**Reference group**
NHS specialist trust	0.285 (0.174 to 0.468)*, <0.001 [0.072]	1.510 (1.086 to 2.100)*, <0.014 [0.254]	0.233 (0.091 to 0.598)*, <0.002 [0.112]
Mental health trust	0.966 (0.628 to 1.486), <0.875 [0.212]	1.576 (1.198 to 2.073)*, <0.001 [0.220]	1.062 (0.508 to 2.221), <0.873 [0.400]
Ambulance service	55.43 (20.56 to 149)*, <0.001 [27.96]	0.033 (0.008 to 0.147)*, <0.001 [0.025]	3.894 (0.453 to 33.14), <0.215 [4.272]
Community service	1.443 (0.780 to 2.670), <0.243 [0.453]	0.962 (0.641 to 1.445), <0.854 [0.199]	1.360 (0.471 to 3.930), <0.570 [0.736]
**CQC ratings**<br><br>**(reference group is trusts with good and outstanding services)**	**Reference group**	**Reference group**	**Reference group**
Inadequate and requiring improvement	1.495 (1.191 to 1.877)*, <0.001 [0.173]	1.044 (0.907 to 1.201), <0.550 [0.075]	1.193 (0.789 to 1.804), <0.402 [0.251]
**Trust level substantive doctor turnover rates**	1.015 (1.009 to 1.021)*, <0.001 [0.002]	1.001 (0.997 to 1.003), <0.589 [0.001]	0.995 (0.987 to 1.003), <0.248 [0.004]
**Trust level vacancy rates [full-time equivalent (FTE)]**	1.000 (0.999 to 1.001), <0.530 [0.005]	0.999 (0.999 to 1.001), <0.948 [0.001]	0.999 (0.997 to 1.001), <0.585 [0.001]

For peer review only - http://bmjopen.bmj.com/site/about/guidelines.xhtml

Region (reference region is London)	Reference group	Reference group	Reference group
South West	0.575 (0.361 to 0.915)*, <0.019 [0.136]	1.447 (1.098 to 1.907)*, <0.009 [0.204]	0.687 (0.316 to 1.493), <0.343 [0.272]
South East	0.701 (0.472 to 1.041), <0.078 [0.141]	1.349 (1.047 to 1.736)*, <0.021 [0.175]	0.524 (0.252 to 1.092), <0.085 [0.196]
Midlands	1.041 (0.714 to 1.520), <0.832 [0.201]	1.425 (1.126 to 1.804)*, <0.003 [0.172]	0.548 (0.276 to 1.086), <0.085 [0.191]
East of England	0.813 (0.533 to 1.240), <0.336 [0.175]	1.525 (1.167 to 1.993)*, <0.002 [0.208]	0.402 (0.182 to 0.890)*, <0.025 [0.163]
North West	1.045 (0.705 to 1.550), <0.826 [0.210]	1.327 (1.035 to 1.701)*, <0.026 [0.168]	0.855 (0.412 to 1.773), <0.673 [0.318]
North East and Yorkshire	0.754 (0.495 to 1.150), <0.191 [0.162]	1.449 (1.120 to 1.875)*, <0.005 [0.191]	0.575 (0.269 to 1.230), <0.154 [0.223]
constant	0.030 (0.152 to 0.601)*, <0.001 [0.105]	0.436 (0.283 to 0.671)*, <0.001 [0.096]	0.117 (0.038 to 0.357)*, <0.001 [0.066]

[a] Model A included data on 220 trusts (observation) while models B and C included data on 214 trusts with robust standard errors.

[b] Coefficients can be interpreted as proportionate changes, for example, trusts in the North West had on average 4.5% higher locum intensity than trusts in London.

Locum intensity

Results indicate that in 2019 trust size was a strong predictor of locum intensity. Using quintile 1 (i.e. small trust size) as the reference group, our results showed significant reductions in locum intensity for medium and very large trusts with IRRs of 0.496 [0.299 to 0.825 95% CI] for quintile 3, and 0.347 [0.187 to 0.644) for quintile 5. As an example of interpretation, comparing quintile 1 and quintile 3 suggests a locum intensity 50.4% lower for the medium size trusts. This was equivalent to 228.1 fewer weekly locum shifts for medium size trusts. NHS specialist trusts had a 71.5% lower locum intensity (IRR=0.285; 95% CI 0.174 to 0.468) than NHS general acute trusts and this effect was equivalent to 152.8 fewer weekly locum shifts for NHS specialist trusts. Ambulance service trusts had 55 times higher locum intensity than NHS general acute (IRR=55.43; 95% CI 20.56 to 149). However, this result is an artefact of the very low numbers of permanent doctors employed by ambulance trusts and the very small number of locum shifts filled when compared to other trust types. CQC ratings were strongly associated with higher locum intensity with trusts rated as inadequate or required improvement having 49.5% (IRR=1.495; 95% CI 1.191 to 1.877) higher mean locum intensity or 84.5 more weekly locum shifts than trusts rated good or outstanding. Staff turnover rates had negligible effects on locum intensity (IRR=1.015; 95% CI 1.009 to 1.021). Trusts in the

For peer review only - http://bmjopen.bmj.com/site/about/guidelines.xhtml

South West had 40.25% lower locum intensity than trusts in London (IRR=0.575; 95% CI 0.361 to 0.915).

Proportion of agency shifts

NHS specialist trusts and mental health trusts had 51% (IRR=1.510; 95% CI 1.086 to 2.100) and 57.6% (IRR=1.576; 95% CI 1.198 to 2.07) higher proportion of agency shifts than NHS general acute trusts. These effects were equivalent to 48.2 and 54.8 more weekly agency shifts for NHS specialist and mental health trusts, respectively. Ambulance service trusts had 96.7% lower proportion of agency shifts (IRR=0.033; 95% CI 0.008 to 0.147) than NHS general acute trusts. Trusts in the East of England had the highest proportion of agency shifts compared to trusts in London (IRR=1.525; 95% CI 1.167 to 1.993). The effects of trust size on the proportion of agency shifts were not statistically significant.

Proportion of unfilled shifts

NHS specialist trusts had 76.7% lower proportion of unfilled shifts when compared to NHS general acute trusts and this was equivalent to 23.77 fewer weekly unfilled shifts for NHS specialist trusts. Trusts in the East of England had 59.80 lower rates of unfilled shifts when compared to trusts in London (IRR=0.402; [0.182 to 0.890 95% CI]).

Locum use during the COVID-19 pandemic

Figure 3 shows the mean agency, bank, unfilled and total shifts per week at the trust level in 2019 to 2021. Over time, the trust level mean was 188.5 shifts per week (SD=205.8), of which 95.2 (SD=108.6) were agency shifts and 93.3 (SD=135.8) were bank staff shifts and the mean of unfilled shifts across all trusts was 38.5 (SD =85.2). Pre-pandemic, we observed small variability in the mean number of agency, bank and unfilled shifts. In March 2020 there was a steep decline (approximately 18%) in agency and bank shifts per trust as very few trusts reported locum use between March and April. In the third quarter of 2020, we observed an increase (approximately 15%) in agency and bank shifts per trust. In 2021, there was a steep steady increase in the mean number of unfilled shifts from 33.9 to 50.1 (47.8% increase) between May and June, which was sustained throughout 2021 and reached a peak of 69.2 unfilled shifts per trust in December 2021.

Discussion

For peer review only - http://bmjopen.bmj.com/site/about/guidelines.xhtml

Summary

This study provides evidence on the extent of locum use and factors associated with locum use in NHS trusts in England for the period 2019 – 2021. Our findings show that on average 4.4% of medical staffing in NHS trusts in 2019 was provided by locum medical staffing. Trusts with lower CQC ratings, acute trusts and smaller trusts had higher locum intensity. We observed moderate variability in locum use across regions and greater variability in the proportion of shifts filled by agency locums. During 2021, the proportion of shifts that were unfilled reached a three year high. Our findings can help inform NHS organisations about the extent of their locum use and provide for the first time important information about the drivers of locum use across NHS trusts. This can help with the effective planning of the NHS workforce by providing a better understanding of the make-up and spread of the locum medical workforce in England to aid recruitment in underperforming areas.

Strengths and limitations of the study

The main strength of this study is the national scope and coverage of every NHS trust of England. For the first time, using routinely collected data on locum use, we quantified the extent of locum use, sourced from agencies or banks, across all NHS trusts for the period 2019-2021. We also explored whether trusts were able to cover sufficiently for staff shortages and identified drivers of locum use at the trust level for the whole of England. We provide evidence on the extent of locum use across NHS Trusts during the COVID-19 pandemic. Our analyses allowed us to control for measured trust and population characteristics.

However, this study has some important limitations which should be considered when interpreting the key findings. First, the NHS Improvement data do not reveal information on locum use by specialty and there may be substantial variations across specialties which we could not identify. Second, although NHS Improvement collects data on the number of locum shifts, it does not collect the shift duration, locum FTE or the number of shifts filled by permanent doctors which would allow a more straightforward comparison with permanent doctor FTE. Therefore, we had to assume that shift lengths for permanent and locum doctors were broadly equivalent in order to estimate the proportion of medical staffing provided by locum doctors. Should data on the number of shifts filled by permanent doctors or data on locum FTE become available, this limitation could be addressed. Third, there may also exist variability in locum use between locums of different types (e.g. infrequent or long-term locums) or durations apart from the agency/bank categories, which has been observed in general practice. (9) Some locums may be employed for several months (16) often to cover a vacancy which has not been filled, while others may cover short-term absences such as illness for as little as one or two shifts and we did not have that information. Fourth, the dataset has no information on how well NHS trusts use their locum workforce such as the provision of adequate induction, training, supervision and feedback in accordance with NHSE guidance.

For peer review only - http://bmjopen.bmj.com/site/about/guidelines.xhtml

Prior work (17) suggests that locum performance is driven more by organisational attributes such as these than by the characteristics of the locum doctors themselves. Fifth, the data do not contain any information on costs for locum doctors and we were therefore unable to estimate the extra financial costs of using agency locum to fill shifts.

Interpretation of findings

The use of locum doctors is important because of the high level of spending it entails and because of concerns about the quality and safety of locum staffing arrangement. (18) Our study shows that the actual level of locum use, as a proportion of overall medical staffing, is relatively low on average, but varies considerably, with some trusts having much higher use of locums and some trusts relying overly on more expensive agency locums rather than using staff banks.

Some of this variation may be explained by organisational characteristics. For instance, larger trusts may be more able to cover workforce gaps within their own staff without needing locums, and specialist/tertiary trusts may find it easier to recruit and provide attractive workplaces compared with general acute trusts. Mental health trusts may face particular staffing shortages, which may explain the high level of agency locum use.

Our results show significantly higher locum intensity in trusts with worse CQC ratings (inadequate or requires improvement). It may be that these trusts find it harder to recruit and fill workforce gaps, but it could also be hypothesised that sustained high levels of locum use may impact quality and safety and hence affect CQC ratings.

The introduction of the first UK lockdown brought significant reductions in the numbers of both bank and agency locum doctors employed across NHS trusts, due to cancelations in elective care. However, shortly after, trusts started employing more locums likely in an effort to tackle excessive workload and increasing demand for health care services during the pandemic. Furthermore, in 2021, we observed an increase in the mean number of shifts filled by bank compared to the previous years and this was accompanied with a stable trend in agency shifts and an increase in the number of unfilled shifts. This suggests that trusts were meeting the increased demand with bank staff, which is in line with the new agency rules enacted by NHS Improvement in 2019 that aim to reduce reliance on agency staff. (19) Despite the increase in the mean number of total shifts, trusts appeared to be less able to fill the number of shifts they were requesting over the second half of 2021. This may suggest a persisting high workload for permanent doctors that trusts were unable to address with the use of locum doctors over that period.

For peer review only - http://bmjopen.bmj.com/site/about/guidelines.xhtml

Contributors: CG, TA and KW designed the study. CG extracted the data from all sources, performed the analyses and drafted the manuscript. KW, EK, JF, GS, DA and TA critically revised the manuscript. CG is the guarantor of this work and, as such, had full access to all the data in the study and takes responsibility for the integrity of the data and the accuracy of the data analysis.

Data availability statement: Data may be obtained from a third party and are not publicly available. Data can be obtained from NHS Improvement under a special license and are not freely available.

Patient consent for publication: Not required.

Acknowledgments: We would like to thank NHS Improvement and NHS Digital for the wealth of information they have collected and systematically organised, which made this study possible.

Competing interests: The authors have declared no competing interests.

Ethics Approval: No ethics approval was required as the study uses aggregated data.

Funding: This study was funded by the National Institute for Health Research (NIHR) Health Service and Delivery Research programme (project reference number: NIHR128349). The views expressed are those of the authors and not necessarily those of the NIHR or the Department of Health and Social Care.

For peer review only - http://bmjopen.bmj.com/site/about/guidelines.xhtml

References

1. Rimmer A. NHS England workforce plan fails to tackle staff shortages, say health leaders. BMJ. 2020;370:m3057; 2020.
2. Kings Fund. NHS workforce: our position. London King's Fund. 2021.
3. Buchan J, Charlesworth A, Gershlick B, Seccombe I. A critical moment: NHS staffing trends, retention and attrition. The Health Foundation. 2019.
4. Anderson M, O'Neill C, Macleod Clark J, Street A, Woods M, Johnston-Webber C, et al. Securing a sustainable and fit-for-purpose UK health and care workforce. Lancet. 2021;397(10288):1992-2011.
5. Focus on physicians; census of consultant physicians and higher specialty trainees 2018-19. London. Royal College of Physicians. 2019.
6. NHS Improvement. Reducing reliance on agency staff: consultation response. 2019.
7. Department of Health. The Department of Health and Social Care's written evidence to the Review Body on Doctors' and Dentists' Remuneration (DDRB) for the 2020/21 Pay Round. Department of Health. 2020.
8. NHS Digital. General Practice Workforce. 2019.
9. Grigoroglou C, Walshe K, Kontopantelis E, Ferguson J, Stringer G, Ashcroft DM, et al. Locum doctor use in English general practice: analysis of routinely collected workforce data 2017–2020. Br J Gen Prac. 2022;72(715):e108-e17.
10. NHS Professionals. About NHS Professionals. 2022.
11. NHS Digital. NHS workforce statistics. 2019.
12. Care Quality Commission. Using CQC data. 2020.
13. NHS Digital. Hospital Episode Statistics. 2017.
14. Digital N. NHS Vacancy Statistics. NHS Digital. 2021.
15. Lin MF, Lucas HC, Shmueli G. Too big to fail: large samples and the p-value problem. Inform Syst Res2013;24:906-17.
16. The British Medical Association. Locum working for SAS doctors. London. 2022.
17. Ferguson J, Walshe K. The quality and safety of locum doctors: a narrative review. J R Soc Med. 2019;112(11):462-71.
18. Ferguson J, Tazzyman A, Walshe K, Bryce M, Boyd A, Archer J, et al. 'You're just a locum': professional identity and temporary workers in the medical profession. Sociol Health Illn. 2021;43(1):149-66.
19. NHS Improvement. Agency rules. 2019

For peer review only

For peer review only - http://bmjopen.bmj.com/site/about/guidelines.xhtml

Fig 1: Locum intensity, proportion of agency shifts and proportion of unfilled shifts in 2019, NHS trust level

a)Mean locum intensity (bank and agency shifts combined) at trust level in 2019†*

b)Mean proportion of agency shifts at trust level in 2019††

c)Mean proportion of unfilled shifts at trust level in 2019¥

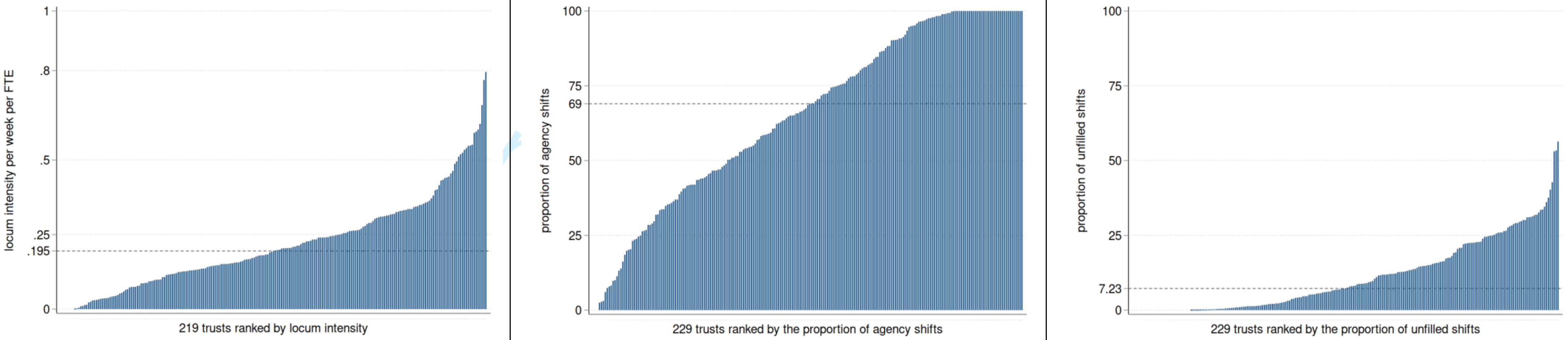

† dash line indicates the median (0.195) locum intensity per week perFTE across 219 NHS trusts in 2019

* 10 trusts were excluded from the analyses due to very small or zero denominators (i.e. low permanent doctor FTE)

†† dash line indicates the median (69%) proportion of agency shifts per week across 229 NHS trusts in 2019

¥ dash line indicates the median (7.23%) unfilled shift rate per week across 229 NHS trusts in 2019.

Fig 2: Regional variation in locum intensity, proportion of agency shifts and proportion of unfilled shifts, 2019*

a) Regional variation in locum intensity (bank + agency shifts combined) in 2019†**

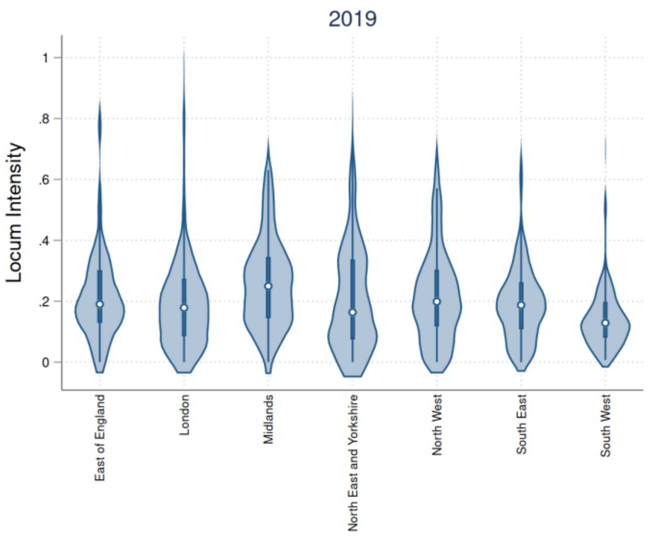

† Figure includes data from 222 NHS trusts in 2019, adjusted for permanent doctor FTE.

b) Regional variation in the proportion of agency shifts in 2019††

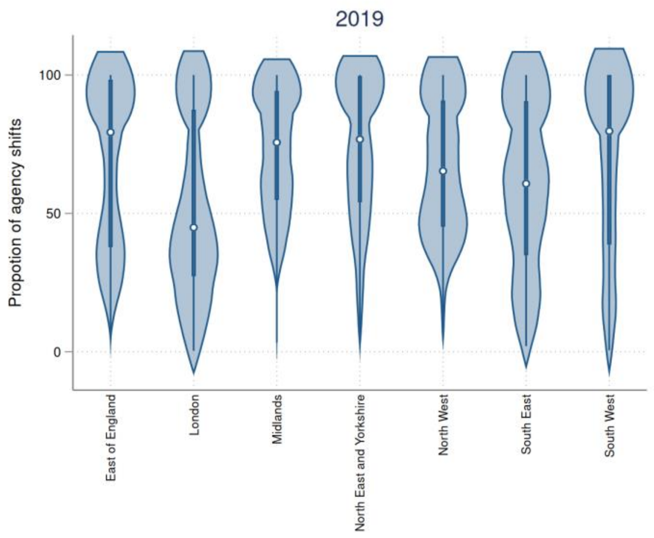

†† Figure includes data from 229 NHS trusts in 2019

c) Regional variation in the proportion of unfilled shifts in 2019¥

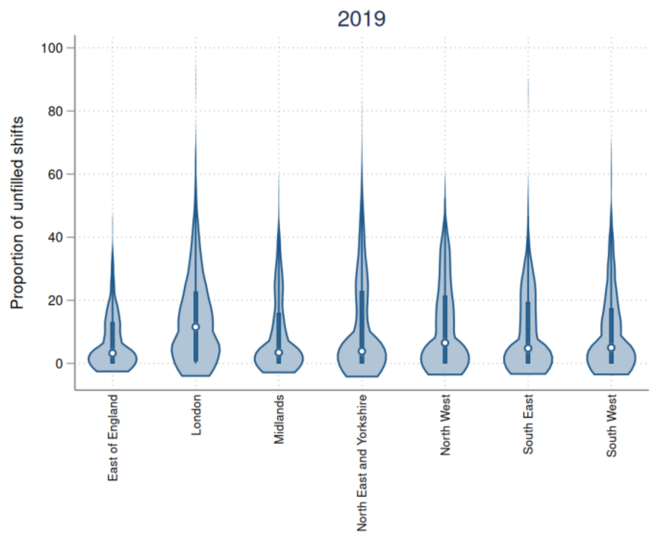

¥ Figure includes data from 229 NHS trusts in 2019

* The thick blue line represents the interquartile range (25th–75th centile) and the thin line represents the rest of the distribution with upper/lower adjacent values. The white dot represents the median of the data. The distribution shape of the data is based on a kernel density estimation where wider sections of the plot represent a higher chance that members of the population of interest will take on a given value and where thinner section represent lower chance.

**Seven trusts were excluded from the analyses due to non-availability of data for permanent doctor FTE.

Fig 3: Variation in mean number of locum shifts over time, 2019 – 2021*±

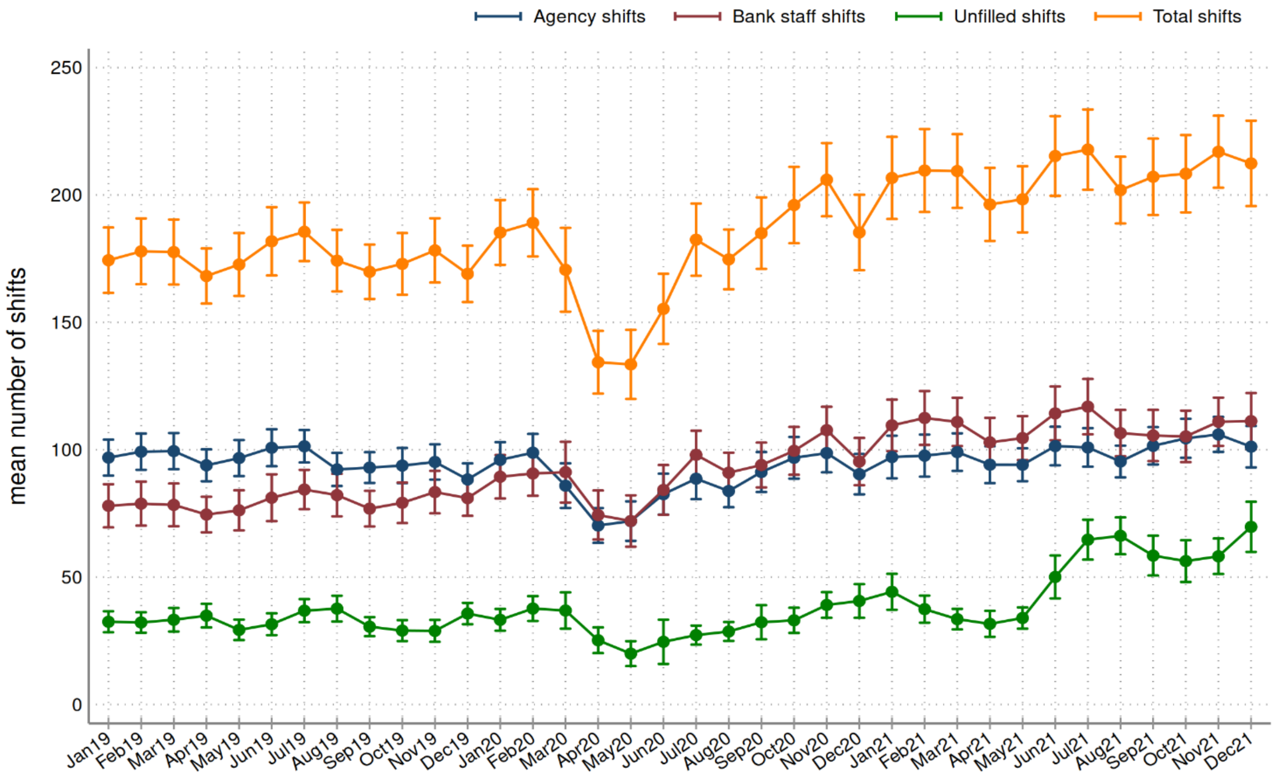

*dots indicate the mean number of shifts across trusts for each month in the period 2019 – 2021. and the vertical lines represent the upper and lower 95% confidence limits
± The number of trusts that reported data in 2019, 2020 and 2021 were 229, 224 and 221 respectively.

Supplementary material - The use of locum doctors in NHS Trusts in England. Analysis of routinely collected workforce data 2019 – 2021.

For peer review only

Contents

Additional databases ... 3

NHS Workforce statistics database ... 3

NHS Vacancy statistics ... 3

Health Regulators Ratings ... 3

English Indices of Deprivation ... 4

Spatial Maps ... 4

Tables ... 5

$Table 1: NHS Trusts with the highest and lowest locum intensity in England, 2019 ... 5

$Table 2 - Descriptive statistics in 2020, by region*† ... 6

$Table 3 - Descriptive statistics in 2021, by region*† ... 7

$Table 4: Negative binomial regression analyses for the three outcomes in 2019, acute and mental health trusts, IRR a,b,c ... 8

$Table 5: Adjusted marginal effects for the three outcomes in 2019, acute and mental health trusts, a,b ... 10

Figures ... 11

$Figure 1: Locum intensity, proportion of agency shifts and proportion of unfilled shifts in 2019, 2020 & 2021 - NHS Trust level ... 11

$Figure 2: Regional variation in locum intensity, proportion of agency shifts and proportion of unfilled shifts, 2019, 2020 & 2021 - NHS Trust level* ... 12

$Figure 3: Spatial map of locum intensity at the Sustainability and Transformation Partnerships (STP) level, England 2019 ... 13

$Figure 4: Spatial map of agency shifts at the Sustainability and Transformation Partnerships (STP) level, England 2019 ... 14

$Figure 5: Spatial map of unfilled shifts at the Sustainability and Transformation Partnerships (STP) level, England 2019 ... 15

References ... 16

For peer review only

Additional databases

NHS Workforce statistics database

NHS Digital collects monthly NHS Hospital and Community Health Service (HCHS) workforce statistics (1) for staff in NHS trusts and Clinical Commissioning Groups (CCGs) in England. The data are recorded within the Electronic Staff Record (ESR), which is a payroll and human resources system and contains staff records NHS employed staff in England since 2008. Data are available as headcount and full-time equivalents and for all months from September 2009 onwards and they represent an accurate summary of the validated data extracted from the NHS ESR system. We downloaded monthly data collections available from NHS Digital from December 2017 to December 2021. For each month and each NHS trust, we calculated the average total FTE across all available doctor categories in the NHS workforce database and matched the data to NHS improvement data for each trust in each month. The database also included monthly NHS trust level turnover data which were used in the negative binomial regression.

NHS Vacancy statistics

From NHS Digital, (2) we obtained trust level vacancy rates for the period January 2019 to December 2019. The series refers to vacancy FTE from providers which are available on a quarterly basis but recorded on a monthly basis. This monthly rate is defined as the total number of unfilled posts reported at the end of each respective reporting month. A vacancy is defined as a post that is unfilled by permanent or fixed-term staff. Some vacant posts may be filled by agency or temporary staff, but these posts are still considered to be vacancies. The number of vacancies is the difference between the number of reported full-time equivalent (FTE) permanent or fixed-term staff in post and planned workforce levels (i.e. the total funded or budgeted establishment on an FTE basis).

Health Regulators Ratings

Health regulators ratings were obtained from the Care Quality Commission (CQC). Each rating is based on the assessment of the evidence against the key lines of enquiry in the assessment framework for healthcare services and, for relevant non-specialist acute trusts, the use of resources assessment framework. The trust level ratings refer to the trust's overall quality, based on findings under five key quality questions that CQC inspects (safe, effective, caring, responsive and well-led). Where applicable, the CQC also awards a combined rating at the trust level, based on the findings of the five trust-level quality ratings plus a use of resources rating. (3, 4) Each year the CQC inspects NHS trusts,

For peer review only

and awards each of them one of four rating levels ('outstanding', 'good', 'requires improvement', or 'inadequate') in five domains ('safe', 'effective', 'responsive', 'caring', and 'well led'), along with an 'overall' rating that summarised the domain ratings. From the CQC, we obtained published inspection ratings and corresponding inspection dates for all 229 NHS trusts in 2019 that were inspected between January 2019 and December 2019. Over the inspection cycle, some NHS trusts were re-inspected and their ratings updated. Only the rating from the first inspection was used in this study, as subsequent ratings were likely to have been influenced by the previous inspection process and outcomes. Data on the most recent practice inspections are freely available online. (5)

English Indices of Deprivation

Area deprivation, as measured by the latest update of the Index of Multiple Deprivation (IMD) (i.e.2019) was available at the 2011 Lower Super Output (LSOA) level. The IMD measures relative levels of deprivation for all the 32,844 LSOAs in England on a continuous scale of deprivation where most of the indicators are based on 2012 statistics. It is a combined score of deprivation based on a total of 37 separate indicators that have been grouped into seven domains, each of which reflects a different aspect of deprivation experienced by individuals living in an area. The overall measure is calculated as a weighted mean across seven domains: income, employment, education and skills, health and disability, crime, barriers to housing and services, and living environment with different weights given to each domain. The Index of Multiple Deprivation is assigned to every small area in England and ranks them from 1 (most deprived area) to 32,844 (least deprived area). The IMD is widely used across central government to focus programmes on the most deprived areas. (6) These area measures were assigned to trust based on trusts' location. Trusts in our analysis are allocated an IMD score based on the mean IMD score of all admitted patients using data from Hospital Episode Statistics 2017. (7)

Spatial Maps

Digital vector boundaries for the 2019 STPs (Sustainability and Transformation Partnerships), generalised to 20 metres and clipped to the coastline to reduce size and improve visualisation, were obtained from the ONS open geography portal. (8) The vector boundaries were inputted in the Stata shp2dta (9) command to calculate the centroid for each STP in the British National Grid format. These were then converted from British National Grid easting and northing to longitude and latitude in degrees. (10)

For peer review only

Tables

$Table 1: NHS Trusts with the highest and lowest locum intensity in England, 2019

**Trusts with the highest locum intensity in England**	**Locum intensity***	**Trusts with the lowest locum intensity in England**	**Locum intensity***
North East London NHS Foundation Trust	0.795	Royal Papworth Hospital NHS Foundation Trust	0.001
Bedfordshire Hospitals Foundation Trust	0.768	Cambridgeshire Community Services NHS Trust	0.002
Rotherham Doncaster And South Humber NHS Foundation Trust	0.684	The Newcastle Upon Tyne Hospitals NHS Foundation Trust	0.004
Oxford Health NHS Foundation Trust	0.621	Moorfields Eye Hospital NHS Foundation Trust	0.009
Dudley And Walsall Mental Health Partnership NHS Trust	0.602	Alder Hey Children's NHS Foundation Trust	0.010
North Cumbria University Hospitals NHS Trust	0.595	North Tees And Hartlepool NHS Foundation Trust	0.014
George Eliot Hospital NHS Trust	0.591	University Hospitals Bristol And Weston NHS Foundation Trust	0.014
North Cumbria Integrated Care NHS Foundation Trust	0.551	Sheffield Children's NHS Foundation Trust	0.022
United Lincolnshire Hospitals NHS Trust	0.548	Leeds Community Healthcare NHS Trust	0.025
Pennine Acute Hospitals NHS Trust	0.548	Imperial College Healthcare NHS Trust	0.029

*Locum intensity is defined as the sum agency and bank locum shifts adjusted for the size of Trusts' permanent doctor workforce

For peer review only

$Table 2 - Descriptive statistics in 2020, by region*†

	East of England	London	Midlands	North East & Yorkshire	North West	South East	South West
Locum intensity *, Median (25th–75th centile)	**0.21 (0.11 to 0.29)**	**0.17 (0.08 to 0.27)**	**0.23 (0.11 to 0.37)**	**0.16 (0.06 to 0.29)**	**0.19 (0.10 to 0.34)**	**0.18 (0.06 to 0.28)**	**0.11 (0.08 to 0.18)**
Proportion of agency shifts (%) Median (25th–75th centile)	**62.2 (32 to 94.4)**	**42.1 (18.7 to 77.5)**	**74.1 (47.2 to 95.1)**	**63.5 (44.4 to 100)**	**57.2 (36.1 to 93.9)**	**50 (24.3 to 94.7)**	**67.4 (33 to 100)**
Proportion of unfilled shifts † (% of requested shifts), Median (25th–75th centile)	**4.87 (0 to 16)**	**12 (1.15 to 21.7)**	**2.3 (0 to 14.4)**	**4 (0 to 21.1)**	**6.7 (0 to 18.7)**	**5.6 (0 to 18)**	**5 (0 to 20.9)**
Full-time doctor FTE, Median (25th–75th centile)	628.7 (288.3 to 1,344.9)	846 (379.3 to 2,102.7)	593.2 (233.2 to 1,347.8)	715.6 (269 to 1,479.1)	603.2 (309.2 to 1,032.1)	1,037.6 (293.9 to 1,392.8)	743.2 (240.9 to 1,249)
Trust types							
NHS general acute trusts (n)	16	18	20	21	20	17	17
Acute - NHS specialist trusts (n)	1	5	3	1	6	1	-
Mental health trusts (n)	4	10	12	8	6	5	6
Community health (n)	3	2	4	1	2	5	-
Ambulance service (n)	1	1	2	2	1	2	1

$Table 3 - Descriptive statistics in 2021, by region*†

	East of England	London	Midlands	North East & Yorkshire	North West	South East	South West
Locum intensity *, Median (25th–75th centile)	**0.19 (0.09 to 0.28)**	**0.19 (0.09 to 0.31)**	**0.29 (0.15 to 0.40)**	**0.18 (0.07 to 0.33)**	**0.23 (0.13 to 0.39)**	**0.22 (0.14 to 0.30)**	**0.12 (0.10 to 0.19)**
Proportion of agency shifts (%) Median (25th–75th centile)	**55.4 (31 to 87.7)**	**35.8 (18.5 to 68.5)**	**70.1 (45.5 to 93.6)**	**60.3 (39.4 to 100)**	**61.5 (33.9 to 88)**	**39.6 (21.6 to 66.6)**	**55.4 (33.6 to 100)**
Proportion of unfilled shifts † (% of requested shifts), Median (25th–75th centile)	**7.66 (0 to 17.9)**	**17 (6.9 to 27.1)**	**3.7 (0 to 17.2)**	**9.6 (0 to 22.9)**	**11.9 (0 to 27.7)**	**13.1 (0 to 22)**	**8.8 (0 to 28.9)**
Full-time doctor FTE, Median (25th–75th centile)	644.5 (34.6 to 1,222.1)	776.4 (50.6 to 1,685.4)	344.3 (33.3 to 1,222.5)	654.1 (2.19 to 1,248.5)	534.2 (218.1 to 1,047.1)	438.4 (95.6 to 1,332.6)	636.7 (134.1 to 1,354.7)
Trust types							
NHS general acute trusts (n)	14	18	19	23	18	17	16
Acute - NHS specialist trusts (n)	1	5	3	1	6	1	-
Mental health trusts (n)	6	8	12	10	6	5	5
Community health (n)	3	2	4	1	2	5	-
Ambulance service (n)	1	1	1	4	-	2	1

$Table 4: Negative binomial regression analyses for the three outcomes in 2019, sensitivity analyses with acute and mental health trusts only, IRR a,b,c

	Locum Intensity	Agency shifts	Unfilled shifts
**Trust level aggregate FTE**<br><br>**(reference group is quintile 1)**	**Reference group**	**Reference group**	**Reference group**
Quintile 2	0.499 (0.369 to 0.676), <0.001 (0.077)	1.044 (0.811 to 1.344), <0.735 (0.134)	2.189 (0.967 to 4.960), <0.060 (0.913)
Quintile 3	0.465 (0.321 to 0.676), <0.001 (0.088)	1.044 (0.760 to 1.434), <0.787 (0.169)	3.076 (1.179 to 8.023), <0.022 (1.504)
Quintile 4	0.486 (0.329 to 0.716), <0.001 (0.096)	0.937 (0.671 to 1.311), <0.708 (0.160)	2.572 (0.937 to 7.063), <0.067 (1.325)
Quintile 5	0.248 (0.157 to 0.390), <0.001 (0.057)	0.859 (0.574 to 1.287), <0.462 (0.177)	3.783 (1.237 to 11.572), <0.020 (2.158)
**Trust type**<br><br>**(reference group is NHS non-specialist trust)**	**Reference group**	**Reference group**	**Reference group**
NHS specialist trust	0.254 (0.176 to 0.366), <0.001 (0.047)	1.600 (1.153 to 2.220), <0.005 (0.268)	0.321 (0.119 to 0.866), <0.025 (0.162)
Mental health trust	0.991 (0.643 to 1.233), <0.487 (0.147)	1.556 (1.180 to 2.052), <0.002 (0.219)	1.015 (0.457 to 2.256), <0.971 (0.414)
**CQC ratings**<br><br>**(reference group is trusts that provide good and outstanding services)**	**Reference group**	**Reference group**	**Reference group**
Inadequate and requiring improvement	1.626 (1.365 to 1.938), <0.001 (0.145)	1.039 (0.899 to 1.201), <0.601 (0.077)	1.329 (0.855 to 2.066), <0.206 (0.299)
**Index of multiple deprivation**			

For peer review only

**(reference group is quintile 1, where 1 is the most deprived)**			
Quintile 2	1.072 (0.818 to 1.406), <0.610 (0.148)	1.077 (0.859 to 1.349), <0.520 (0.124)	0.585 (0.308 to 1.111), <0.101 (0.191)
Quintile 3	1.138 (0.855 to 1.514), <0.375 (0.166)	1.182 (0.936 to 1.494), <0.159 (0.141)	0.494 (0.248 to 0.986), <0.046 (0.174)
Quintile 4	1.153 (0.855 to 1.554), <0.350 (0.175)	1.198 (0.933 to 1.539), <0.156 (0.153)	0.948 (0.448 to 2.008), <0.890 (0.363)
Quintile 5	1.038 (0.741 to 1.455), <0.827 (0.179)	1.169 (0.882 to 1.550), <0.278 (0.168)	0.628 (0.286 to 1.381), <0.247 (0.252)
**Region (reference region is London)**	**Reference group**	**Reference group**	**Reference group**
South West	0.468 (0.328 to 0.668), <0.001 (0.085)	1.595 (1.198 to 2.123), <0.014 (0.233)	1.258 (0.510 to 3.103), <0.618 (0.580)
South East	0.746 (0.531 to 1.048), <0.092 (0.130)	1.498 (1.136 to 1.975), <0.006 (0.211)	0.578 (0.262 to 1.273), <0.174 (0.233)
Midlands	0.980 (0.718 to 1.337), <0.896 (0.155)	1.481 (1.149 to 1.908), <0.001 (0.192)	0.795 (0.371 to 1.700), <0.553 (0.308)
East of England	0.815 (0.579 to 1.147), <0.240 (0.142)	1.632 (1.233 to 2.161), <0.001 (0.234)	0.419 (0.177 to 0.988), <0.047 (0.184)
North West	0.917 (0.646 to 1.301), <0.627 (0.163)	1.289 (0.964 to 1.724), <0.101 (0.191)	1.176 (0.508 to 2.724), <0.704 (0.504)
North East and Yorkshire	0.703 (0.491 to 1.005), <0.053 (0.128)	1.450 (1.084 to 1.941), <0.001 (0.215)	0.753 (0.299 to 1.901), <0.549 (0.356)
constant	0.381 (0.221 to 0.656), <0.001 (0.105)	0.339 (0.219 to 0.524), <0.001 (0.075)	0.065 (0.017 to 0.247), <0.001 (0.045)

[a] All models included data on 187 trusts (observations) with robust standard errors.
[b] 95% confidence intervals are in brackets; results are reported as incidence rate ratios (IRR) followed by *P*-values and standard errors in parentheses.
[c] Coefficients can be interpreted as proportionate changes, for example, trusts in the North West had on average 8.3% lower locum intensity than trusts in London.

For peer review only

$Table 5: Adjusted marginal effects for the three outcomes in 2019, acute and mental health trusts, IRR a,b

	Total shifts	Agency shifts	Unfilled shifts
**CQC rates**			
Good + outstanding	170.5 (130 to 211.1), <0.001 [20.7]	-	-
Requiring improvement + inadequate	255 (197.1 to 312.9), <0.001 [29.6]	-	-
**Trust type**			
NHS non-specialist	213.8 (168.9 to 258.6), <0.001 [22.9]	94.1 (86 to 102.3), <0.001 [4.17]	31 (22.6 to 39.4), <0.001 [4.31]
NHS specialist trust	61 (31.5 to 90.6), <0.001 [15.1]	142.2 (98.3 to 186), <0.001 [22.4]	7.23 (0.53 to 14), <0.001 [3.42]
Mental health trust	206.5 (122.7 to 290.3), <0.001 [42.8]	148.3 (112.4 to 184.3), <0.001 [18.3]	-
Ambulance service	11,829.7 (1,372.9 to 22,286.5), <0.001 [5,335.2]	3.2 (-1.51 to 7.82], <0.185 [2.4]	-
Community service	308.4 (127.5 to 489.3), <0.001 [92.3]	-	-
**Trust size**			
Quintile 1	452.9 (201.3 to 704.6), <0.001 [128.3]	-	-
Quintile 2	355.3 (196.8 to 513.8), <0.001 [80.9]	-	-
Quintile 3	224.8 (150.9 to 298.7), <0.001 [37.7]	-	-
Quintile 4	276.9 (193 to 360.8), <0.001 [42.8]	-	-
Quintile 5	157.3 (111.2 to 203.5), <0.001 [23.6]	-	-

[a] Model A included data on 220 trusts (observation) while models B and C included data on 214 trusts with robust standard errors.

[b] Coefficients can be interpreted as absolute changes, for example, trusts that were rated as inadequate and requiring improvement on average had 84.5 more weekly locum shifts than trusts that were rated as having good and outstanding services.

For peer review only

Figures

$Figure 1: Locum intensity, proportion of agency shifts and proportion of unfilled shifts in 2019, 2020 & 2021 - NHS Trust level

a)Mean locum intensity in 2019†*

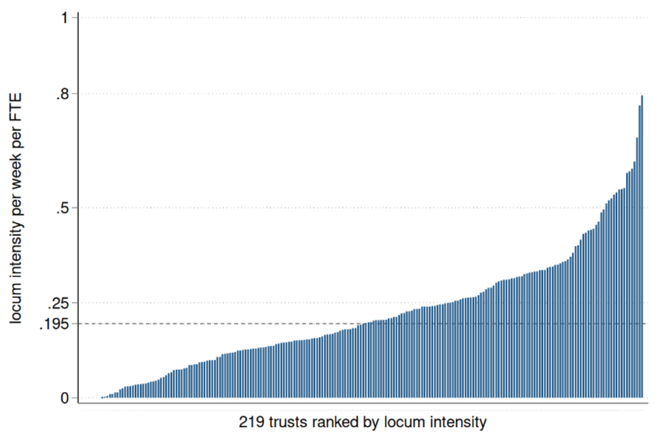

† dash line indicates the median (0.195) locum intensity per week per FTE across 219 NHS trusts in 2019.

d)Mean proportion of agency shifts in 2019††

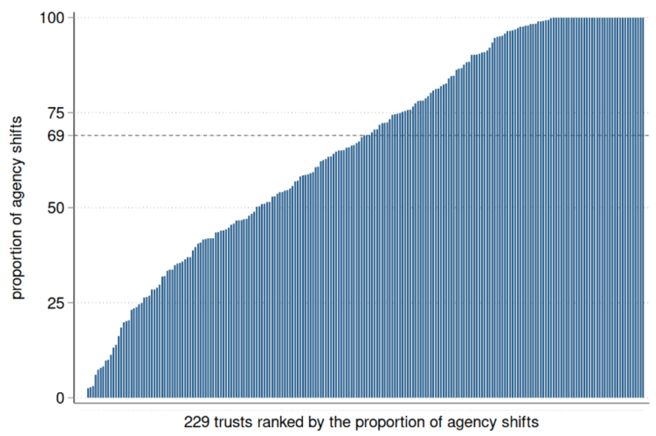

†† dash line indicates the median (69%) proportion of agency shifts perweek across 229 NHS trusts in 2019.

g)Mean proportion of unfilled shifts in 2019¥

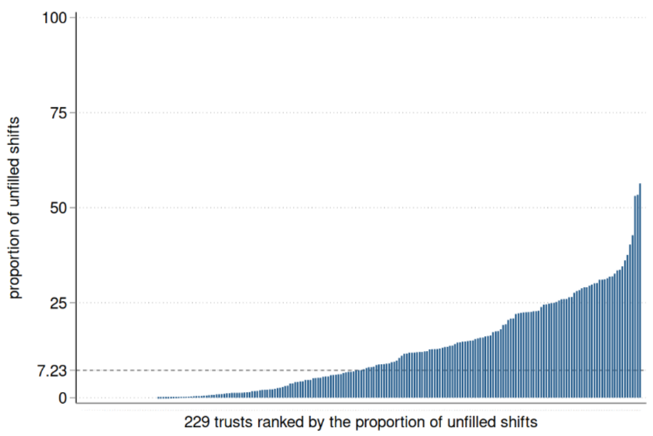

¥ dash line indicates the median (7.23%) unfilled shift rate per week across 229 NHS trusts in 2019.

b)Mean locum intensity in 2020†*

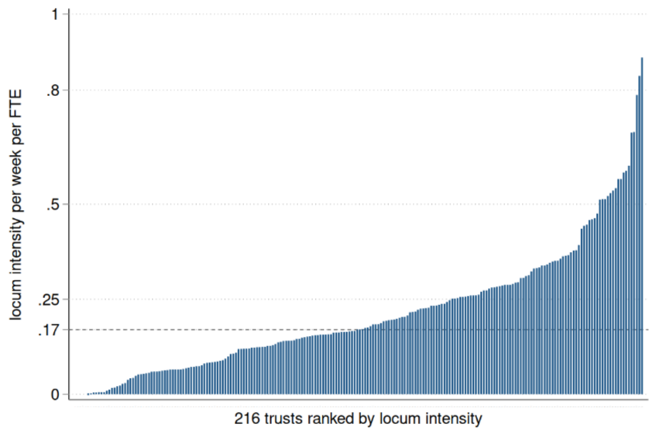

† † dash line indicates the median (0.170) locum intensity per week per FTE across 216 NHS trusts in 2020.

e)Mean proportion of agency shifts in 2020††

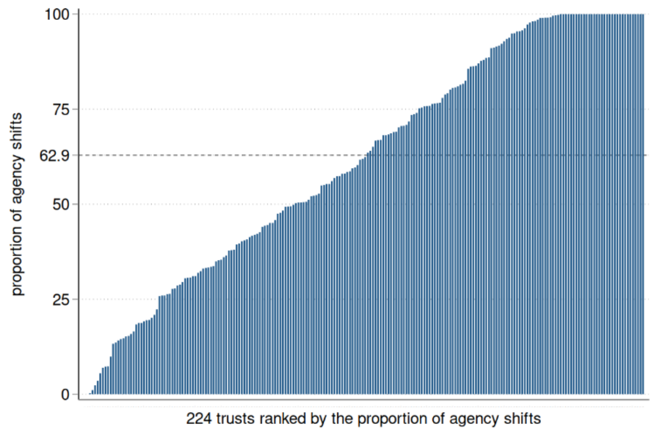

†† dash line indicates the median (62.9%) proportion of agency shifts per week across 224 NHS trusts in 2020.

h)Mean proportion of unfilled shifts in 2020¥

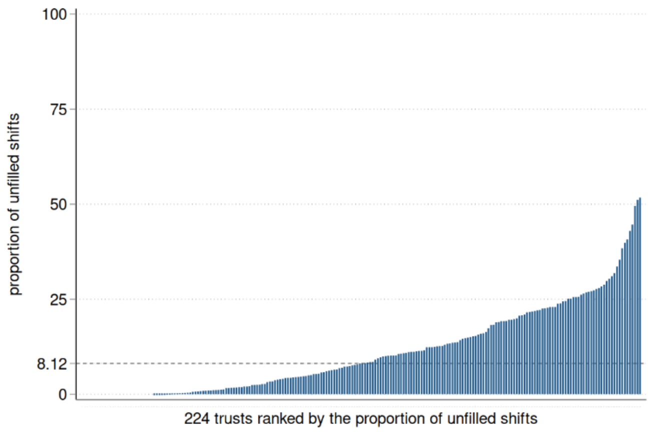

¥ dash line indicates the median (8.12%) unfilled shift rate per week across 224 NHS trusts in 2020.

c)Mean locum intensity in 2021†*

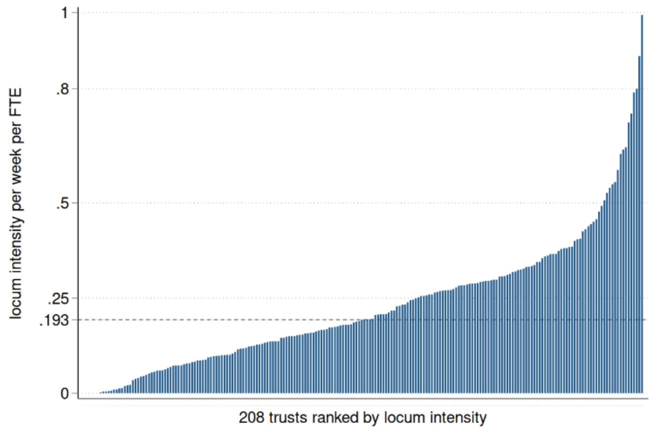

† dash line indicates the median (μ=0.193) locum intensity per week per FTE across 208 NHS trusts in 2021.

f)Mean proportion of agency shifts in 2021††

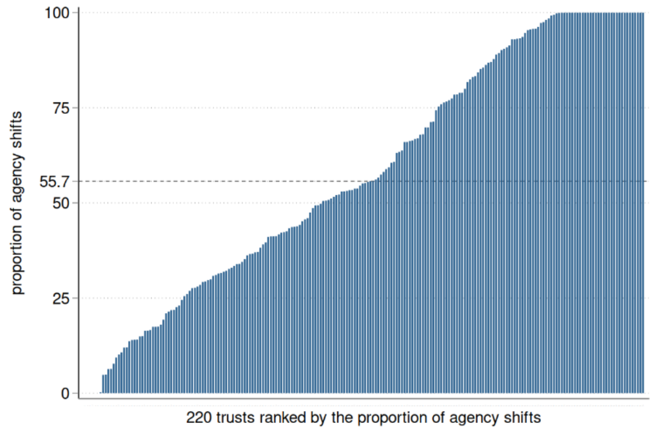

†† dash line indicates the median (μ=55.7%) proportion of agency shifts per week across 220 NHS trusts in 2021.

i)Mean proportion of unfilled shifts in 2021¥

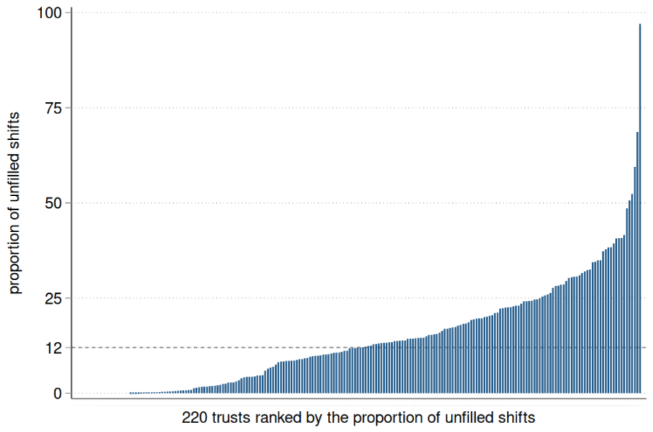

¥ dash line indicates the median (μ=12%) unfilled shift rate per week across 220 NHS trusts in 2021.

*Ten, eight and twelve trusts were excluded from the analyses due to very small or zerodenominators (i.e. low permanent doctor FTE) in 2019, 2020 and 2021, respectively.

$Figure 2: Regional variation in locum intensity, proportion of agency shifts and proportion of unfilled shifts, 2019, 2020 & 2021 - NHS Trust level*

a) Regional variation in locum intensity in 2019†**

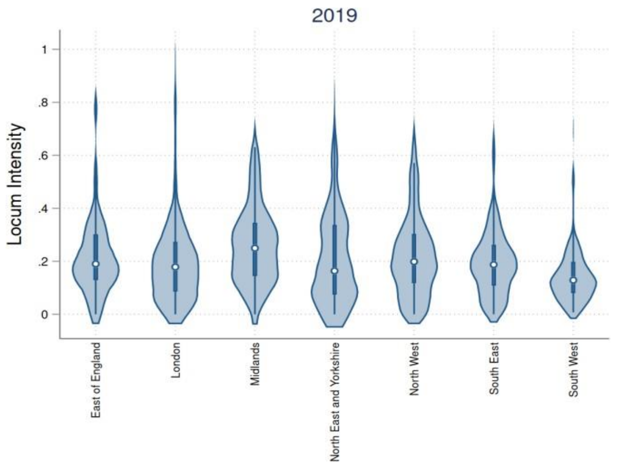

† Figure includes data from 222 NHS trusts in 2019

b) Regional variation in the proportion of agency shifts in 2019††

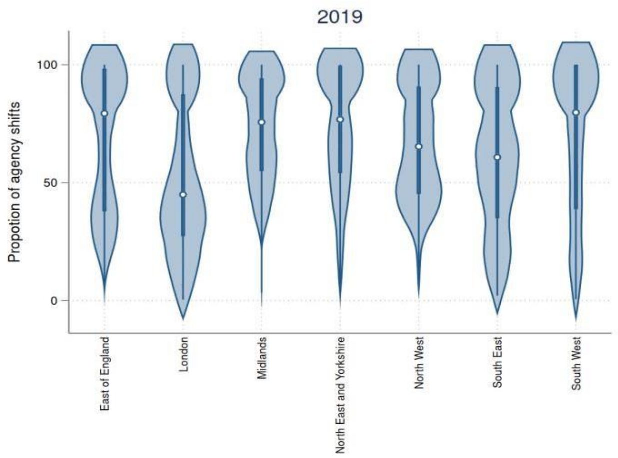

†† Figure includes data from 229 NHS trusts in 2019

c) Regional variation in the proportion of unfilled shifts in2019¥

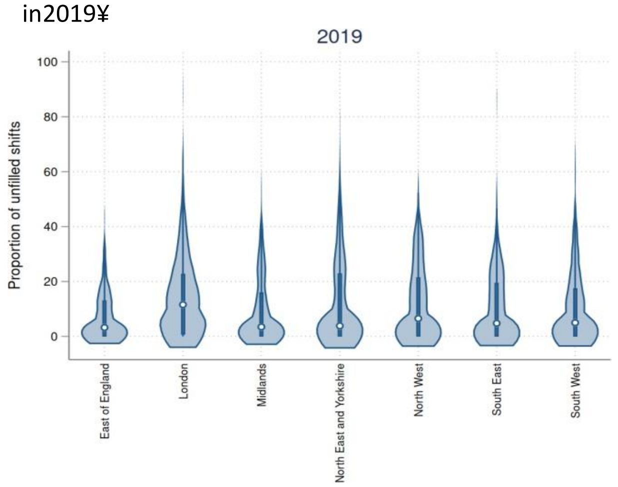

¥ Figure includes data from 229 NHS trusts in 2019

d) Regional variation in locum intensity in 2020†**

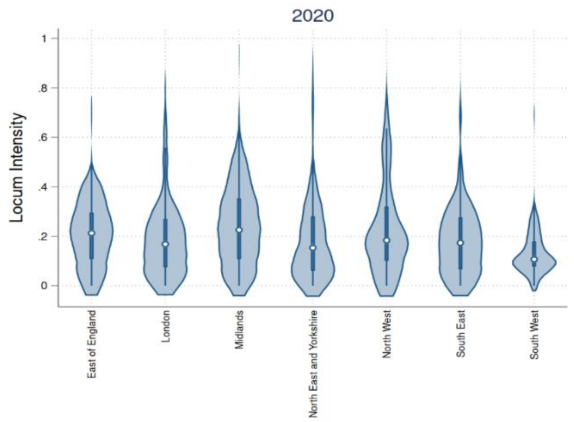

† Figure includes data from 218 NHS trusts in 2020

e) Regional variation in the proportion of agency shifts in 2020††

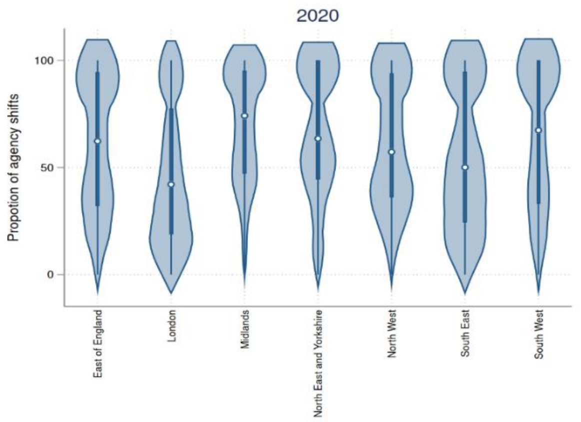

†† Figure includes data from 224 NHS trusts in 2020

f) Regional variation in the proportion of unfilled shifts in 2020¥

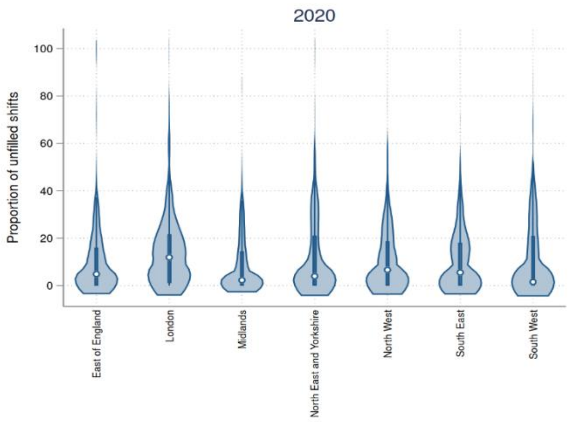

¥ Figure includes data from 224 NHS trusts in 2020

g) Regional variation in locum intensity in 2021†**

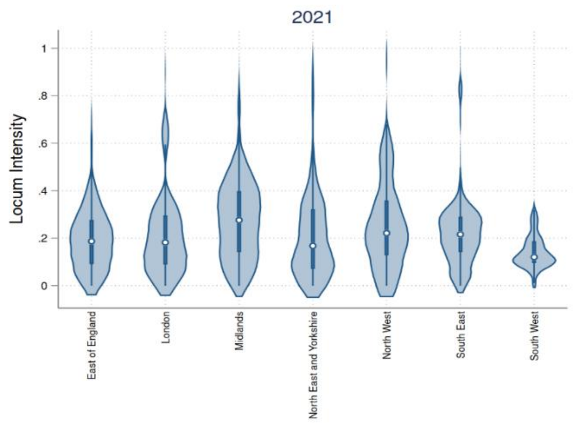

† Figure includes data from 210 NHS trusts in 2021

h) Regional variation in the proportion of agency shifts in 2021††

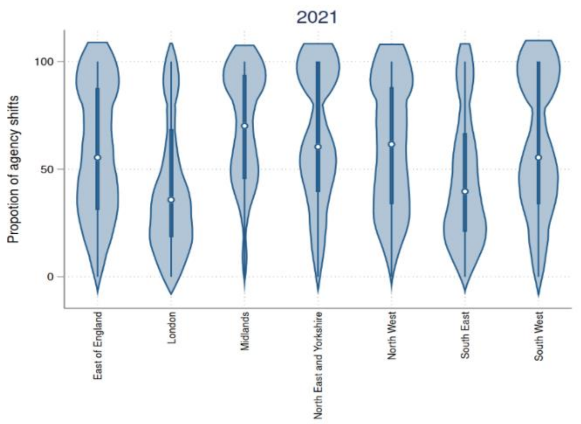

†† Figure includes data from 221 NHS trusts in 2021

i) Regional variation in the proportion of unfilled shifts in 2021¥

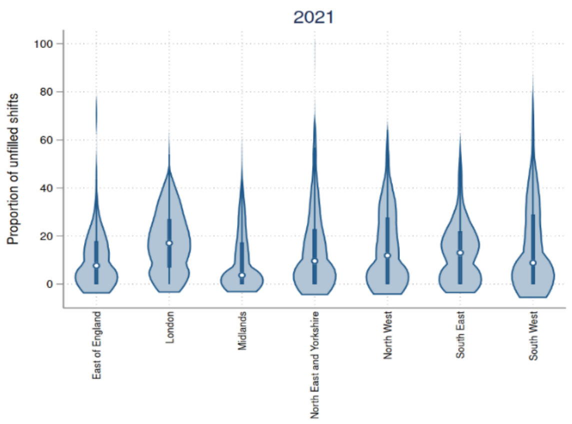

¥ Figure includes data from 221 NHS trusts in 2021

* The thick blue line represents the interquartile range (25th–75th centile) and the thin line represents the rest of the distribution with upper/lower adjacent values. The white dot represents the median of the data.The distribution shape of the data is based on a kernel density estimation where wider sections of the plot represent a higher chance that members of the population of interest will take on a given value and where thinner section represent lower chance.

**Seven, six and eleven trusts were excluded from the analyses due to non-availability of data for permanent doctor FTE in 2019, 2020 and 2021, respectively. Locum intensity is adjusted for permanent doctor FTE.

$Figure 3: Spatial map of locum intensity at the Sustainability and Transformation Partnerships (STP) level, England 2019

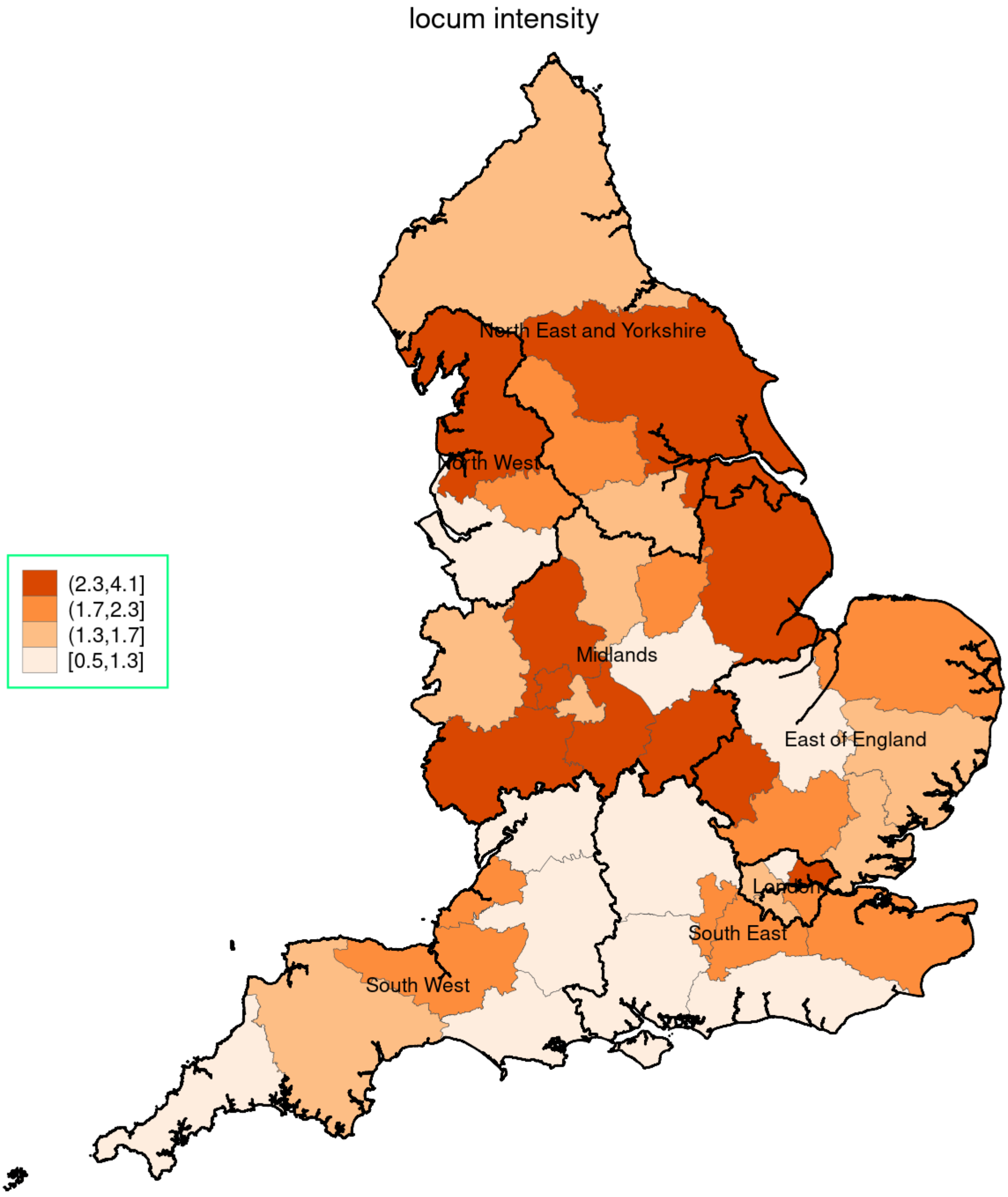

$Figure 4: Spatial map of agency shifts at the Sustainability and Transformation Partnerships (STP) level, England 2019

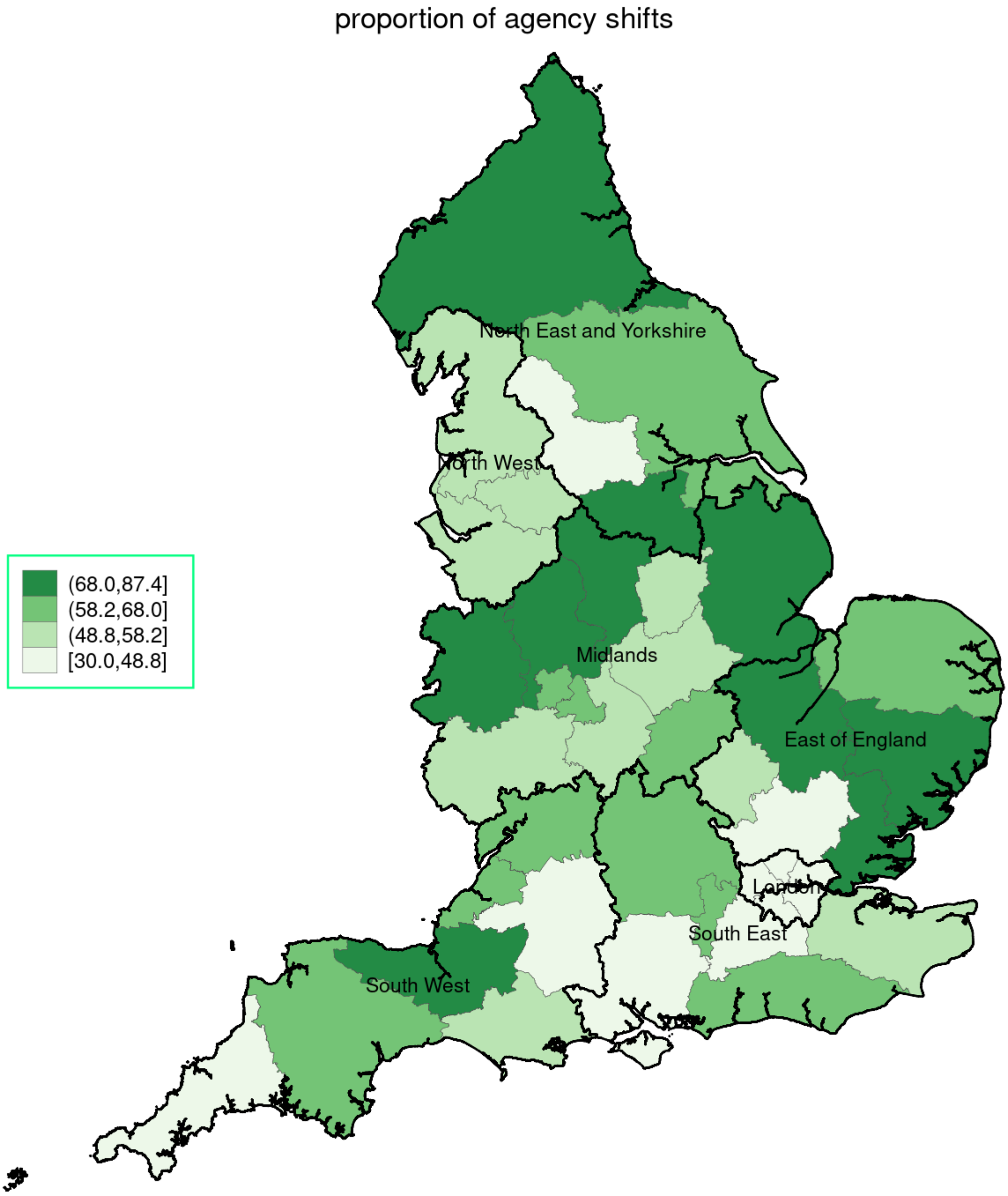

$Figure 5: Spatial map of unfilled shifts at the Sustainability and Transformation Partnerships (STP) level, England 2019

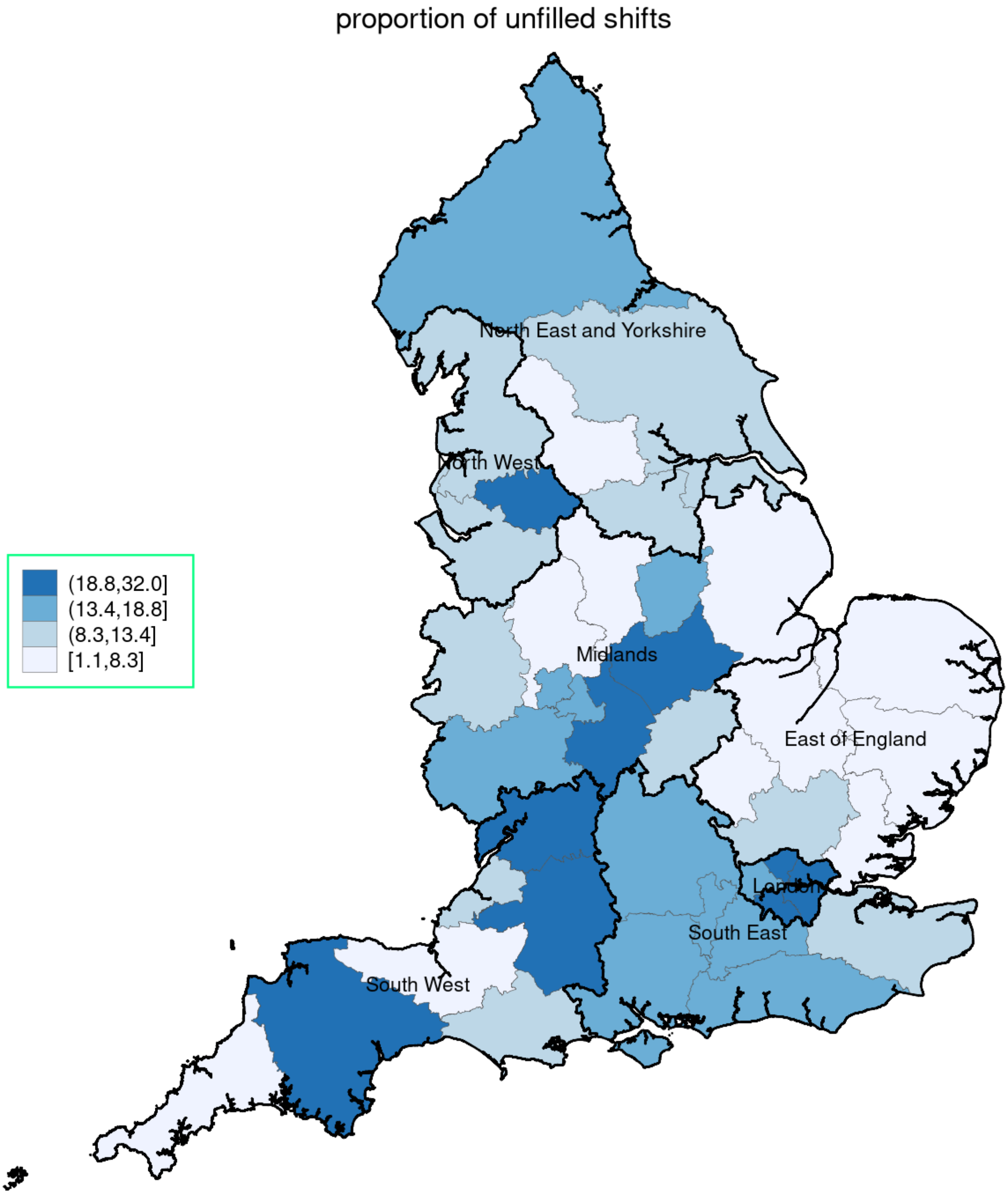

References

1. NHS Digital. NHS workforce statistics. 2022.
2. NHS Digital. NHS Vacancy Statistics. 2021.
3. Care Quality Commission. Shaping the Future: CQC's strategy 2016 to 2021. 2016.
4. Care Quality Commission. Intelligent Monitoring: NHS GP practices Indicators and methodology. 2014.
5. Care Quality Commission. Using CQC data. 2020.
6. Ministry of Housing, Communities and Local GovernmenT. The English Indices of Deprivation. Ministry of Housing, Communities and Local Government 2019.
7. NHS Digital. Hospital Episode Statistics. 2017.
8. Office for National Statistics. Open Geography Portal. 2018.
9. SHP2DTA: Stata module to convert shape boundary files to Stata datasets[program]. Boston College Department of Economics. 2006.;S456718 version.
10. Ordance Survey. A guide to coordinate systems in Great Britain. ONS, 2013:43. www.ordnancesurvey.co.uk/docs/support/guide-coordinate-systems-great-britainpdf.

For peer review only

The RECORD statement – checklist of items, extended from the STROBE statement, that should be reported in observational studies using routinely collected health data.

	Item No.	STROBE items	Location in manuscript where items are reported	RECORD items	Location in manuscript where items are reported
**Title and abstract**					
	1	(a) Indicate the study's design with a commonly used term in the title or the abstract (b) Provide in the abstract an informative and balanced summary of what was done and what was found	2	RECORD 1.1: The type of data used should be specified in the title or abstract. When possible, the name of the databases used should be included.<br><br>RECORD 1.2: If applicable, the geographic region and timeframe within which the study took place should be reported in the title or abstract.<br><br>RECORD 1.3: If linkage between databases was conducted for the study, this should be clearly stated in the title or abstract.	2<br><br>2<br><br>N/A
**Introduction**					
Background rationale	2	Explain the scientific background and rationale for the investigation being reported	4		
Objectives	3	State specific objectives, including any prespecified hypotheses	4		
**Methods**					
Study Design	4	Present key elements of study design early in the paper	4-5		
Setting	5	Describe the setting, locations, and relevant dates, including periods of recruitment, exposure, follow-up, and data collection	5		

For peer review only

Participants	6	*(a) Cohort study* - Give the eligibility criteria, and the sources and methods of selection of participants. Describe methods of follow-up<br>*Case-control study* - Give the eligibility criteria, and the sources and methods of case ascertainment and control selection. Give the rationale for the choice of cases and controls<br>*Cross-sectional study* - Give the eligibility criteria, and the sources and methods of selection of participants<br><br>*(b) Cohort study* - For matched studies, give matching criteria and number of exposed and unexposed<br>*Case-control study* - For matched studies, give matching criteria and the number of controls per case	5	RECORD 6.1: The methods of study population selection (such as codes or algorithms used to identify subjects) should be listed in detail. If this is not possible, an explanation should be provided.<br><br>RECORD 6.2: Any validation studies of the codes or algorithms used to select the population should be referenced. If validation was conducted for this study and not published elsewhere, detailed methods and results should be provided.<br><br>RECORD 6.3: If the study involved linkage of databases, consider use of a flow diagram or other graphical display to demonstrate the data linkage process, including the number of individuals with linked data at each stage.	N/A - whole population study<br><br>N/A<br><br>N/A
Variables	7	Clearly define all outcomes, exposures, predictors, potential confounders, and effect modifiers. Give diagnostic criteria, if applicable.	6	RECORD 7.1: A complete list of codes and algorithms used to classify exposures, outcomes, confounders, and effect modifiers should be provided. If these cannot be reported, an explanation should be provided.	The data are under a special agreement and only members of the research team can access the data.
Data sources/ measurement	8	For each variable of interest, give sources of data and details of methods of assessment (measurement).<br>Describe comparability of assessment methods if there is more than one group	5-6		

For peer review only

Bias	9	Describe any efforts to address potential sources of bias	7		
Study size	10	Explain how the study size was arrived at	N/A		
Quantitative variables	11	Explain how quantitative variables were handled in the analyses. If applicable, describe which groupings were chosen, and why	6-7		
Statistical methods	12	(a) Describe all statistical methods, including those used to control for confounding<br>(b) Describe any methods used to examine subgroups and interactions<br>(c) Explain how missing data were addressed<br>(d) *Cohort study* - If applicable, explain how loss to follow-up was addressed<br>*Case-control study* - If applicable, explain how matching of cases and controls was addressed<br>*Cross-sectional study* - If applicable, describe analytical methods taking account of sampling strategy<br>(e) Describe any sensitivity analyses	7<br>7<br>7<br>7<br>7		
Data access and cleaning methods		..N/A		RECORD 12.1: Authors should describe the extent to which the investigators had access to the database population used to create the study population.	N/A

For peer review only

				RECORD 12.2: Authors should provide information on the data cleaning methods used in the study.	
Linkage		..N/A		RECORD 12.3: State whether the study included person-level, institutional-level, or other data linkage across two or more databases. The methods of linkage and methods of linkage quality evaluation should be provided.	N/A
**Results**					
Participants	13	(a) Report the numbers of individuals at each stage of the study (*e.g.*, numbers potentially eligible, examined for eligibility, confirmed eligible, included in the study, completing follow-up, and analysed)<br>(b) Give reasons for non-participation at each stage.<br>(c) Consider use of a flow diagram	8<br><br>N/A<br><br>N/A	RECORD 13.1: Describe in detail the selection of the persons included in the study (*i.e.,* study population selection) including filtering based on data quality, data availability and linkage. The selection of included persons can be described in the text and/or by means of the study flow diagram.	N/A whole population study
Descriptive data	14	(a) Give characteristics of study participants (*e.g.*, demographic, clinical, social) and information on exposures and potential confounders<br>(b) Indicate the number of participants with missing data for each variable of interest<br>(c) *Cohort study* - summarise follow-up time (*e.g.*, average and total amount)	8<br><br>8<br><br>N/A		
Outcome data	15	*Cohort study* - Report numbers of outcome events or summary measures over time<br>*Case-control study* - Report numbers in each exposure			

		category, or summary measures of exposure<br>*Cross-sectional study* - Report numbers of outcome events or summary measures	8		
Main results	16	(a) Give unadjusted estimates and, if applicable, confounder-adjusted estimates and their precision (e.g., 95% confidence interval). Make clear which confounders were adjusted for and why they were included<br>(b) Report category boundaries when continuous variables were categorized<br>(c) If relevant, consider translating estimates of relative risk into absolute risk for a meaningful time period	9-10<br><br>N/A<br><br>N/A		
Other analyses	17	Report other analyses done—e.g., analyses of subgroups and interactions, and sensitivity analyses	10		
**Discussion**					
Key results	18	Summarise key results with reference to study objectives	11-13		
Limitations	19	Discuss limitations of the study, taking into account sources of potential bias or imprecision. Discuss both direction and magnitude of any potential bias	14	RECORD 19.1: Discuss the implications of using data that were not created or collected to answer the specific research question(s). Include discussion of misclassification bias, unmeasured confounding, missing data, and changing eligibility over time, as they pertain to the study being reported.	N/A
Interpretation	20	Give a cautious overall interpretation of results considering objectives,	15-16		

		limitations, multiplicity of analyses, results from similar studies, and other relevant evidence			
Generalisability	21	Discuss the generalisability (external validity) of the study results	16		
**Other Information**					
Funding	22	Give the source of funding and the role of the funders for the present study and, if applicable, for the original study on which the present article is based	13		
Accessibility of protocol, raw data, and programming code		..		RECORD 22.1: Authors should provide information on how to access any supplemental information such as the study protocol, raw data, or programming code.	N/A

*Reference: Benchimol EI, Smeeth L, Guttmann A, Harron K, Moher D, Petersen I, Sørensen HT, von Elm E, Langan SM, the RECORD Working Committee. The REporting of studies Conducted using Observational Routinely-collected health Data (RECORD) Statement. *PLoS Medicine* 2015; in press.

*Checklist is protected under Creative Commons Attribution (CC BY) license.
